# Randomized controlled trial to test the efficacy of a brief, communication-based, substance use preventive intervention for parents of adolescents: Protocol for the SUPPER Project (Substance Use Prevention Promoted by Eating family meals Regularly)

**DOI:** 10.1371/journal.pone.0263016

**Published:** 2022-02-02

**Authors:** Margie R. Skeer, Rachael A. Sabelli, Katherine M. Rancaño, Michelle Lee-Bravatti, Emma C. Ryan, Misha Eliasziw, Anthony Spirito

**Affiliations:** 1 Tufts University School of Medicine, Boston, MA, United States of America; 2 Friedman School of Nutrition Science and Policy, Tufts University, Boston, MA, United States of America; 3 Warren Alpert Medical School of Brown University, Providence, RI, United States of America; PLOS: Public Library of Science, UNITED KINGDOM

## Abstract

**Background:**

Substance use among adolescents in the U.S. is associated with adverse physical and mental health outcomes in the long-term. Universal youth-focused substance use prevention programs have demonstrated effectiveness but are often not sustainable due to the significant amount of time, effort, and resources required. We describe a trial protocol for a brief, low-participant-burden intervention to improve substance use-specific parent-child communication through the promotion of family meals and increased parental engagement.

**Methods:**

This study is a parallel-group randomized controlled trial designed to assess the efficacy of a 13-week intervention. A total of 500 dyads of parents and their 5^th^-7^th^ grade children are recruited from across Massachusetts. Dyads are randomized to the intervention or attention-control condition using block urn randomization, based on child grade, gender, and school. Parents/guardians in the substance use preventive intervention arm receive a short handbook, attend two meetings with an interventionist, and receive two SMS messages per week. Parents/guardians in the control arm receive the same dose but with content focused on nutrition, physical activity, and weight stigma. Participant dyads submit videos of family meals, audio recordings of prompted conversations, and quantitative surveys over an 18-month period (baseline, 3, 6, 12, 18 months post-intervention). The primary outcomes measure the quantity and quality of parent-child substance use conversations and proximal child indicators (i.e., substance use attitudes and expectancies, affiliation with substance-using peers, and intentions and willingness to use substances). The secondary outcome is child substance use initiation.

**Discussion:**

This is a novel, brief, communication-focused intervention for parents/guardians that was designed to reduce participant burden. The intervention has the potential to improve parent-child engagement and communication and conversations about substance use specifically and decrease child substance use risk factors and substance use initiation.

**Trial registration:**

ClinicalTrials.gov NCT03925220. Registered on 24 April 2019.

## Background

Substance use during adolescence in the U.S. is a major public health problem, as the earlier a child initiates alcohol use, the greater their risk for developing substance use disorders [1,2]. Approximately 90% of those with substance-related problems started using before the age of 18 [3], and compared to those aged 18–25, youth aged 12–17 who use substances are more likely to develop disordered use within a year [4]. While substance use among high school students has been on the decline in recent years, it is still highly prevalent [5,6]. Data from the 2019 Youth Risk Behavior Surveillance System indicated that nationally, 19.0% of ninth graders had at least one drink of alcohol in the last thirty days and 24.3% and 18.1% had ever used marijuana and cigarettes, respectively [7]. The prevalence of these risky behaviors is higher among twelfth-grade students, as 39.9% had at least one drink of alcohol in the last thirty days and 48.7% and 32.0% had ever used marijuana and cigarettes, respectively [7]. Current vaping patterns in high school students have steeply inclined–more than a quarter of high school students currently vape compared to 11.7% of high schoolers in 2017 [8,9].

Given the stress and increased mental health problems among youth [10], the COVID-19 pandemic has raised some concerns regarding adolescent substance use, although evidence is limited [11,12]. One study of Canadian adolescents reported the proportion of adolescents who reported one or more substance-using days within a period of three weeks pre-COVID compared to 3 weeks during COVID increased significantly from 0.76 to 0.96 (p = 0.02) and from 0.94 to 1.10 (p = 0.01) for alcohol and marijuana, respectively [13]. The adverse consequences of adolescent substance use include an increased likelihood of sexual risk taking, driving while intoxicated, and delinquency [14,15]. According to the National Institute on Drug Abuse, the prevention of substance use among youth is a national public health priority and the COVID-19 pandemic has further exacerbated the problem [16].

There is a significant need for brief, family-based, substance use prevention programs. During adolescence, the family environment has been recognized as having a profound influence on substance use [17–19]. Universal substance use prevention programs (i.e., programs aimed at the general population without regard for individual level of risk [20]) that include parents have been shown to be efficacious [21]. However, the programs that have been most effective are resource-intensive and require extensive time and effort for program staff and participants [21]. Therefore, an approach to universal substance use prevention is needed that reduces participant and program staff burden and is effective, easily implemented and disseminated, and sustainable.

Family meals can be a conduit of parent-child communication, which has been associated with decreased substance use in adolescents [22]. In observational studies, the practice of having family meals is consistently associated with a reduced risk of tobacco, alcohol, and other drug use, as well as with other behavioral problems [23–25]. However, to the best of our knowledge, no study has examined whether encouraging family meals while building communication skills in parents is an effective strategy for substance use prevention.

The SUPPER Project (Substance Use Prevention Promoted by Eating family meals Regularly) addresses this gap in knowledge; it is a brief, communication-focused, universal prevention program that utilizes eating family meals and parent-child communication as primary preventive strategies for adolescent substance use initiation. This program uses the “brief intervention model” [26,27] to target parents/guardians (referred to “parents” herein) as facilitators in preventing substance use among their children with a framework that is easily adaptable, has low participant burden, and is resource efficient, thus enhancing sustainability. A SUPPER pilot study (R34 DA031337; PI: Skeer) demonstrated that the project was feasible and acceptable to families and preliminarily efficacious [28]. In the current randomized controlled trial we aim to: 1) determine the efficacy of The SUPPER Project on parent outcomes including higher quality and frequency of parent-child conversations about substance use; 2) examine the effects of the prevention program on proximal child indicators including attitudes and expectancies regarding substance use, engagement with substance-using peers, and intentions and willingness to use substances; and 3) examine the effects of the prevention program on pre-/early adolescent substance use. If shown to be efficacious, the SUPPER Project would offer a brief, evidence-based, low-burden program for families.

## Methods/Design

The SUPPER Project employs a parallel group randomized controlled trial design. The study aims to enroll a diverse sample of 500 parent-child dyads. Dyads are randomized, with a 1:1 allocation, to receive either the substance use prevention (experimental) condition or an attention-control (comparison) condition focused on nutrition, physical activity, and weight stigma. Dyads are recruited from public and private schools in the Greater Boston area of Massachusetts. As this is a universal preventive intervention, all families in participating schools who meet eligibility criteria (see ‘Screening and Eligibility’ below) are able to participate. To enhance external validity, an administrative diversity supplement (R01DA045073-02S1) allowed us to additionally focus recruitment efforts on ethnically and racially diverse communities, as well as those with heightened risk factors (e.g., higher prevalence of substance use disorders, gang involvement, etc.). Due to the differences in administration and infrastructure between school districts and individual schools, the process for building relationships with each school varies. In general, school districts are selected based upon their proximity to the Boston-based study team due to the in-person activities involved and their interest in being involved in the research. Permission is first received at a district level to conduct research within the schools. Schools within the district are then contacted and those interested are asked to sign a formal letter of support. Individualized recruitment plans are made with each school utilizing their existing communication platforms (e.g. newsletters, school social media, teacher-parent emails or text messages) and opportunities for in-person meetings with parents (e.g. open houses, school events, parent-teacher conferences) to disseminate information about the study. Information about the study is available to all students; however, schools are given the flexibility to target recruitment to best serve the needs of their communities. A recruitment goal is communicated to each school and progress toward that goal is reported back regularly, which is based on the number of students in each target grade. Interested parents reach out to the study team directly to complete the screening and eligibility process.

In March 2020, the original recruitment approach was adjusted to accommodate recruitment outside of the context of schools due to the COVID-19 pandemic. Most school systems transitioned to remote learning in Massachusetts and the largest school district working with us ceased all school-based research for the remainder of the school year. As a result, all study activities were modified for remote delivery and travel was no longer required for the study team; therefore, the scope was broadened to include schools in the Eastern region of the Massachusetts, and later, across the state. Various strategies were used, including working with an outside recruitment agency to recruit families online, applying snowball recruitment methods by asking participating families to share the opportunity with their contacts, and engaging with community-based organizations currently working with parents to help disseminate the opportunity.

### Study timeline

**Fig 1:**
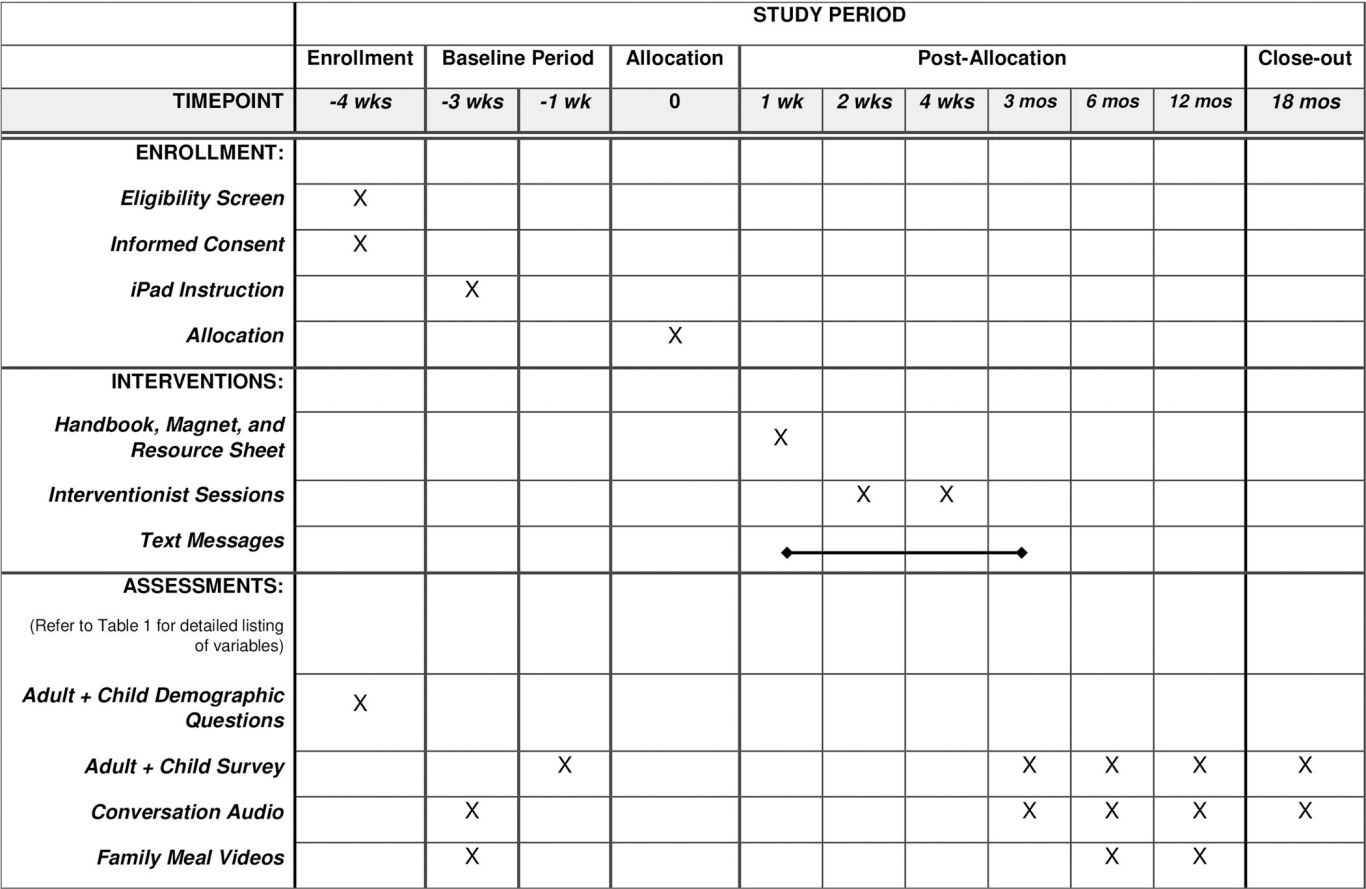
Schedule of enrollment, intervention and assessments.

A complete description of the study timeline can be found in Fig 1, an adaptation of the SPIRIT figure.

### Screening and eligibility

Interested parents are screened using a brief online survey that is administered by a research assistant over the phone. To be eligible, parents must be a custodial parent living with their child at least 50% of the time and child participants must be a student in 5^th^, 6^th^, or 7^th^ grade and enrolled in school within Massachusetts. During summer-month recruitment (June through August), students entering 5^th^ grade and those who just completed 7^th^ grade are eligible. Parents and children with a developmental disability or limited proficiency in English or Spanish that would interfere with their independent completion of surveys and study activities are excluded. Only one parent-child dyad per family is allowed. If the parent is eligible and interested in continuing with participation, the research assistant schedules the consent and baseline meeting.

### Consent and baseline meeting

Baseline meetings are conducted in person at the participant’s home or another place of their choosing, and the participant receives all study materials. Due to the COVID-19 pandemic, after March 2020, meetings were conducted virtually via web-conference and all study materials were sent via carrier mail. Remote visits were implemented as a permanent option for the remainder of the study. An electronic copy of the consent and assent forms are emailed to the dyad prior to the visit. During the meeting for both in-person and remote options, informed consent/assent are obtained from the participants before beginning data collection. Demographic data from both parent and child are collected via an online survey. Research assistants then review the procedures for using a study-provided iPad to record and upload conversations and family meals that they are to complete and upload independently during the baseline and follow-up visits. Dyads are then asked to complete all baseline study activities.

Participants complete baseline data collection over a period of four weeks immediately following the consent and baseline meeting to give participants a sense of the time commitment required by the study. Participants are randomized after all baseline data are completed. Dyads that do not complete baseline activities are not randomized.

### Randomization

Every week, parent-child dyads are randomized to receive the experimental or control condition using stratified block urn randomization, programmed in SAS, based on the school in which the child participant is enrolled, child grade, and child-reported gender. The project manager assigns the interventionist and sends the participants their respective condition materials. Only the project manager, Principal Investigator, and one Co-Investigator (the biostatistician on the study who conducts randomization) have access to the assigned condition, which is stored separately from the participant’s personal data and any other study-related data.

### Follow-up time periods

Data are collected at four follow-up time points, 3, 6, 12 and 18 months post-randomization. Timepoints were chosen to assess the immediate (3-month), short-term (6-month) and long-term (12- and 18-month) effects of the intervention and to improve the chance of observing differences in substance use initiation. Each timepoint has a four-week window for participants to complete all study activities.

### Participant retention

Participants receive automatically generated emails and personalized text message reminders during each follow-up period until all data are submitted. Participants are encouraged to reach out with questions and to troubleshoot issues with data submissions. To further support participant engagement and retention, the study team sends personalized communication throughout the study period (i.e., greeting cards every three months, text messages at 9- and 15-month follow-up time periods, and a frequently asked questions website). Retention is monitored regularly, and stakeholder and key informant interviews help inform best practices for retaining recruited participants and schools in real time.

### SUPPER experimental condition

Participants randomized to the experimental condition receive a brief substance use prevention program with two components: live and home-based. After randomization, the parent participant is mailed a packet with the home-based components including a: handbook, magnet, local resources handout, and link to the study website. In addition, the parent receives two text messages per week for thirteen weeks with tips and reminders from the handbook, starting immediately following randomization. The live component includes two meetings with a study interventionist, which is either in person or via videoconference.

#### Home-based components

*Handbook*: *“Keeping Your Child Safe from Alcohol and Drugs*.*”* The handbook for the prevention program condition is gender-specific, written at an eighth-grade reading level, and focuses on five domains: 1) statistics of adolescent substance use; 2) importance of and strategies to effectively communicate with your child; 3) eating family meals together and alternatives if family meals are not possible; 4) suggested content for high quality substance use conversations; and 5) the role of substance-using peers with respect to adolescent substance use and effective peer management strategies. Gender-specific statistics and reasons why teens use substances are also included. Parent participants are able to choose the handbook that aligns with their child’s gender and are able to get both (which have substantial overlap) if they prefer.

*Prevention Program Magnet*. The magnet serves as a visual cue to remind families to eat meals with their child as often as they can, and that eating and talking together can help kids stay alcohol- and drug- free.

*Text Messages*. Two automated text messages are delivered weekly for 13 weeks with reminders and tips that reinforce the information covered in the handbook. Participants that do not have a phone that can receive text messages are provided with one.

*Study Website*. The website provides information about substance use, parent-child communication, and links to additional resources not included in the handbook.

*Local Resources Handout*. The handout includes mental health resources and local organizations that provide substance use and recovery services, family health centers and services, and substance abuse and mental health treatment referral services.

#### Live component

*Prevention Program Sessions*. The parent is asked to participate in two live sessions, an approximately one-hour in-person/video session (Session 1) and a 30-minute follow-up phone call (Session 2) with a study interventionist. Session 1 takes place approximately two weeks after receiving the handbook and Session 2 approximately two weeks after Session 1. The purpose of the sessions is to increase parent knowledge in the material covered in the handbook and self-efficacy for having conversations about substance use with their child. Interventionists are trained by the study team using standardized training in a motivational interviewing style and method, general therapeutic techniques, and how to administer an action plan and communicate key points from the handbook. The action plan serves as a guide for the participant to plan their substance use conversations with their child, encouraging them to consider possible barriers, people who could support them, when to have the conversations, and specifically what they would like to say. During Session 1, the interventionist asks the participant to complete a brief questionnaire to assess their knowledge of different key points from the handbook, reviews the handbook’s key points, and helps the participant set a realistic goal and practice for having a conversation about substance use with their child using the action plan as a guide. The interventionist follows up during Session 2 about progress on the action goals, reviews the handbook key points and answers any questions.

### Attention-control (comparison) condition

Parent-child dyads randomized to the comparison condition receive the same dose with respect to contact time and materials as the experimental condition, but with a focus on nutrition, physical activity, and weight stigma. The handbook “Helping Your Child: Tips for Parents” is adapted from the *Healthy Eating and Physical Activity Across your Lifespan Series* from the National Institute of Diabetes and Digestive and Kidney Diseases [29]. Parents receive an additional handout, which delivers guidance for parents on best practices for talking about weight with children in a non-stigmatizing way. The handout was developed using evidence-based practices informed by content experts and a literature review. Text messages focus on key points from the handbook and handout. Sessions with the interventionist use a similar action plan layout to guide parents in setting goals to improve child eating and exercise habits and reduce the occurrence of negative or critical comments about weight from family members. After study completion, control participants can obtain the prevention program materials if desired.

### Data collection

Participants are asked to submit data through two direct observation methods (i.e., conversation recordings and meal recordings), as well as quantitative surveys throughout the baseline and follow-up time points. iPads are provided to the participants during baseline (or mailed prior to baseline when baseline is virtual) to complete all study-related activities.

#### Parent-child conversation recording

Dyads audio record 20-minute prompted conversations at every time point (baseline, 3-, 6-, 12- and 18-month). The prompts, modeled after the Family Assessment Task (FAsTask) where parents and adolescents have a conversation about substance use and related behaviors [30,31], were developed by the study team and key informant interviews. The same prompts are used at each time point. The prompts are designed to facilitate a discussion between parents and children on substance use (10 minutes) and eating habits, exercise, and talking about weight (10 minutes).

#### Family meal video recordings

Modeled after procedures introduced in the “Family Meals, LIVE!” study [32], dyads are asked to video record four family meals (three weekday and one weekend) at three timepoints (baseline, 6- and 12-month). Participants are instructed to first record a weekday meal, which serves for participant acclimation to being recorded and is not intended to be used in the analysis. A family meal is defined as when the parent and child participants are together and at least one is eating. Meals are recorded using the front facing camera such that the parent and child participant can be clearly seen and heard.

#### Surveys

Parent and child participants both complete approximately 60-minute online surveys at all timepoints (baseline, 3-, 6-, 12- and 18-months). Surveys are administered via Research Electronic Data Capture (REDCap), a secure online data collection system [33,34]. The parent participant is sent two emails: one containing the link to the parent survey and one containing the link to the child survey. Paper surveys were used in cases when the online surveys were not available then entered into REDCap using double data entry to ensure accuracy. The parent and child are asked to not share their survey responses. Built-in validations are included in the online survey to increase internal validity of the survey. Missing data are regularly monitored, and study staff contact participants when missing data are observed.

### Data measures

The data collection tools described above are used to assess changes in study outcomes from baseline and to assess risk and protective factors across multiple domains as aligned with the study’s Aims. All measures are assessed for change from baseline. Details of all assessments are organized below in Table 1.

**Table 1:** Study measures timeline.

Outcome	Source	Instrument/Scale	Timeline Parent					Timeline Child				
			B	3M	6M	12M	18M	B	3M	6M	12M	18M
**Aim 1:**												
Frequency of parent-child conversations about substance use	Survey	Single item from Miller, et al. 1998, repeated for each substance* [35]	X	X	X	X	X	X	X	X	X	X
Quality of targeted parent-child communication about alcohol and other drugs	Survey	Adapted Targeted Parent-Child Communication about Alcohol scale from Miller, et al. 2010 [36]	X	X	X	X	X	X	X	X	X	X
Quality of substance use conversations	Audio	Tool adapted from FasTask and codebook developed by authors [31]	X	X	X	X	X	X	X	X	X	X
Quality of mealtime interactions	Video	Used 13 sub-scales from the Iowa Family Interaction Rating Scales (IFIRS) [37]	X		X	X		X		X	X	
**Aim 2:**												
Child substance use attitudes and expectancies Alcohol Cigarettes Marijuana Vaping / e-cigarettes Other drugs	Survey	Short Alcohol Expectancies Scale [38]						X	X	X	X	X
		Positive and Negative Expectancies of Smoking [39]										
		Marijuana Effect Expectancies Questionnaire-Brief [40,41]										
		E-cigarette Expectations [42]										
		Adapted Marijuana Effect Expectancies Questionnaire-Brief [40]										
Child substance use intentions	Survey	Two items adapted from the Youth Alcohol and Drug Survey, repeated for each substance* [43]						X	X	X	X	X
Child willingness to use substances	Survey	Behavioral willingness items from Gibbons, et al. 1998, repeated for each substance* [44]						X	X	X	X	X
Child affiliation with peers who use substances	Survey	Single item from Monitoring the Future Survey, repeated for each substance* [45]						X	X	X	X	X
**Aim 3:**												
Child initiation of substance use	Survey	Two items adapted from Drug Use Questionnaire, repeated for each substance* [46]						X	X	X	X	X
Child incidence of substance use	Survey	Single item from Drug Use Questionnaire, repeated for each substance* [46]						X	X	X	X	X

*Repeated for each substance means that the same item was used to ask about each of the following substances specifically: alcohol, cigarettes, marijuana, e-cigarettes/vaping and other drugs.

#### Sociodemographic variables

Parent items include: date of birth, gender, race, ethnicity, marital status, acculturation [47], education, employment status, work schedule, household structure, and household income. Child items include: grade, date of birth, gender, race, ethnicity, household structure, and acculturation [47].

#### Frequency of parent-child conversations about substance use

Parent-and child-reported frequency of parent-child conversations are measured using an item adapted from a measure for parent-child communication about sex [35]: “During [time frame], how many times have you and your child talked about [substance]?.” The same item assesses frequency for each substance of interest (smoking cigarettes, drinking alcohol, using e-cigarettes or vaping, using marijuana, using other drugs) using the respective time frame between follow ups (i.e., ever, past 3 months, or past 6 months). These items have a five-point Likert scale from “None” to “A lot”.

#### Targeted parent-child communication about substances

This scale is an adapted version of the Targeted Parent-Child Communication about Alcohol Scale, a measure with demonstrated reliability and validity [36]. Several dimensions are assessed including parental warnings about the dangers of drugs, advice for how to address drug situations such as offers or peer pressure, and articulation of rules and sanctions around drugs. Ten items are asked of the parent and child at each time point. Items are assessed on a six-point Likert scale from “Strongly agree” to “Strongly disagree”.

#### Quality of mealtime interactions

To measure the dyadic and family-level interpersonal dynamics and behaviors, thirteen scales from the Iowa Family Interaction Rating Scales (IFIRS) coding system [37] are used: Silence/Pause, Relationship Quality, Group Enjoyment, Hostility, Lecture Moralize, Warmth Support, Listener Responsiveness, Communication, Neglecting Distancing, Indulgent/Permissive, Parental Influence, Positive Reinforcement and Intrusive. Behaviors are assessed and scored on a 9-point Likert scale from “Not at all characteristic” to “Mainly characteristic”.

#### Quality of conversations about substance use

To qualitatively measure the change in the quality of conversations about substance use, a self-developed SUPPER Family Conversation Tool (SUPPER-FCT) is used. The tool, modeled after FAsTask, an assessment tool used in prevention and intervention studies on youth substances [30,31], measures four constructs: 1) parental expectations on substance use; 2) parental targeted messaging about the effects of substance use; 3) quality of parent-child communication and style (e.g. permissive vs. restrictive); and 4) discussion of strategies to handle situations where the child is exposed to substances and parental encouragement for the child to come to the parent with substance use-related questions and discussions. Constructs are scored on a 9-point Likert scale with higher scores representative of higher quality conversations.

#### Child substance use attitudes and expectancies about substance use

Positive and negative expectancies about the affective, cognitive, and behavioral effects of substances are assessed at each timepoint. An adapted version of the Short Alcohol Expectancy Scale is used to measure alcohol expectancies. Cigarette expectancies are measured using a 12-item tool with high reliability and validity [39]. A ten-item measure is used to measure e-cigarette expectancies [42]. Three subscales, relaxation/tension reduction, cognitive/behavioral impairment and global negative effects, from the Marijuana Effect Expectancy Questionnaire (MEEQ) are used to measure marijuana expectancies. Each expectancy is scored on a six-point Likert scale from “Strongly disagree” to “Strongly agree.”

#### Child substance use intentions

The same two items are used to assess the child’s intention to use each substance: cigarettes, e-cigarettes, alcohol, marijuana and other drugs. Items are from the Youth Alcohol and Drug Survey and have high estimates of validity and reliability [43,48]. Items ask if the child plans to use the substance during the next 30 days and within the time period until they take their next survey (i.e. either 3- or 6-months). A 4-point Likert scale (“No”, “Probably No”, “Probably Yes” and “Yes”) is used with a higher score indicating a greater intention to use the substance.

#### Child willingness to use substances

Child-reported willingness to use cigarettes, e-cigarettes, alcohol, marijuana, and other drugs is captured using three items adapted from intention and willingness measures for tobacco and amphetamines [44]. The child is prompted to imagine they are in a situation where they are offered a substance by their friend and asked how likely they would be to take and try the substance, tell their friends “no”, and leave the situation. Items are scored using a six-point Likert scale from “Not at all” to “Very likely.” A higher mean score indicates greater willingness to try the substance.

#### Child affiliation with peers who use substances

Child-reported affiliation with peers who use substances is captured using items from the Monitoring the Future study [45]. Teens are asked how many of their friends use substances occasionally and regularly, and how their close friends would feel about their substance use. Items are scored on a five-point Likert scale from “None” to “All.” A higher score indicates that a greater number of peers use substances. Pilot trial data generated Cronbach’s coefficients between 0.80 and 0.85 for affiliation with substance using peers and 0.75 and 0.83 for peer tolerance of substance use [49].

#### Child initiation of substance use

Child-reported initiation of using use cigarettes, e-cigarettes, alcohol, marijuana, and other drugs is captured using items adapted from the Drug Use Questionnaire [46]. Each item asks if the child has ever tried the substance using dichotomous Yes/No response options. Child participants who reported having ever used a substance are subsequently asked to report the date (i.e., month, day, and year) they first tried or used the substance.

#### Incidence of child substance use

Child-reported incidence of using cigarettes, e-cigarettes, alcohol, marijuana, and other drugs is captured using a single item from the Drug Use Questionnaire measured on a six-point Likert scale from “Never” to “Several times a day” [46].

Potential mediators or effect modifiers of the intervention’s effect as well as potential independent factors will be assessed during the trial using a series of measures including: general quality of parent-child communication [50], child conversations with other people about substances [35], parental self-efficacy for having conversations with their child about substances (developed by authors), child comfort level talking about drugs and alcohol [35], affiliation with substance-using peers [51], peer norms about substance use [51], parental and other household member substance use [52,53], parental problems with substance use [54], parental report of other child or adult substance use [46], parental attitudes towards drug use [55], self-esteem [56], mental health [57], frequency of family meals [28], family dinner index [28], parenting styles [58], child adverse experiences [59], parent-reported child resiliency [60], perceived neighborhood disorder [61], and social desirability [62,63].

### Intervention fidelity

To ensure treatment fidelity, we adopted recommended practices by the National Institutes of Health Behavior Change Consortium Treatment Fidelity Workgroup [64,65] including those for training interventionists, delivery of treatment, and receipt of treatment.

Interventionists for the intervention arm undergo standardized training in session content and motivational interview style to ensure sessions are delivered consistently and as designed in the protocol. All sessions are audio-recorded for fidelity assessment. The first ten sessions of newly onboarded interventionists are assessed by supervisors. After observing the session, the interventionist and supervisor meet for feedback, support and norming. Once an acceptable level of adherence is met, 20% of sessions continue to be monitored. Refresher trainings are conducted if interventionists are nonadherent.

To ensure fidelity delivery and receipt of the intervention, five process measures (dose, adherence, quality of delivery, participant responsiveness and cross contamination) were adapted and are monitored at regular intervals throughout the study [66]. Process measure data are extracted from self-administered and supervisor intervention surveys and participant self-reported surveys.

To minimize cross-contamination risk, participants are asked not to share information about the content of the intervention with anyone outside their household. Cross-contamination is also measured by asking a question in the parent and child survey at the 3- and 18- month assessment point.

### Blinding

Due to the nature of the study, the PI and statistician Co-I, interventionists, and the Project Manager, who are responsible for sending participants program materials, contacting participants, and randomization, are unblinded to group allocation. All study staff involved in data collection and coding are blinded. Blinded study team members only see anonymized Participant Identification Numbers (PID) when coding the parent-child conversation and family meal recordings.

### Compensation

Dyads are compensated with a $60 gift card for completing the baseline, 3- and 6-month surveys, and a $80 gift card for the 12- and 18-month follow-up surveys to encourage retention. Participant dyads are also eligible to keep the iPad as compensation at the end of the study if they remain in the study and complete the 18-month assessment.

### Harm

This study was approved by Tufts University Social, Behavioral and Educational Research IRB (Study # 1805018; original approval: June 12, 2018; most recent approval: June 29, 2021), in addition to gaining research approval through the Boston Public Schools Office of Data Accountability (RA-72 SY1819). All study materials and protocols have been ethically reviewed, both in English and Spanish where appropriate. All substantial amendments will be reviewed by the Tufts Health and Sciences Institutional Review Board.

It is not anticipated that any serious adverse events will occur during this research. Any adverse events observed and/or reported during assessments or sessions are reported immediately to the PI and the Tufts Health and Sciences Institutional Review Board (IRB). In all cases, necessary action is taken, including reporting to authorities, to prevent serious harm to participants, children, or others. Any recommendations to change the protocol as a result of an adverse event is to be included in the study’s standard operating procedures and implemented immediately.

### Data management and storage

Study data are managed using standard procedures approved by the IRB. Study data are collected and entered by the Project Manager and RAs. To protect participant privacy, data are coded using a PID. The link between identifiable information and PIDs are password-protected and kept separately from the data; the link document is only accessible to the PI and Project Manager. Data are accessible only to the PI, Project Manager, RAs, and biostatistician.

Electronic survey data and electronic study documents (e.g., electronic copies of signed consent and assent forms, audio and video recordings) are stored on Tufts Box, a Health Insurance Portability and Accountability Act (HIPAA)-compliant cloud storage service, and in the case of forms and surveys on the Tufts REDCap server [33,34]. All data are backed up regularly on a secure, password-protected external hard drive and stored in a locked cabinet in a locked room. Hard copy data, such as paper surveys, are kept confidential and stored in locked cabinets in a locked office. Data will be kept for five years following close of the study.

### Data monitoring

Oversight of internal monitoring of the participants’ safety and trial conduct is conducted by the PI. Weekly meetings with the PI, co-investigators, and staff are used to evaluate the progress of the trial, review data quality, recruitment, and study retention, and examine factors that may affect outcomes. The rates of adverse events are also reviewed to determine any changes in participant risk. A brief report is generated quarterly for the study record and forwarded to the IRB. A summary of this information regarding adverse events is provided in the annual report to National Institutes of Health.

### Sample size

The trial is powered on the outcome of parent-child communication about substance use, which was most responsive to intervention in the pilot trial. It is also most relevant for reducing the risk of substance use initiation. Results from the pilot trial showed that among parents who received the intervention, one-half (55% compared to 10% in the control condition) talked “a lot” to their child over the 6-month study period about alcohol, 35% (compared to 15%) talked “a lot” about marijuana, and 30% (compared to 10%) talked “a lot” about other drugs. A total of 400 parent-child dyads yields 80% power to detect one or more differences between experimental and comparison conditions, of 45% in talking about alcohol, 20% about marijuana, and 20% about other drugs, using a two-sided Bonferroni-corrected 1.5% level of significance (to account for testing 3 substances). With regard to the video assessment of quality of parent-child conversations about substance use, using an average standard deviation of 1.0 for the FAsTask from a recently published pilot trial that assessed an intervention for substance use in a sample of young adolescents [67], a sample size of 400 yields 80% power to detect a mean difference of 0.4 or greater (effect size = 0.4) between conditions at a two-sided 5% significance level. This is a meaningful difference as the ratings for this task ranges from 1 to 9. Accounting for a 20% dropout rate, the size of trial is 500 in total, evenly spread across the three grades, for a total of 400 dyad completers.

### Planned analyses

The scientific rigor of this trial will be ensured by conducting ongoing data integrity assessments, missing data detection and correction procedures, and proposed data analysis strategies. All forms will be checked for missing data and extreme values prior to data entry. Attrition effects will be evaluated by testing whether systematic differences exist between participants who complete the trial follow-ups versus those who drop out. Differences between the two conditions in demographic composition and all measures collected at baseline will be assessed at regular intervals throughout the duration of the trial. Group differences in these variables at trial’s end will be accounted for in subsequent analyses, using baseline scores as covariates. All analyses will subscribe to the intention-to-treat principle, whereby all participants will be included in the analysis in the group to which they were randomized. Participants who are lost to follow-up or drop out can affect the validity of the group comparisons if: 1) the outcome of interest is related to being lost or dropped, and 2) it is differential between groups. The missing data mechanism will be formally tested [68,69] to determine whether it is Missing Completely at Random (MCAR) or Missing at Random (MAR). For completeness in assessing the effect of missingness, two additional analyses will be conducted: 1) a ‘per-protocol’ analysis among participants who complete all follow-up surveys; and 2) multiple imputation of five data sets using a discriminant function for binary outcomes and Markov chain Monte Carlo for continuous outcomes, assuming the missing data mechanism is MAR.

#### Aim 1: To determine the efficacy of the brief intervention on parent outcomes

The proportion of parents who more frequently communicate to their child about substance use will be compared between conditions across the follow-up periods using a repeated measures log-binomial regression analysis (via generalized estimating equations), with an appropriate covariance structure and adjusting for baseline scores. Covariates will be added to the modeled analyses if differences among baseline characteristics are observed. A test for interaction between conditions and follow-up points using the above models will assess whether there is a differential effect of the experimental condition across time. To assess quality of mealtime interactions, the videos will be scored according to the IFIRS and then analyzed using repeated measures analysis of covariance (i.e., a linear mixed model) to compare the two conditions across the follow-ups, using an appropriate covariance structure and adjusting for baseline scores. A similar analysis will be used for the summed scores from the parent-child conversations about substance use.

#### Aim 2: To examine the effects of the prevention program on child substance use beliefs, intentions, and willingness, and affiliation with substance-using peers

The analysis will compare the proportion of children with intentions and willingness to use substances, negative substance use attitudes and expectancies, and substance-using peers between conditions across the follow-up periods using a repeated measures log-binomial regression (via generalized estimating equations), with an appropriate covariance structure and adjusting for baseline scores.

#### Secondary aim: To examine the effects of the prevention program on substance use initiation

The analysis will assess whether children of parents in the experimental condition delay the initiation of substance use longer than the comparison condition by comparing the incidence of initiation using a discrete-time survival analysis via a complementary log-log regression model, as the date of initiation between follow-up time points may not be known exactly.

#### Exploratory analyses by race and ethnicity

Because it is known that subgroup analyses have less statistical power than the primary analysis, an exploratory analysis will be conducted to determine whether the efficacy of the experimental condition varies by race and ethnicity, to inform future adaptations.

## Discussion

Adolescent substance use is a national problem and while decreasing with some substances, continues to persist and escalate in others [5,6]. The COVID-19 pandemic has added further urgency to addressing this issue as adolescents may turn to substances to cope or self-medicate during this difficult time [10–12]. Currently there is lack of family-based, universal substance use prevention programming to reduce and/or delay substance use initiation among youth that reduce participant burden, are easily disseminated, and sustainable. This protocol outlines the methodology to test the effectiveness of a brief, multi-pronged parent-focused intervention promoting family meals that uses a tailored approach to improve parents’ self-efficacy in communicating with their child about substances. The strengths of this study are: first, rather than solely focusing on quantity of family meals and conversations about substance use, we code qualitative data to measure quality of these interactions; second, we assess these outcomes through observation rather than relying exclusively on self-report; and third, a large, diverse cohort of parent-child dyads to examine study outcomes.

Due to the COVID-19 pandemic, many of our procedures changed which presented both challenges and benefits. We were able to quickly pivot our recruitment, baseline meeting, and interventionist session procedures to be virtual. Families appeared to transition well to virtual activities, likely due to many of their other work and school activities being moved to the same web-based platforms. Interventionists reported being able to successfully build rapport with parents in virtual sessions.

Our strategy for recruiting specifically within schools in the Greater Boston area was challenged because the district paused research activities. However, many schools continued to promote the program. Being completely remote allowed recruitment to be extended to families throughout the state, thereby opening the sampling pool and allowing the team to offer more scheduling options to families and to schedule more meetings in a day than previously. An online recruitment agency was employed to disseminate the opportunity state-wide to supplement lulls in school-based recruitment. One limitation to this online recruitment platform was that despite making the recruitment materials and screener available in Spanish, the platform itself is English-only, requiring the Spanish speaker to know enough English to make an account.

This study will provide significant contributions to the limited literature on the promotion of family meals and open communication [22] as an innovative approach to substance use prevention. Further, because of the brief nature of the intervention and low burden on the participant and program staff, if found effective, it could be easily adopted by schools for implementation.

## Supporting information

S1 Checklist SPIRIT checklist. (DOC)

S1 File (PDF)

S2 File (PDF)

S3 File (PDF)

S4 File (PDF)

S5 File (DOCX)

S6 File (DOCX)

## References

[1] Flory K, Lynam D, Milich R, Leukefeld C, Clayton R. Early adolescent through young adult alcohol and marijuana use trajectories: Early predictors, young adult outcomes, and predictive utility. 2004;16(01). 10.1017/s0954579404044475 15115071

[2] McGue M, Iacono WG, Legrand LN, Malone S, Elkins I. Origins and Consequences of Age at First Drink. I. Associations With Substance-Use Disorders, Disinhibitory Behavior and Psychopathology, and P3 Amplitude. 2001;25(8):1156–65. 11505047

[3] Lipari RN, Park-Lee E. Key substance use and mental health indicators in the United States: results from the 2018 National Survey on Drug Use and Health. Substance Abuse and Mental Health Services Administration Published. 2019. 29431966

[4] Volkow ND, Han B, Einstein EB, Compton WM. Prevalence of substance use disorders by time since first substance use among young people in the US. JAMA pediatrics. 2021. doi: 10.1001/jamapediatrics.2020.6981 33779715 PMC8008418

[5] Centers for Disease C, Prevention. Trends in the prevalence of alcohol use national YRBS: 1991–2019. Atlanta, GA: Centers for Disease Control and Prevention. 2020.

[6] Centers for Disease C, Prevention. Trends in the Prevalence of Marijuana, Cocaine, and Other Illegal Drug Use National YRBS: 1991–2019. 2020.

[7] Prevention CfDCa. Youth Risk Behavior Survey 2019 [Available from: www.cdc.gov/yrbss.

[8] Cullen KA, Gentzke AS, Sawdey MD, Chang JT, Anic GM, Wang TW, et al. e-Cigarette Use Among Youth in the United States, 2019. JAMA. 2019;322(21):2095–103. doi: 10.1001/jama.2019.18387 31688912 PMC6865299

[9] Wang TW, Gentzke A, Sharapova S, Cullen KA, Ambrose BK, Jamal A. Tobacco product use among middle and high school students—United States, 2011–2017. Morbidity and Mortality Weekly Report. 2018;67(22):629. doi: 10.15585/mmwr.mm6722a3 29879097 PMC5991815

[10] de Figueiredo CS, Sandre PC, Portugal LCL, Mázala-de-Oliveira T, da Silva Chagas L, Raony Í, et al. COVID-19 pandemic impact on children and adolescents’ mental health: biological, environmental, and social factors. Progress in Neuro-Psychopharmacology and Biological Psychiatry. 2021;106:110171. doi: 10.1016/j.pnpbp.2020.110171 33186638 PMC7657035

[11] Bhatia G, Chatterjee B, Dhawan A. Adolescents, Drugs, and COVID-19: Special Challenges During the Pandemic. SAGE Publications Sage India: New Delhi, India; 2021. 10.1177/0253717621988998 PMC8313445 34376882

[12] Ingoglia C. COVID-19 and Youth Substance Use: We Need More than Good Intentions. The Journal of Behavioral Health Services & Research. 2021;48(1):1–3. doi: 10.1007/s11414-020-09739-9 33230653 PMC7682947

[13] Dumas TM, Ellis W, Litt DM. What Does Adolescent Substance Use Look Like During the COVID-19 Pandemic? Examining Changes in Frequency, Social Contexts, and Pandemic-Related Predictors. J Adolesc Health. 2020;67(3):354–61. doi: 10.1016/j.jadohealth.2020.06.018 32693983 PMC7368647

[14] Cook RLMDMPH Comer DM, Wiesenfeld HCMDCM Chang C-CHP, Tarter RP Lave JRP, et al. Alcohol and Drug Use and Related Disorders: An Underrecognized Health Issue Among Adolescents and Young Adults Attending Sexually Transmitted Disease Clinics. Sexually Transmitted Diseases. 2006;33(9):565–70. doi: 10.1097/01.olq.0000206422.40319.54 16572042

[15] Dembo R, Williams L, Schmeidler J, Wish ED, Getreu A, Berry E. Juvenile Crime and Drug Abuse. Journal of Addictive Diseases. 1992;11(2):5–31. 10.1300/j069v11n02_02 1811760

[16] Abuse NIoD. National institute on drug abuse 2016–2020 strategic plan. 2015.

[17] Skeer MR, McCormick MC, Normand S-LT, Mimiaga MJ, Buka SL, Gilman SE. Gender differences in the association between family conflict and adolescent substance use disorders. Journal of Adolescent Health. 2011;49(2):187–92. doi: 10.1016/j.jadohealth.2010.12.003 21783052 PMC3143378

[18] Spoth R, Randall GK, Shin C, Redmond C. Randomized study of combined universal family and school preventive interventions: patterns of long-term effects on initiation, regular use, and weekly drunkenness. Psychology of addictive behaviors. 2005;19(4):372. doi: 10.1037/0893-164X.19.4.372 16366809 PMC2409287

[19] Vakalahi HF. Adolescent substance use and family-based risk and protective factors: A literature review. Journal of drug education. 2001;31(1):29–46. doi: 10.2190/QP75-P9AR-NUVJ-FJCB 11338964

[20] O’Connell ME, Boat T, Warner KE. Preventing mental, emotional, and behavioral disorders among young people: Progress and possibilities: Washington, DC: National Academies Press; 2009. 20662125

[21] Kumpfer KL, Alvarado R, Whiteside HO. Family-Based Interventions for Substance Use and Misuse Prevention. 2003;38(11–13):1759–87. 10.1081/ja-120024240 14582577

[22] Velleman RD, Templeton LJ, Copello AG. The role of the family in preventing and intervening with substance use and misuse: a comprehensive review of family interventions, with a focus on young people. Drug and alcohol review. 2005;24(2):93–109. doi: 10.1080/09595230500167478 16076580

[23] Eisenberg ME, Neumark-Sztainer D, Fulkerson JA, Story M. Family Meals and Substance Use: Is There a Long-Term Protective Association? 2008;43(2):151–6. 10.1016/j.jadohealth.2008.01.019 18639788

[24] Eisenberg ME, Olson RE, Neumark-Sztainer D, Story M, Bearinger LH. Correlations Between Family Meals and Psychosocial Well-being Among Adolescents. 2004;158(8):792. 10.1001/archpedi.158.8.792 15289253

[25] Sen B. The relationship between frequency of family dinner and adolescent problem behaviors after adjusting for other family characteristics. Journal of adolescence. 2010;33(1):187–96. doi: 10.1016/j.adolescence.2009.03.011 19476994

[26] Bien TH, Miller WR, Tonigan JS. Brief interventions for alcohol problems: a review. Addiction. 1993;88(3):315–36. doi: 10.1111/j.1360-0443.1993.tb00820.x 8461850

[27] Monti PM, Colby SM, Barnett NP, Spirito A, Rohsenow DJ, Myers M, et al. Brief Intervention for Harm Reduction With Alcohol-Positive Older Adolescents in a Hospital Emergency Department. Journal of Consulting and Clinical Psychology. 1999;67(6):989–94. doi: 10.1037//0022-006x.67.6.989 10596521

[28] Skeer MR, Yantsides KE, Eliasziw M, Carlton-Smith AR, Tracy MR, Spirito A. Testing a brief substance misuse preventive intervention for parents of pre-adolescents: feasibility, acceptability, preliminary efficacy. Journal of child and family studies. 2016;25(12):3739–48. doi: 10.1007/s10826-016-0525-3 28163563 PMC5286462

[29] Diseases NIoDaDaK. Helping Your Child: Tips for Parents.

[30] Dishion TJ, Kavanagh K. Intervening in adolescent problem behavior: A family-centered approach. New York, NY, US: Guilford Press; 2003. xi, 243–xi, p.

[31] Spirito A, Sindelar-Manning H, Colby SM, Barnett NP, Lewander W, Rohsenow DJ, et al. Individual and family motivational interventions for alcohol-positive adolescents treated in an emergency department: results of a randomized clinical trial. Archives of Pediatrics & Adolescent Medicine. 2011;165(3):269–74. 21383276 10.1001/archpediatrics.2010.296 PMC3690344

[32] Berge JM, Rowley S, Trofholz A, Hanson C, Rueter M, Maclehose RF, et al. Childhood Obesity and Interpersonal Dynamics During Family Meals. PEDIATRICS. 2014;134(5):923–32. doi: 10.1542/peds.2014-1936 25311603 PMC4210801

[33] Harris PA, Taylor R, Minor BL, Elliott V, Fernandez M, O’Neal L, et al. The REDCap consortium: Building an international community of software platform partners. Journal of Biomedical Informatics. 2019;95:103208. doi: 10.1016/j.jbi.2019.103208 31078660 PMC7254481

[34] Harris PA, Taylor R, Thielke R, Payne J, Gonzalez N, Conde JG. Research electronic data capture (REDCap)—A metadata-driven methodology and workflow process for providing translational research informatics support. Journal of Biomedical Informatics. 2009;42(2):377–81. doi: 10.1016/j.jbi.2008.08.010 18929686 PMC2700030

[35] Miller KS, Kotchick BA, Dorsey S, Forehand R, Ham AY. Family Communication About Sex: What are Parents Saying and Are Their Adolescents Listening? 1998;30(5):218. 9782044

[36] Miller-Day M, Kam JA. More Than Just Openness: Developing and Validating a Measure of Targeted Parent–Child Communication About Alcohol. Health Communication. 2010;25(4):293–302. doi: 10.1080/10410231003698952 20512711 PMC2879702

[37] Melby JN, Conger RD. The Iowa Family Interaction Rating Scales: Instrument summary. In Kerig P. K. & Lindahl K. M. (Eds.), Family observational coding systems: Resources for systemic research (pp. 33–58). Lawrence Erlbaum Associates Publishers. 2001.

[38] Aas H. Adaptation of the Alcohol Expectancy Questionnaire (AEQ‐A): A short version for use among 13‐year‐olds in Norway. Scandinavian Journal of Psychology. 1993;34(2):107–18. doi: 10.1111/j.1467-9450.1993.tb01106.x 8322045

[39] Dalton MA, Sargent JD, Beach ML, Bernhardt AM, Stevens M. Positive and negative outcome expectations of smoking: implications for prevention. Preventive medicine. 1999;29(6):460–5. doi: 10.1006/pmed.1999.0582 10600426

[40] Aarons GA, Brown SA, Stice E, Coe MT. Psychometric evaluation of the marijuana and stimulant effect expectancy questionnaires for adolescents. Addictive behaviors. 2001;26(2):219–36. doi: 10.1016/s0306-4603(00)00103-9 11316378

[41] Hayaki J, Hagerty CE, Herman DS, de Dios MA, Anderson BJ, Stein MD. Expectancies and marijuana use frequency and severity among young females. Addictive Behaviors. 2010;35(11):995–1000. doi: 10.1016/j.addbeh.2010.06.017 20621423 PMC2919625

[42] Noar SM, Rohde JA, Horvitz C, Lazard AJ, Cornacchione Ross J, Sutfin EL. Adolescents’ receptivity to E-cigarette harms messages delivered using text messaging. Addict Behav. 2019;91:201–7. doi: 10.1016/j.addbeh.2018.05.025 29960716 PMC6275144

[43] Werch C. The Youth Alcohol and Drug Survey. Jacksonville, FL: University of North Florida. 1996.

[44] Gibbons FX, Gerrard M, Blanton H, Russell DW. Reasoned action and social reaction: willingness and intention as independent predictors of health risk. Journal of personality and social psychology. 1998;74(5):1164. doi: 10.1037//0022-3514.74.5.1164 9599437

[45] Johnston LD, O’Malley PM, Bachman JG, Schulenberg JE. Monitoring the Future national results on adolescent drug use: Overview of key findings, 2012. 2013.

[46] Spirito A. Reliability data for the drug use questionnaire. Providence, RI: Brown University. 2001.

[47] Cruz TH, Marshall SW, Bowling JM, Villaveces A. The validity of a proxy acculturation scale among US Hispanics. Hispanic Journal of Behavioral Sciences. 2008;30(4):425–46.

[48] Werch CE. Expanding the stages of change: A program matched to the stages of alcohol acquisition. American Journal of Health Promotion. 1997;12(1):34–7. doi: 10.4278/0890-1171-12.1.34 10170433

[49] Johnston L, O’Malley P, Miech R, Bachman J, Schulenberg J. Monitoring the Future national survey results on drug use, 1975–2015: Overview, key findings on adolescent drug use. 2016.

[50] Barnes HL, Olson DH. Parent-adolescent communication and the circumplex model. Child development. 1985:438–47.

[51] Jackson KM, Roberts ME, Colby SM, Barnett NP, Abar CC, Merrill JE. Willingness to drink as a function of peer offers and peer norms in early adolescence. Journal of studies on alcohol and drugs. 2014;75(3):404–14. doi: 10.15288/jsad.2014.75.404 24766752 PMC4002854

[52] Saunders JB, Aasland OG, Babor TF, De La Fuente JR, Grant M. Development of the Alcohol Use Disorders Identification Test (AUDIT): WHO Collaborative Project on Early Detection of Persons with Harmful Alcohol Consumption-II. Addiction. 1993;88(6):791–804. doi: 10.1111/j.1360-0443.1993.tb02093.x 8329970

[53] Skinner HA. The drug abuse screening test. Addictive Behaviors. 1982;7(4):363–71. doi: 10.1016/0306-4603(82)90005-3 7183189

[54] SAMHSA.gov. 2018 National Survey of Drug Use and Health (NSDUH) Releases: CBHSQ Data.

[55] Youth.gov. Communities That Care.

[56] Gray-Little B, Williams VSL, Hancock TD. An item response theory analysis of the Rosenberg Self-Esteem Scale. Personality and social psychology bulletin. 1997;23(5):443–51.

[57] Pagano ME, Cassidy LJ, Little M, Murphy JM, Jellinek, Michael S. Identifying psychosocial dysfunction in school‐age children: The Pediatric Symptom Checklist as a self‐report measure. Psychology in the Schools. 2000;37(2):91–106. doi: 10.1002/(SICI)1520-6807(200003)37:2<91::AID-PITS1>3.0.CO;2-3 22328794 PMC3274771

[58] Robinson CC, Mandleco B, Olsen SF, Hart CH. The parenting styles and dimensions questionnaire (PSDQ). Handbook of family measurement techniques. 2001;3:319–21.

[59] Bureau TUSC. National Survey of Children’s Health (NSCH).

[60] Kärkkäinen R, Räty H, Kasanen K. Parents’ perceptions of their child’s resilience and competencies. European Journal of Psychology of Education. 2009;24(3):405–19.

[61] Ross CE, Mirowsky J. Disorder and decay: The concept and measurement of perceived neighborhood disorder. Urban Affairs Review. 1999;34(3):412–32.

[62] Baxter SD, Smith AF, Litaker MS, Baglio ML, Guinn CH, Shaffer NM. Children’s social desirability and dietary reports. Journal of nutrition education and behavior. 2004;36(2):84–9. doi: 10.1016/s1499-4046(06)60138-3 15068757 PMC1464376

[63] Reynolds WM. Development of reliable and valid short forms of the Marlowe‐Crowne Social Desirability Scale. Journal of clinical psychology. 1982;38(1):119–25.

[64] Bellg AJ, Borrelli B, Resnick B, Hecht J, Minicucci DS, Ory M, et al. Enhancing treatment fidelity in health behavior change studies: best practices and recommendations from the NIH Behavior Change Consortium. Health Psychology. 2004;23(5):443. doi: 10.1037/0278-6133.23.5.443 15367063

[65] Robb SL, Burns DS, Docherty SL, Haase JE. Ensuring treatment fidelity in a multi‐site behavioral intervention study: Implementing NIH behavior change consortium recommendations in the SMART trial. Psycho‐oncology. 2011;20(11):1193–201. doi: 10.1002/pon.1845 22012943 PMC3198011

[66] Dusenbury L, Brannigan R, Falco M, Hansen WB. A review of research on fidelity of implementation: implications for drug abuse prevention in school settings. Health education research. 2003;18(2):237–56. doi: 10.1093/her/18.2.237 12729182

[67] Spirito A, Hernandez L, Cancilliere MK, Graves H, Barnett N. Improving parenting and parent-adolescent communication to delay or prevent the onset of alcohol and drug use in young adolescents with emotional/behavioral disorders: A pilot trial. Journal of child & adolescent substance abuse. 2015;24(5):308–22. doi: 10.1080/1067828X.2013.829013 26478690 PMC4607087

[68] Little RJ, Rubin DB. Statistical analysis with missing data. John Wiley & Sons. New York. 2002.

[69] Little RJA. A Test of Missing Completely at Random for Multivariate Data with Missing Values. 1988;83(404):1198–202.

