# Supplementary material for: Randomized controlled trial to test the efficacy of a brief, communication-based, substance use preventive intervention for parents of adolescents: Protocol for the SUPPER Project (Substance Use Prevention Promoted by Eating family meals Regularly)
Source: PLoS One. 2022 Feb 2;17(2):e0263016. doi: 10.1371/journal.pone.0263016 (PMC8809599; doi:10.1371/journal.pone.0263016)
Supplement: S1 File — (PDF) [file pone.0263016.s002.pdf]

PID:	Date:	School Code:	Initials of Data Collector:
p __ __ __ __ __	D D / M M M / Y Y		

Ref #	Question	Response Options	Skip	Response
D01	We are now going to ask you a few questions about you and your household structure.			
D02	What is <u>your</u> relationship to [name of eligible child]?	1. Biological parent<br>2. Step-parent<br>3. Adoptive parent<br>4. Foster parent<br>5. Grandparent<br>6. Legal guardian<br>7. Other		
D03	Please specify <u>your</u> relationship to the child.		Only appears if D02= 7. Other	
D30	What is your gender?	1. Female<br>2. Male<br>3. Non-binary / third gender<br>4. Prefer to self-describe<br>5. Prefer not to say		
D31	Please specify:		Only appears if D30=4	
D04	We define household members as people who sleep and eat in the same residence as you most of the time.<br><br>How many people currently live in your household (include yourself and participant child)?			
D05	<u>EXCLUDING YOURSELF AND THE CHILD IN THE STUDY WITH YOU</u>, please tell me the first name of each of the people living in your household.	*List all names.*	Will be one variable household member (total is equal to D04 minus 2), excluding parent and child participant.	

D06	What is the relationship of [<u>Name written on line #xxx</u>] to the participant child?	1. Biological mother<br>2. Biological Father<br>3. Step-mother<br>4. Step-father<br>5. Adoptive mother<br>6. Adoptive father<br>7. Foster mother<br>8. Foster father<br>9. Grandmother<br>10. Grandfather<br>11. Biological sister<br>12. Biological brother<br>13. Step-sister<br>14. Step-brother<br>15. Foster sister<br>16. Foster brother<br>17. Adopted sister<br>18. Adopted brother<br>19. Other	Will appear for each person living in the household (D05) except for the parent/ guardian and child participant.	
D07	Please specify their relationship to the child.		Only appears if D06 = 19. Other	
D08	What is your current marital status?	1. Single, never married<br>2. Living with partner, unmarried<br>3. Married<br>4. Separated/divorced<br>5. Widowed		
D09	Do you consider yourself Hispanic or Latino/a?	0. No<br>1. Yes		
D10	Which of the following best describes you? *Check <u>all</u> that apply.*	1. Mexican/Mexican American<br>2. Puerto Rican<br>3. Cuban/Cuban American<br>4. Dominican<br>5. Central/South American<br>6. Other Hispanic or Latino/a	Only appears if D09 = 1. Yes	
D11	Please specify:		Only appears if D10 = 6. Other	
D12	Which of the following best describes you? *Check <u>all</u> that apply.*	1. Black or African American<br>2. White	Only appears if D09 = 0. No	

		3. American Indian or Alaskan Native<br>4. Asian<br>5. Native Hawaiian or Other Pacific Islander<br>6. Other		
D13	Please specify:		Only appears if D12 = 6. Other	
Section Header: As a reminder, study staff will protect your personal information so no one will be able to connect your responses and any other information that identifies you.				
D14	Were you born in the United States?	0. No<br>1. Yes		
D15	What country were you born in?		Only appears if D14 = 0. No	
D16	Do you speak another language in addition to English at your home?	0. No, English only<br>1. Yes, Spanish (Español)<br>2. Yes, other		
D17	Specify other language:		Only appears if D16 = 2. Yes, other	
D18	Do you speak mostly [other language] or English with <u>YOUR WIFE, HUSBAND, OR PERSON THAT YOU LIVE WITH</u> or do you use both about the same?	1. Mostly [other language]<br>2. Both about the same<br>3. Mostly English	Only appears if: D08 = 2 or 3 <u>AND</u> D16 = 1 or 2	
D19	Do you speak mostly [other language] or English with <u>YOUR CHILD(REN)</u> or do you use both about the same?	1. Mostly [other language]<br>2. Both about the same<br>3. Mostly English	Only appears if: D16 = 1 or 2	
D20	Do you speak mostly [other language] or English with <u>YOUR BROTHER/SISTERS</u> or do you use both about the same?	0. Not applicable (do not have brothers or sisters)<br>1. Mostly [other language]<br>2. Both about the same<br>3. Mostly English	Only appears if: D16 = 1 or 2	
D21	Do you speak mostly [other language] or English with <u>YOUR PARENTS</u> or do you use both about the same?	1. Mostly [other language]<br>2. Both about the same	Only appears if:	

		3. Mostly English	D16 = 1 or 2	
D22	How many years have you lived in the United States?	____________ years	Only appear of D15 = 0. No	
D23	What is your birthdate?	*dd/mmm/yyyy*		
D24	What is the highest level of education you have completed? *Check one best answer.*	1. No high school degree<br>2. High school degree<br>3. GED<br>4. Some college<br>5. 2 year degree<br>6. 4 year degree<br>7. Master's level degree<br>8. Doctorate level degree		
D25	What is the highest level of education the other adult caretaker in your household has completed? *Check one best answer.*	0. There is only one adult caretaker in the household<br>1. No high school degree<br>2. High school degree<br>3. GED<br>4. Some college<br>5. 2 year degree<br>6. 4 year degree<br>7. Master's level degree<br>8. Doctorate level degree		
D26	Please answer based on a typical work week.<br><br>I am:	1. Employed full-time (30 or more hours per week)<br>2. Employed part-time (Less than 30 hours per week)<br>3. Retired<br>4. Not employed for pay<br>5. A student		
D27	Please answer based on a typical work week.<br><br>I work:	1. Days<br>2. Nights<br>3. Both	Only shown if D26 = 1 or 2	
D28	Please answer based on a typical work week.<br><br>I work:	1. Weekdays<br>2. Weekends<br>3. Both	Only shown if D26 = 1 or 2	
D29	How much money does everyone in the household make combined? *Check one best answer.*	1. Less than $5,000<br>2. $5,000 to $9,999<br>3. $10,000 to $14,999<br>4. $15,000 to $25,999		

		5. $26,000 to $49,999<br>6. $50,000 to $74,999<br>7. $75,000 to $99,999<br>8. $100,000 to $149,000<br>9. $150,000 or more		
