# Supplementary material for: Randomized controlled trial to test the efficacy of a brief, communication-based, substance use preventive intervention for parents of adolescents: Protocol for the SUPPER Project (Substance Use Prevention Promoted by Eating family meals Regularly)
Source: PLoS One. 2022 Feb 2;17(2):e0263016. doi: 10.1371/journal.pone.0263016 (PMC8809599; doi:10.1371/journal.pone.0263016)
Supplement: S2 File — (PDF) [file pone.0263016.s003.pdf]

The SUPPER Project: Child Demographic Survey
Version 3,0, 16 May 2019

PID: c __ __ __ __ __	Date Returned: D D / M M M / Y Y	School Code:	Initials of Data Collector:

**Ref #**	**Question**	**Response Options**	**Response**
D01	Do you prefer to communicate in Spanish or English?	1. English<br>2. Spanish	
D02	What is your birthdate?	*DD/MMM/YYYY*	
D15	What is your grade?<br><br>*(If it is summer vacation, please indicate the grade you will be entering in the fall)*	1. 5th grade<br>2. 6th grade<br>3. 7th grade	
D16	What is your gender?	1. Boy<br>2. Girl<br>3. Other	
D17	Specify (*if you chose other):*		
D03	Do you consider yourself Hispanic or Latino/a?	0. No<br>1. Yes	
D04	Which of the following best describes you? *Check all that apply.*	1. Mexican/Mexican American<br>2. Puerto Rican<br>3. Cuban/Cuban American<br>4. Dominican<br>5. Central/South American<br>6. Other Hispanic or Latino/a	
D05	Please specify *(if you chose Other Hispanic or Latino/a)*:		
D06	Which of the following best describes you? *Check all that apply.*	1. Black or African American<br>2. White<br>3. American Indian or Alaskan Native<br>4. Asian<br>5. Native Hawaiian or Other Pacific Islander<br>6. Other	

D07	Please specify *(if you chose Other)*.		
D08	Were you born in the United States?	0. No<br>1. Yes	
D09	What country were you born in?		
D10	Do you speak another language in addition to English at your home?	0. No, English only<br>1. Yes, Spanish (Español)<br>2. Yes, other	
D11	Yes, other, specify language:		
D12	Do you speak mostly [*other language*] or English with <u>YOUR BROTHER/SISTERS</u> or do you use both about the same?	0. Not applicable (do not have brothers or sisters)<br>1. Mostly [*other language*]<br>2. Both about the same<br>3. Mostly English	
D13	Do you speak mostly [*other language*] or English with <u>YOUR PARENTS</u> or do you use both about the same?	1. Mostly [*other language*]<br>2. Both about the same<br>3. Mostly English	
D14	How many years have you lived in the United States?	___________ years	
