# Supplementary material for: Randomized controlled trial to test the efficacy of a brief, communication-based, substance use preventive intervention for parents of adolescents: Protocol for the SUPPER Project (Substance Use Prevention Promoted by Eating family meals Regularly)
Source: PLoS One. 2022 Feb 2;17(2):e0263016. doi: 10.1371/journal.pone.0263016 (PMC8809599; doi:10.1371/journal.pone.0263016)
Supplement: S3 File — (PDF) [file pone.0263016.s004.pdf]

**CID:**	**Date:** D D M M M Y Y	**School Code:**	**Initials of Data Collector:**

CID:	Date: D D M M M Y Y	School Code:	Initials of Data Collector:

Var Name	Item	Response Options	Response
cintro	Thank you for taking the time to complete this survey. Your responses are very important to us. Please read each question carefully before answering.<br><br>Filling out this survey is your choice. You do not have to answer any questions unless you choose to. All the information you share with us will be kept private. It will not be seen by anyone, besides the people working on the study.<br><br>The survey is expected to take about **1 hour** to complete. Try to complete the entire survey in one sitting. If you need to stop and finish the survey at a later time, you can do so.<br><br>If you have any questions about the survey, please contact our study team at **(617) 636-3587** or e-mail us at thesupperproject@tufts.edu .<br><br>Throughout the survey, when we say "parent" we mean your parent or guardian that is in the study with you.<br><br>When taking this survey, you agree to:<br><br>1. Complete the survey yourself without assistance from anyone, except a study team member;<br>2. Not share your responses with your parent; and<br>3. Not watch or help your parent complete their survey<br><br>**By clicking "continue" you agree to the above statements.**<br><br>Thank you for your help!		

Var Name	Item	Response Options	Response
cpracintro01	We are going to walk you through a few practice questions to make sure you feel comfortable completing the survey in this format.<br><br>Throughout the survey, if you skip a question you will be asked whether you skipped the question on purpose or if it was a mistake. If it was a mistake, you will be able to answer		

CID:	Date: DD MMM YY	School Code:	Initials of Data Collector:

	the question. If you skipped the question because you did not want to answer it, you can leave it blank.		
cprac01	Which is your favorite season?<br><br>If winter is your favorite season, you can choose the "Other" button and type your answer in the text box.	1. Spring<br>2. Summer<br>3. Fall<br>4. Other	
cprac01_spec	Please specify:		
cprac02	This is an example of a question where you can choose more than one answer. "Check all that apply" will tell you when you can select more than one answer.<br><br>Which of the following are shapes? (Check all that apply)	1. Triangle<br>2. Dog<br>3. Circle<br>4. Square	
cpracintro03	The next question is a practice grid question. It is important that you make a choice for each row before clicking on the "next" button.<br><br>How much do you agree with the following statements? (Select one answer for each line)		
cprac03	Winter is cold	1. Strongly disagree<br>2. Disagree<br>3. Somewhat disagree<br>4. Somewhat agree<br>5. Agree<br>6. Strongly agree	
cprac04	Dancing is fun	1. Strongly disagree<br>2. Disagree<br>3. Somewhat disagree<br>4. Somewhat agree<br>5. Agree<br>6. Strongly agree	
cprac05	Pizza is delicious	1. Strongly disagree	

CID:	Date: DD MMM YY	School Code:	Initials of Data Collector:

		2. Disagree<br>3. Somewhat disagree<br>4. Somewhat agree<br>5. Agree<br>6. Strongly agree	
cprac06_hours	In the last practice question, you need to type in the amount of time in the format below.<br><br>About how much time does it take you to get to school?<br><br>Number of hours to get to school:	*Write number of hours.*	
cprac06_min	Number of minutes to get to school:	*Write number of minutes.*	
	The survey will now begin.		

Section 1: Household structure

Var Name	Item	Response Options	Response
chsfintro	We define household members as people who sleep and eat in the same residence as you most of the time.		
chsf01	How many people currently live in your household (include yourself)?	*Number value.*	
chsf02	For each person living in the household with you, list their name and the corresponding number of their relationship <u>to you</u>.<br><br>1. Biological mother<br>2. Biological Father<br>3. Step-mother	*For example: You live with your mother (Lisa), and little brother (John).*<br><br>*HH Member 1 Name: Lisa*<br>*Relationship: 1*<br><br>*HH Member 2 Name: John*<br>*Relationship of HH Member 2: 12*	HH Member #1 Name:<br>Relationship HH Member #1:<br><br>HH Member #2 Name:<br>Relationship HH Member #2:<br><br>HH Member #3 Name:<br>Relationship HH Member #3:

CID:	Date: D D M M M Y Y	School Code:	Initials of Data Collector:

	4. Step-father<br>5. Adoptive mother<br>6. Adoptive father<br>7. Foster mother<br>8. Foster father<br>9. Grandmother<br>10. Grandfather<br>11. Biological sister<br>12. Biological brother<br>13. Step-sister<br>14. Step-brother<br>15. Foster sister<br>16. Foster brother<br>17. Adopted sister<br>18. Adopted brother<br>19. Other and specify	*It does not matter the order that you list your household members.*<br><br>*If you respond "19. Other", specify the relationship.*	HH Member #4 Name:<br>Relationship HH Member #4:<br><br>HH Member #5 Name:<br>Relationship HH Member #5:<br><br>HH Member #6 Name:<br>Relationship HH Member #6:<br><br>HH Member #7 Name:<br>Relationship HH Member #7:<br><br>HH Member #8 Name:<br>Relationship HH Member #8:<br><br>HH Member #9 Name:<br>Relationship HH Member #9:<br><br>HH Member #10 Name:<br>Relationship HH Member #10:

CID:	Date: DD MMM YY	School Code:	Initials of Data Collector:

Section 2: COMMUNICATION
2.1 Parent-Adolescent Communication Scale

Var Name	Item	Response Options	Response
cpacintro01	Using the scale below, please indicate how much you agree or disagree with each of the following statements about the general communication between you and your parent/guardian in the study with you in the past [time point].		
cpac01	I can discuss my beliefs with my parent without feeling restrained or embarrassed.	1. Strongly disagree<br>2. Disagree<br>3. Somewhat disagree<br>4. Somewhat agree<br>5. Agree<br>6. Strongly agree	
cpac02	Sometimes I have trouble believing everything my parent tells me.	1. Strongly disagree<br>2. Disagree<br>3. Somewhat disagree<br>4. Somewhat agree<br>5. Agree<br>6. Strongly agree	
cpac03	My parent is always a good listener.	1. Strongly disagree<br>2. Disagree<br>3. Somewhat disagree<br>4. Somewhat agree<br>5. Agree<br>6. Strongly agree	
cpac04	I am sometimes afraid to ask my parent for what I want.	1. Strongly disagree<br>2. Disagree<br>3. Somewhat disagree<br>4. Somewhat agree<br>5. Agree<br>6. Strongly agree	
cpac05	My parent has a tendency to say things which would be better left unsaid.	1. Strongly disagree	

CID:	Date: DD MMM YY	School Code:	Initials of Data Collector:

		2. Disagree<br>3. Somewhat disagree<br>4. Somewhat agree<br>5. Agree<br>6. Strongly agree	
cpac06	My parent can tell how I am feeling without asking.	1. Strongly disagree<br>2. Disagree<br>3. Somewhat disagree<br>4. Somewhat agree<br>5. Agree<br>6. Strongly agree	
cpac07	I am very satisfied with how my parent and I talk together.	1. Strongly disagree<br>2. Disagree<br>3. Somewhat disagree<br>4. Somewhat agree<br>5. Agree<br>6. Strongly agree	
cpac08	If I were in trouble, I could tell my parent.	1. Strongly disagree<br>2. Disagree<br>3. Somewhat disagree<br>4. Somewhat agree<br>5. Agree<br>6. Strongly agree	
cpac09	I openly show affection to my parent.	1. Strongly disagree<br>2. Disagree<br>3. Somewhat disagree<br>4. Somewhat agree<br>5. Agree<br>6. Strongly agree	
cpac10	When we are having a problem, I often give my parent the silent treatment.	1. Strongly disagree<br>2. Disagree<br>3. Somewhat disagree	

CID:	Date: D D M M M Y Y	School Code:	Initials of Data Collector:

		4. Somewhat agree<br>5. Agree<br>6. Strongly agree	
cpac11	I am careful about what I say to my parent.	1. Strongly disagree<br>2. Disagree<br>3. Somewhat disagree<br>4. Somewhat agree<br>5. Agree<br>6. Strongly agree	
cpac12	When I am talking to my parent, I have a tendency to say things that would be better left unsaid.	1. Strongly disagree<br>2. Disagree<br>3. Somewhat disagree<br>4. Somewhat agree<br>5. Agree<br>6. Strongly agree	
cpac13	When I ask questions, I get honest answers from my parent.	1. Strongly disagree<br>2. Disagree<br>3. Somewhat disagree<br>4. Somewhat agree<br>5. Agree<br>6. Strongly agree	
cpac14	My parent tries to understand my point of view.	1. Strongly disagree<br>2. Disagree<br>3. Somewhat disagree<br>4. Somewhat agree<br>5. Agree<br>6. Strongly agree	
cpac15	There are topics I avoid discussing with my parent.	1. Strongly disagree<br>2. Disagree<br>3. Somewhat disagree<br>4. Somewhat agree<br>5. Agree	

CID:	Date: DD MMM YY	School Code:	Initials of Data Collector:

		6. Strongly agree	
cpac16	I find it easy to discuss problems with my parent.	1. Strongly disagree<br>2. Disagree<br>3. Somewhat disagree<br>4. Somewhat agree<br>5. Agree<br>6. Strongly agree	
cpac17	It is very easy for me to express all my true feelings to my parent.	1. Strongly disagree<br>2. Disagree<br>3. Somewhat disagree<br>4. Somewhat agree<br>5. Agree<br>6. Strongly agree	
cpac18	My parent nags/bothers me.	1. Strongly disagree<br>2. Disagree<br>3. Somewhat disagree<br>4. Somewhat agree<br>5. Agree<br>6. Strongly agree	
cpac19	My parent insults me when they are angry with me.	1. Strongly disagree<br>2. Disagree<br>3. Somewhat disagree<br>4. Somewhat agree<br>5. Agree<br>6. Strongly agree	
cpac20	I don't think I can tell my parent how I really feel about some things.	1. Strongly disagree<br>2. Disagree<br>3. Somewhat disagree<br>4. Somewhat agree<br>5. Agree<br>6. Strongly agree	

CID:	Date: DD MMM YY	School Code:	Initials of Data Collector:

2.2 Frequency of parent-child communication about substance use

Var Name	Item	Response Options	Response
cfpcintro	Below are a few topics you may or may not have ever discussed with your parent/guardian in the study with you.<br><br>**DO NOT include the audio-recorded conversation you had with your parent using the Parent-Child Conversation Prompts for the SUPPER Project.**		
cfpc01	Have you and your parent ever talked about smoking cigarettes?	0. No<br>1. Yes	
cfpc02	During the past [time_point], how many times have you and your parent talked about smoking cigarettes?	0. None<br>1. Once<br>2. A few times<br>3. Several<br>4. A lot	
cfpc03	Have you and your parent ever talked about using e-cigarettes or vaping?	0. No<br>1. Yes	
cfpc04	During the past [time_point], how many times have you and your parent talked about using e-cigarettes or vaping?	0. None<br>1. Once<br>2. A few times<br>3. Several<br>4. A lot	
cfpc05	Have you and your parent ever talked about drinking alcohol?	0. No<br>1. Yes	
cfpc06	During the past [time_point], how many times have you and your parent talked about drinking alcohol?	0. None<br>1. Once<br>2. A few times<br>3. Several<br>4. A lot	
cfpc07	Have you and your parent ever talked about using marijuana?	0. No<br>1. Yes	
cfpc08	During the past [time_point], how many times have you and your parent talked about using marijuana?	0. None	

CID:	Date: DD MMM YY	School Code:	Initials of Data Collector:

		1. Once<br>2. A few times<br>3. Several<br>4. A lot	
cfpc09	Have you and your parent ever talked about using other drugs?	0. No<br>1. Yes	
cfpc10	During the past [time_point], how many times have you and your parent talked about using other drugs?	0. None<br>1. Once<br>2. A few times<br>3. Several<br>4. A lot	

2.3 Other Conversations about Drugs/Alcohol

Var Name	Item	Response Options	Response
cocd01	Have you ever talked to anyone else (other than the parent/guardian in the study with you) about alcohol, marijuana, or other drugs?	0. No<br>1. Yes	
cocd02	If so, who? (Check all that apply.)	1. Friends<br>2. Teacher<br>3. Sibling<br>4. Other person or relative	
cocd02_spec	Please specify the other person(s) relationship to you (for example: aunt, uncle, grandparent, nurse, etc.)		
cocd03	During the past [time_point], have you ever talked to anyone else (other than the parent/guardian in the study with you) about alcohol, marijuana, or other drugs?	0. No<br>1. Yes	
cocd04	If so, who? (Check all that apply)	1. Friends<br>2. Teacher<br>3. Sibling<br>4. Other person or relative	
cocd04_spec	Please specify who and include their relationship to you (for example: friend, aunt, uncle, grandparent, etc.)		

CID:	Date: DD MMM YY	School Code:	Initials of Data Collector:

2.4 Targeted Parent-Child Communication About Drugs

Var Name	Item	Response Options	Response
ctpcintro	Think about the conversations you have had with your parent/**guardian**, the one in the study with you, in the past [time_point]. With these conversations in mind, **indicate how much you agree or disagree with each of the following statements.**<br><br>**DO NOT include the audio-recorded conversation you had with your parent using the Parent-Child Conversation Prompts for the SUPPER Project.**<br><br>The parent in the study with me…		
ctpc01	Has not directly talked with me about drugs and alcohol use but has given hints that I should not use them.	1. Strongly disagree<br>2. Disagree<br>3. Somewhat disagree<br>4. Somewhat agree<br>5. Agree<br>6. Strongly agree	
ctpc02	Has lectured me or given me a speech about drinking alcohol and using drugs.	1. Strongly disagree<br>2. Disagree<br>3. Somewhat disagree<br>4. Somewhat agree<br>5. Agree<br>6. Strongly agree	
ctpc03	Has warned me about the dangers of drinking alcohol and using drugs	1. Strongly disagree<br>2. Disagree<br>3. Somewhat disagree<br>4. Somewhat agree<br>5. Agree<br>6. Strongly agree	
ctpc04	Has talked to me about how to handle offers of alcoholic drinks and drugs.	1. Strongly disagree<br>2. Disagree<br>3. Somewhat disagree<br>4. Somewhat agree<br>5. Agree<br>6. Strongly agree	
ctpc05	Has given me rules to obey about drinking alcohol and using drugs.	1. Strongly disagree	

CID:	Date: DD MMM YY	School Code:	Initials of Data Collector:

		2. Disagree<br>3. Somewhat disagree<br>4. Somewhat agree<br>5. Agree<br>6. Strongly agree	
ctpc06	Will make a comment about how drinking alcohol and using drugs is bad if a character on TV is drinking or drunk.	1. Strongly disagree<br>2. Disagree<br>3. Somewhat disagree<br>4. Somewhat agree<br>5. Agree<br>6. Strongly agree	
ctpc07	Tells me stories of people who drink alcohol, have been drunk, or use drugs.	1. Strongly disagree<br>2. Disagree<br>3. Somewhat disagree<br>4. Somewhat agree<br>5. Agree<br>6. Strongly agree	
ctpc08	Tells me they would be disappointed in me if I drink alcohol or use drugs	1. Strongly disagree<br>2. Disagree<br>3. Somewhat disagree<br>4. Somewhat agree<br>5. Agree<br>6. Strongly agree	
ctpc09	Shows me information on the web, TV, or in the news about the dangers of drinking alcohol and using drugs.	1. Strongly disagree<br>2. Disagree<br>3. Somewhat disagree<br>4. Somewhat agree<br>5. Agree<br>6. Strongly agree	
ctpc10	Asks about my thoughts and opinions about drinking alcohol and using drugs	1. Strongly disagree<br>2. Disagree<br>3. Somewhat disagree<br>4. Somewhat agree<br>5. Agree<br>6. Strongly agree	

CID:	Date: DD MMM YY	School Code:	Initials of Data Collector:

2.5 Comfort level talking about drugs/alcohol

Var Name	Item	Response Options	Response
ccltintro	Think about <u>all</u> the conversations you have had with your parent/guardian in the study with you about alcohol and drugs.		
cclt01	How comfortable do <u>you</u> feel when you discuss cigarettes with your parent/guardian?	1. I feel very uncomfortable<br>2. I feel a little uncomfortable<br>3. I feel somewhat comfortable<br>4. I feel very comfortable	
cclt02	How comfortable do <u>you</u> feel when you discuss e-cigarettes with your parent/guardian?	1. I feel very uncomfortable<br>2. I feel a little uncomfortable<br>3. I feel somewhat comfortable<br>4. I feel very comfortable	
cclt03	How comfortable do <u>you</u> feel when you discuss alcohol with your parent/guardian?	1. I feel very uncomfortable<br>2. I feel a little uncomfortable<br>3. I feel somewhat comfortable<br>4. I feel very comfortable	
cclt04	How comfortable do <u>you</u> feel when you discuss marijuana with your parent/guardian?	1. I feel very uncomfortable<br>2. I feel a little uncomfortable<br>3. I feel somewhat comfortable<br>4. I feel very comfortable	
cclt05	How comfortable do <u>you</u> feel when you discuss other drugs with your parent/guardian?	1. I feel very uncomfortable<br>2. I feel a little uncomfortable<br>3. I feel somewhat comfortable<br>4. I feel very comfortable	
cclt06	How comfortable do you think <u>your parent/guardian</u> feels when they discuss cigarettes with you?	1. They feel very uncomfortable<br>2. They feel a little uncomfortable<br>3. They feel somewhat comfortable<br>4. They feel very comfortable	
cclt07	How comfortable do you think <u>your parent/guardian</u> feels when they discuss e-cigarettes with you?	1. They feel very uncomfortable<br>2. They feel a little uncomfortable<br>3. They feel somewhat comfortable<br>4. They feel very comfortable	
cclt08	How comfortable do you think <u>your parent/guardian</u> feels when they discuss alcohol with you?	1. They feel very uncomfortable<br>2. They feel a little uncomfortable<br>3. They feel somewhat comfortable<br>4. They feel very comfortable	

**CID:**	**Date:** DD MMM YY	**School Code:**	**Initials of Data Collector:**

cclt09	How comfortable do you think your parent/guardian feels when they discusses marijuana with you?	1. They feel very uncomfortable<br>2. They feel a little uncomfortable<br>3. They feel somewhat comfortable<br>4. They feel very comfortable	
cclt10	How comfortable do you think your parent/guardian feels when they discuss other drugs with you?	1. They feel very uncomfortable<br>2. They feel a little uncomfortable<br>3. They feel somewhat comfortable<br>4. They feel very comfortable	

CID:	Date: DD MMM YY	School Code:	Initials of Data Collector:

2.6 Health/Weight-Related Conversations

Var Name	Item	Response Options	Response
chwrcintro	The following set of questions are about conversations you have had with your parent/guardian in the study with you about eating habits or physical activity.<br><br>**DO NOT include the audio-recorded conversation you had with your parent using the Parent-Child Conversation Prompts for the SUPPER Project.**		
chwrc01	How often in the past [time_point] has your parent had a conversation with you about healthy eating habits?	0. Never<br>1. A few times in the past [time_point]<br>2. A few times a month<br>3. A few times a week<br>4. Almost every day	
chwrc02	How often in the past [time_point] has your parent had a conversation with you about being physically active?	0. Never<br>1. A few times in the past [time_point]<br>2. A few times a month<br>3. A few times a week<br>4. Almost every day	
chwrc03	How often in the past [time_point] has your parent had a conversation with you about your weight or size?	0. Never<br>1. A few times in the past [time_point]<br>2. A few times a month<br>3. A few times a week<br>4. Almost every day	
chwrc04	How often in the past [time_point] has your parent mentioned to you that you weigh too much?	0. Never<br>1. A few times in the past [time_point]<br>2. A few times a month<br>3. A few times a week<br>4. Almost every day	
chwrc05	How often in the past [time_point] has your parent mentioned to you that you should eat differently in order to lose weight or keep from gaining weight?	0. Never<br>1. A few times in the past [time_point]<br>2. A few times a month<br>3. A few times a week<br>4. Almost every day	
chwrc06	How often in the past [time_point] has your parent mentioned to you that you should exercise in order to lose weight or to keep from gaining weight?	0. Never<br>1. A few times in the past [time_point]	

CID:	Date: D D M M M Y Y	School Code:	Initials of Data Collector:

		2. A few times a month<br>3. A few times a week<br>4. Almost every day	

2.7 Negative weight talk

Var Name	Item	Response Options	Response
cnwtintro	How often in the past [time_point] has your parent/guardian in the study with you done or said the following to you:		
cnwt01	Said you were fat?	0. Never<br>1. A few times in the past [time_point]<br>2. A few times a month<br>3. A few times a week<br>4. Almost every day	
cnwt02	Teased or made fun of you about the size and shape of your body?	0. Never<br>1. A few times in the past [time_point]<br>2. A few times a month<br>3. A few times a week<br>4. Almost every day	
cnwt03	Said you should go on a diet?	0. Never<br>1. A few times in the past [time_point]2. A few times a month<br>3. A few times a week<br>4. Almost every day	
cnwt04	Said that you eat food that will make you fat?	0. Never<br>1. A few times in the past [time_point]<br>2. A few times a month<br>3. A few times a week<br>4. Almost every day	
cnwt05	Said that you would look better if you were thinner?	0. Never<br>1. A few times in the past [time_point]<br>2. A few times a month<br>3. A few times a week<br>4. Almost every day	

**CID:**	**Date:** DD MMM YY	**School Code:**	**Initials of Data Collector:**

csnwt01	Said **about themselves** that **they** were fat or that **they** needed to go on a diet to lose weight?	0. Never<br>1. A few times in the past [time_point]<br>2. A few times a month<br>3. A few times a week<br>4. Almost every day	
cnwtintro02	How often in the past [time_point] has **your other parent/guardian (the one NOT in the study with you)** done or said the following to you:	0. I do **not** have another parent/guardian besides the one in the study with me<br>1. I have another parent/guardian besides the one in the study with me	
cnwt06	Said you were fat?	0. Never<br>1. A few times in the past [time_point]<br>2. A few times a month<br>3. A few times a week<br>4. Almost every day	
cnwt07	Teased or made fun of you about the size and shape of your body?	0. Never<br>1. A few times in the past [time_point]<br>2. A few times a month<br>3. A few times a week<br>4. Almost every day	
cnwt08	Said you should go on a diet?	0. Never<br>1. A few times in the past [time_point]<br>2. A few times a month<br>3. A few times a week<br>4. Almost every day	
cnwt09	Said that you eat food that will make you fat?	0. Never<br>1. A few times in the past [time_point]<br>2. A few times a month<br>3. A few times a week<br>4. Almost every day	
cnwt10	Said that you would look better if you were thinner?	0. Never<br>1. A few times in the past [time_point]<br>2. A few times a month<br>3. A few times a week<br>4. Almost every day	

**CID:**	**Date:** DD MMM YY	**School Code:**	**Initials of Data Collector:**

csnwt02	Said **about themselves** that **they** were fat or that **they** needed to go on a diet to lose weight?	0. Never<br>1. A few times in the past [time_point]<br>2. A few times a month<br>3. A few times a week<br>4. Almost every day	
cnwtintro03	How often in the past [time_point] has/have **your sibling/s or other child/ren living in your house** done or said the following to you:	0. I do **not** have any siblings or other children living with me<br>1. I do have siblings or other children living with me	
cnwt11	Said you were fat?	0. Never<br>1. A few times in the past [time_point]<br>2. A few times a month<br>3. A few times a week<br>4. Almost every day	
cnwt12	Teased or made fun of you about the size and shape of your body?	0. Never<br>1. A few times in the past [time_point]<br>2. A few times a month<br>3. A few times a week<br>4. Almost every day	
cnwt13	Said you should go on a diet?	0. Never<br>1. A few times in the past [time_point]<br>2. A few times a month<br>3. A few times a week<br>4. Almost every day	
cnwt14	Said that you eat food that will make you fat?	0. Never<br>1. A few times in the past [time_point]<br>2. A few times a month<br>3. A few times a week<br>4. Almost every day	
cnwt15	Said that you would look better if you were thinner?	0. Never<br>1. A few times in the past [time_point]<br>2. A few times a month<br>3. A few times a week<br>4. Almost every day	

CID:	Date: DD MMM YY	School Code:	Initials of Data Collector:

csnwt03	<u>Said</u> **<u>about themselves</u>** that **they** were fat or that **they** needed to go on a diet to lose weight?	0. Never<br>1. A few times in the past <u>[time_point]</u><br>2. A few times a month<br>3. A few times a week<br>4. Almost every day	

Section 3: EATING, EXERCISE, & WEIGHT
3.1 Dietary Intake

Var Name	Item	Response Options	Response
cdiintro	The next 8 questions ask about food and beverages you ate or drank during the past 7 days. Think about all the meals and snacks you had from the time you got up until you went to bed. Be sure to include food you ate at home, at school, at restaurants, or anywhere else.		
cdi01	During the <u>past 7 days</u>, how many times did you eat <u>fruit</u>? (Do <u>not</u> count fruit juice.)	0. None<br>1. 1-3 times<br>2. 4-6 times<br>3. 1 time per day<br>4. 2 times per day<br>5. 3 times per day<br>6. 4 or more times per day	
cdi02	During the <u>past 7 days</u>, how many times did you drink <u>100% fruit juice</u> such as orange juice, apple juice, or grape juice? (Do <u>not</u> count punch, Kool-Aid, sports drinks, or other fruit-flavored drinks.)	0. None<br>1. 1-3 times<br>2. 4-6 times<br>3. 1 time per day<br>4. 2 times per day<br>5. 3 times per day<br>6. 4 or more times per day	
cdi03	During the <u>past 7 days</u>, how many times did you eat <u>vegetables</u> (for example, green salad, carrots or any other vegetable—do <u>not</u> include French fries or potato chips)?	0. None<br>1. 1-3 times<br>2. 4-6 times<br>3. 1 time per day<br>4. 2 times per day	

CID:	Date: D D M M M Y Y	School Code:	Initials of Data Collector:

		5. 3 times per day<br>6. 4 or more times per day	
cdi04	During the past 7 days, how many times did you drink a can, bottle, or glass of soda or pop, such as Coke, Pepsi, or Sprite? (Do not count diet soda or diet pop.)	0. None<br>1. 1-3 times<br>2. 4-6 times<br>3. 1 time per day<br>4. 2 times per day<br>5. 3 times per day<br>6. 4 or more times per day	
cdi05	During the past 7 days, how many times did you drink a can, bottle, or glass of a sports drink such as Gatorade or Powerade? (Do not count low-calorie sports drinks such as Propel or G2.)	0. None<br>1. 1-3 times<br>2. 4-6 times<br>3. 1 time per day<br>4. 2 times per day<br>5. 3 times per day<br>6. 4 or more times per day	
cdi06	During the past 7 days, how many times did you drink a bottle or glass of plain water (count tap, bottled, and unflavored sparkling water)?	0. None<br>1. 1-3 times<br>2. 4-6 times<br>3. 1 time per day<br>4. 2 times per day<br>5. 3 times per day<br>6. 4 or more times per day	
cdi07	During the past 7 days, how many times did you drink a glass of milk? (Count the milk you drank in a glass or cup, from a carton, or with cereal. Count the half pint of milk served at school as equal to one glass).	0. None<br>1. 1-3 times<br>2. 4-6 times<br>3. 1 time per day<br>4. 2 times per day<br>5. 3 times per day<br>6. 4 or more times per day	
cdi08	During the past 7 days, on how many days did you eat breakfast?	0. Never<br>1. 1 day	

CID:	Date: DD MMM YY	School Code:	Initials of Data Collector:

		2 days<br>3 days<br>4 days<br>5 days<br>6 days<br>7 days	

3.2 Physical Activity Levels

Var Name	Item	Response Options	Response
cpal01	The following two questions ask about your physical activity and exercise habits.<br>During the <u>past 7 days</u>, on how many days were you physically active for a total of at least 60 minutes per day? (Add up all the time you spent in any kind of physical activity that increased your heart rate and made you breathe hard some of the time).	0. 0 days<br>1. 1 day<br>2. 2 days<br>3. 3 days<br>4. 4 days<br>5. 5 days<br>6. 6 days<br>7. 7 days	
cpal02	During the past <u>[time_point]</u>, on how many sports teams did you play?	0. 0 teams<br>1. 1 team<br>2. 2 teams<br>3. 3 or more teams	

3.3 Child's own weight description

Var Name	Item	Response Options	Response
cwd01	The next few questions will ask you about your current weight. It is possible that answering some of these questions might make you uncomfortable. Please remember that there are no right or wrong answers and all of your answers will remain private.<br><br>How do you describe your weight?	1. Very underweight<br>2. Slightly underweight<br>3. About the right weight<br>4. Slightly overweight<br>5. Very overweight	
cwd02	Which of the following are you trying to do about your weight?	1. Lose weight	

CID:	Date: DD MMM YY	School Code:	Initials of Data Collector:

		2. Gain weight<br>3. Stay the same weight<br>4. I am not trying to do anything about my weight	

3.4 Weight Bias Internalization Scale - Modified

Var Name	Item	Response Options	Response
cwbismintro	The following statements are about how you feel about **your current weight.**<br><br>Read each statement below and answer with how much you agree or disagree with each statement.		
cwbism01	I am less attractive than most other people because of my weight.	1. Strongly disagree<br>2. Disagree<br>3. Somewhat disagree<br>4. Somewhat agree<br>5. Agree<br>6. Strongly agree	
cwbism02	I feel anxious about my weight because of what other people might think of me.	1. Strongly disagree<br>2. Disagree<br>3. Somewhat disagree<br>4. Somewhat agree<br>5. Agree<br>6. Strongly agree	
cwbism03	I wish I could drastically change my weight.	1. Strongly disagree<br>2. Disagree<br>3. Somewhat disagree<br>4. Somewhat agree<br>5. Agree<br>6. Strongly agree	
cwbism04	Whenever I think a lot about my weight, I feel depressed.	1. Strongly disagree<br>2. Disagree<br>3. Somewhat disagree	

CID:	Date: DD MMM YY	School Code:	Initials of Data Collector:

		4. Somewhat agree<br>5. Agree<br>6. Strongly agree	
cwbism05	I hate myself for my weight.	1. Strongly disagree<br>2. Disagree<br>3. Somewhat disagree<br>4. Somewhat agree<br>5. Agree<br>6. Strongly agree	
cwbism06	My weight is a major way that I judge my value as a person.	1. Strongly disagree<br>2. Disagree<br>3. Somewhat disagree<br>4. Somewhat agree<br>5. Agree<br>6. Strongly agree	
cwbism07	I don't feel that I deserve to have a really fulfilling social life because of my weight.	1. Strongly disagree<br>2. Disagree<br>3. Somewhat disagree<br>4. Somewhat agree<br>5. Agree<br>6. Strongly agree	
cwbism08	I am OK being the weight that I am.	1. Strongly disagree<br>2. Disagree<br>3. Somewhat disagree<br>4. Somewhat agree<br>5. Agree<br>6. Strongly agree	
cwbism09	Because of my weight, I don't feel like my true self.	1. Strongly disagree<br>2. Disagree<br>3. Somewhat disagree<br>4. Somewhat agree<br>5. Agree	

CID:	Date: DD MMM YY	School Code:	Initials of Data Collector:

		6. Strongly agree	
cwbism10	Because of my weight, I don't understand how anyone attractive would want to like me or date me.	1. Strongly disagree<br>2. Disagree<br>3. Somewhat disagree<br>4. Somewhat agree<br>5. Agree<br>6. Strongly agree	

3.5 Weight stigma experience by peers

Var Name	Item	Response Options	Response
cwsp01	Have you ever been teased or made fun of by other people your age because of your weight?	0. No<br>1. Yes	
cwsp02	During the past [time_point], have you been teased or made fun of by other people your age because of your weight?	0. No<br>1. Yes	
cwsp03	Have you ever been treated unkindly by other people your age because of your weight?	0. No<br>1. Yes	
cwsp04	During the past [time_point], have you been treated unkindly by other people your age because of your weight?	0. No<br>1. Yes	

3.6 Weight stigma experience by family members

Var Name	Item	Response Options	Response
cwsf01	Have you ever been teased or made fun of by family members because of your weight?	0. No<br>1. Yes	
cwsf02	During the past [time_point], have you been teased or made fun of by family members because of your weight?	0. No<br>1. Yes	

CID:	Date: DD MMM YY	School Code:	Initials of Data Collector:

cwsf03	Have you ever been treated unkindly by family members because of your weight?	0. No<br>1. Yes	
cwsf04	During the past [time_point], have you been treated unkindly by family members because of your weight?	0. No<br>1. Yes	

Section 4: USING SUBSTANCES

4.1 Cigarette Definition

Var Name	Item	Response Options	Response
ccdintro01	The following set of questions will be about cigarettes. By cigarettes we mean tobacco cigarettes, this does NOT INCLUDE e-cigarettes (vaping/JUULing), hookah, or other tobacco products.<br>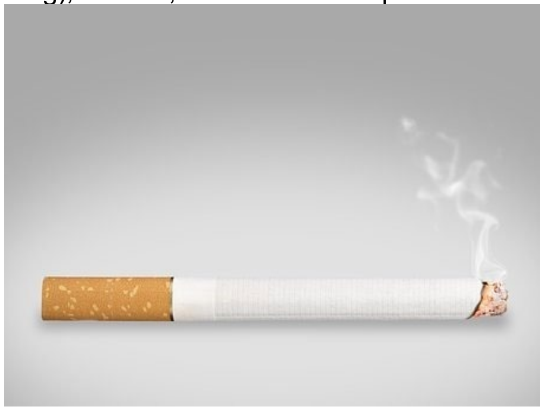		

4.2 Smoking Expectancies (Positive and Negative Outcome Expectancies of Smoking)

Var Name	Item	Response Options	Response
ccseintro01	You do not need to have any experience smoking cigarettes to answer these questions.		

CID:	Date: DD MMM YY	School Code:	Initials of Data Collector:

	Answer each statement based on YOUR own thoughts, feelings, and beliefs about cigarettes. We are interested in what you think about cigarettes, not what others might think.<br><br>Using the scale below, please indicate how much you agree or disagree with each of the following statements.		
ccse01	I think I would enjoy smoking.	1. Strongly disagree<br>2. Disagree<br>3. Somewhat disagree<br>4. Somewhat agree<br>5. Agree<br>6. Strongly agree	
ccse02	I think smoking would give me something to do when I'm bored.	1. Strongly disagree<br>2. Disagree<br>3. Somewhat disagree<br>4. Somewhat agree<br>5. Agree<br>6. Strongly agree	
ccse03	I think smoking would make my teeth yellow.	1. Strongly disagree<br>2. Disagree<br>3. Somewhat disagree<br>4. Somewhat agree<br>5. Agree<br>6. Strongly agree	
ccse04	I think smoking would help me to deal with problems or stress.	1. Strongly disagree<br>2. Disagree<br>3. Somewhat disagree<br>4. Somewhat agree<br>5. Agree<br>6. Strongly agree	
ccse05	I think smoking would help me stay thin.	1. Strongly disagree<br>2. Disagree<br>3. Somewhat disagree<br>4. Somewhat agree<br>5. Agree<br>6. Strongly agree	
ccse06	I think smoking would make it harder for me to play sports.	1. Strongly disagree	

CID:	Date: DD MMM YY	School Code:	Initials of Data Collector:

		2. Disagree<br>3. Somewhat disagree<br>4. Somewhat agree<br>5. Agree<br>6. Strongly agree	
ccse07	I think smoking would help me feel more comfortable at parties.	1. Strongly disagree<br>2. Disagree<br>3. Somewhat disagree<br>4. Somewhat agree<br>5. Agree<br>6. Strongly agree	
ccse08	I think smoking would be relaxing.	1. Strongly disagree<br>2. Disagree<br>3. Somewhat disagree<br>4. Somewhat agree<br>5. Agree<br>6. Strongly agree	
ccse09	If I started smoking regularly, I think it would be very hard for me to stop.	1. Strongly disagree<br>2. Disagree<br>3. Somewhat disagree<br>4. Somewhat agree<br>5. Agree<br>6. Strongly agree	
ccse10	I think smoking would make me look more mature.	1. Strongly disagree<br>2. Disagree<br>3. Somewhat disagree<br>4. Somewhat agree<br>5. Agree<br>6. Strongly agree	
ccse11	I think smoking would give me bad breath.	1. Strongly disagree<br>2. Disagree<br>3. Somewhat disagree<br>4. Somewhat agree<br>5. Agree<br>6. Strongly agree	
ccse12	I think smoking would be bad for my health.	1. Strongly disagree<br>2. Disagree	

CID:	Date: DD MMM YY	School Code:	Initials of Data Collector:

		3. Somewhat disagree<br>4. Somewhat agree<br>5. Agree<br>6. Strongly agree	

4.3 Cigarette Willingness

Var Name	Item	Response Options	Response
ccwintro01	Suppose you were with some friends and one of them offered you cigarettes.		
ccw01	How likely is it that you would take it and try it?	1. Not at all likely<br>2. Unlikely<br>3. Somewhat unlikely<br>4. Somewhat likely<br>5. Likely<br>6. Very likely	
ccw02	How likely is it that you would tell them "no"?	1. Not at all likely<br>2. Unlikely<br>3. Somewhat unlikely<br>4. Somewhat likely<br>5. Likely<br>6. Very likely	
ccw03	How likely is it that you would leave the situation?	1. Not at all likely<br>2. Unlikely<br>3. Somewhat unlikely<br>4. Somewhat likely<br>5. Likely<br>6. Very likely	

4.4 Cigarette Use

Var Name	Item	Response Options	Response
ccu01	Have you ever smoked a cigarette?	0. No<br>1. Yes	
ccu02	On what approximate day did you first smoke a cigarette?	*Date (mm dd yyyy)*	
ccu03	How many times in the past [time_point] did you smoke a cigarette?	0. Never	

CID:	Date: DD MMM YY	School Code:	Initials of Data Collector:

		1. Once a month or less<br>2. About once a week<br>3. Several times a week<br>4. About once a day<br>5. Several times a day	

4.5 Other tobacco products use

Var Name	Item	Response Options	Response
cotu01	Have you ever used other tobacco products, such as chewing tobacco, snuff, dip, snus, or dissolvable tobacco products, such as Copenhagen, Grizzly, Skoal, or Camel Snus (do not count any electronic vapor products)?	0. No<br>1. Yes	
cotu02	On what approximate day did you first use other tobacco products, such as chewing tobacco, snuff, dip, snus, or dissolvable tobacco products, such as Copenhagen, Grizzly, Skoal, or Camel Snus (do not count any electronic vapor products)?	*Date (mm dd yyyy)*	
cotu03	How many times in the past [time_point] did you use other tobacco products, such as chewing tobacco, snuff, dip, snus, or dissolvable tobacco products, such as Copenhagen, Grizzly, Skoal, or Camel Snus (do not count any electronic vapor products)?	0. Never<br>1. Once a month or less<br>2. About once a week<br>3. Several times a week<br>4. About once a day<br>5. Several times a day	

4.6 Cigarette Intentions

Var Name	Item	Response Options	Response
cci01	Do you plan to smoke cigarettes in the next 30 days?	0. No<br>1. Probably No<br>2. Probably Yes<br>3. Yes	
cci02	Do you plan to smoke cigarettes in the next [time_point]?	0. No<br>1. Probably No<br>2. Probably Yes<br>3. Yes	

CID:	Date: DD MMM YY	School Code:	Initials of Data Collector:

4.7 Affiliation with Cigarette-Smoking Peers

Var Name	Item	Response Options	Response
cacp01	How many of your friends do you think smoke cigarettes?	0. None<br>1. A few<br>2. Some<br>3. Most<br>4. All	

4.8 Peer Norms about Cigarettes

Var Name	Item	Response Options	Response
cipnc01	How do most of your friends feel about people your age smoking cigarettes?	1. Strongly disapprove<br>2. Disapprove<br>3. Somewhat disapprove<br>4. Somewhat approve<br>Approve<br>Strongly approve	
cdpnc01	How many of your friends do you estimate occasionally smoke cigarettes?	0. None<br>1. Very few<br>2. Some<br>3. Most<br>4. All	

CID:	Date: D D M M M Y Y	School Code:	Initials of Data Collector:

4.9 E-Cigarette Definition

Var Name	Item	Response Options	Response
cecdintro01	The following set of questions will be about **e-cigarettes**. By e-cigarettes we mean any electronic vapor product such as JUUL, Vuse, MarkTen, and blu. Electronic vapor products include e-cigarettes, vapes, vape pens, e-cigars, ehookahs, hookah pens, and mods. 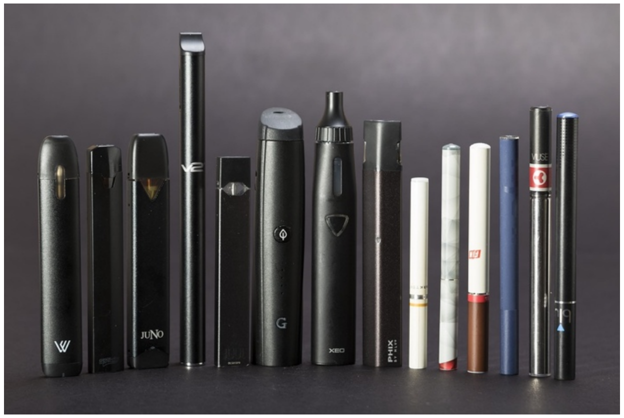		

4.10 E-cigarette expectations

Var Name	Item	Response Options	Response
ceceintro	You do not need to have any experience using vaping devices to answer these questions, just tell us what you think.<br><br>Answer each statement based on YOUR own thoughts, feelings, and beliefs about vaping devices. We are interested in what you think about vaping devices, not what others might think.<br><br>Using the scale below, please indicate how much you agree or disagree with each of the following statements.		
cece01	I would worry about my health.	1. Strongly disagree	

CID:	Date: DD MMM YY	School Code:	Initials of Data Collector:

		2. Disagree<br>3. Somewhat disagree<br>4. Somewhat agree<br>5. Agree<br>6. Strongly agree	
cece02	I would be concerned about the risks.	1. Strongly disagree<br>2. Disagree<br>3. Somewhat disagree<br>4. Somewhat agree<br>5. Agree<br>6. Strongly agree	
cece03	I would harm my lungs.	1. Strongly disagree<br>2. Disagree<br>3. Somewhat disagree<br>4. Somewhat agree<br>5. Agree<br>6. Strongly agree	
cece04	I would damage my brain.	1. Strongly disagree<br>2. Disagree<br>3. Somewhat disagree<br>4. Somewhat agree<br>5. Agree<br>6. Strongly agree	
cece05	I would wonder what I was inhaling.	1. Strongly disagree<br>2. Disagree<br>3. Somewhat disagree<br>4. Somewhat agree<br>5. Agree<br>6. Strongly agree	
cece06	I would inhale harmful chemicals.	1. Strongly disagree<br>2. Disagree<br>3. Somewhat disagree<br>4. Somewhat agree<br>5. Agree<br>6. Strongly agree	
cece07	I would inhale nicotine.	1. Strongly disagree<br>2. Disagree	

		3. Somewhat disagree<br>4. Somewhat agree<br>5. Agree<br>6. Strongly agree	
cece08	I would get addicted.	1. Strongly disagree<br>2. Disagree<br>3. Somewhat disagree<br>4. Somewhat agree<br>5. Agree<br>6. Strongly agree	
cece09	I would get hooked on tobacco.	1. Strongly disagree<br>2. Disagree<br>3. Somewhat disagree<br>4. Somewhat agree<br>5. Agree<br>6. Strongly agree	
cece10	I would be unable to stop vaping when I want to.	1. Strongly disagree<br>2. Disagree<br>3. Somewhat disagree<br>4. Somewhat agree<br>5. Agree<br>6. Strongly agree	

4.11 E-cigarette willingness

Var Name	Item	Response Options	Response
cecwintro01	Suppose you were with some friends and one of them offered you an e-cigarette or vape/JUUL.		
cecw01	How likely is it that you would take it and try it?	1. Not at all likely<br>2. Unlikely<br>3. Somewhat unlikely<br>4. Somewhat likely<br>5. Likely<br>6. Very likely	
cecw02	How likely is it that you would tell them “no”?	1. Not at all likely<br>2. Unlikely<br>3. Somewhat unlikely<br>4. Somewhat likely	

CID:	Date: DD MMM YY	School Code:	Initials of Data Collector:

		5. Likely<br>6. Very likely	
cecw03	How likely is it that you would leave the situation?	1. Not at all likely<br>2. Unlikely<br>3. Somewhat unlikely<br>4. Somewhat likely<br>5. Likely<br>6. Very likely	

4.12 E-cigarette use

Var Name	Item	Response Options	Response
cecu01	Have you ever used an e-cigarette or vape/JUUL?	0. No<br>1. Yes	
cecu02	On what approximate day did you first use an e-cigarette or vape/JUUL?	*Date (mm dd yyyy):*	
cecu03	How many times in the past [time_point] did you use an e-cigarette or vape/JUUL?	0. Never<br>1. Once a month or less<br>2. About once a week<br>3. Several times a week<br>4. About once a day<br>5. Several times a day	

4.13 E-cigarette intentions

Var Name	Item	Response Options	Response
ceci01	Do you plan to use e-cigarettes or vapes/JUULs in the next 30 days?	0. No<br>1. Probably No<br>2. Probably Yes<br>3. Yes	
ceci02	Do you plan to use e-cigarettes or vapes/JUULs in the next [time_point]?	0. No<br>1. Probably No<br>2. Probably Yes<br>3. Yes	

CID:	Date: DD MMM YY	School Code:	Initials of Data Collector:

4.14 Affiliation with e-cigarette using peers

Var Name	Item	Response Options	Response
caecp01	How many of your friends do you think use e-cigarettes or vapes/JUULs?	0. None<br>1. A few<br>2. Some<br>3. Most<br>4. All	

4.15 Peer Norms about E-cigarettes

Var Name	Item	Response Options	Response
cipne01	How do most of your friends feel about people your age using e-cigarettes or vapes (like JUULs)?	1. Strongly disapprove<br>2. Disapprove<br>3. Somewhat disapprove<br>4. Somewhat approve<br>5. Approve<br>6. Strongly approve	
cdpne01	How many of your friends do you estimate occasionally use e-cigarettes or vapes (like JUULs)?	0. None<br>2. Very few<br>3. Some<br>4. Most<br>5. All	

CID:	Date: D D M M M Y Y	School Code:	Initials of Data Collector:

4.16 Alcohol Definition

Variable Name	Item	Response Options	Response
cadintro01	The following set of questions will be about alcohol. By alcohol we mean any alcoholic beverage such as, beer, wine, wine coolers, and liquor (whiskey, rum, vodka, or gin). We do not mean alcohol that people drink for religious reasons.		

4.17 Alcohol Expectancies (Short Alcohol Expectancy Scale)

Variable Name	Item	Response Options	Response
caesintro01	You do not need to have any experience drinking alcohol to answer these questions.<br><br>Answer each statement based on YOUR own thoughts, feelings, and beliefs about drinking alcohol. We are interested in what you think about drinking alcohol, not what others might think.<br><br>Using the scale below, please indicate how much you agree or disagree with each of the following statements.		
caes01	Most people become happy and feel good when they drink alcohol.	1. Strongly disagree<br>2. Disagree<br>3. Somewhat disagree<br>4. Somewhat agree<br>5. Agree<br>6. Strongly agree	
caes02	It is easier to open up and speak about one's feelings after drinking alcohol.	1. Strongly disagree<br>2. Disagree<br>3. Somewhat disagree<br>4. Somewhat agree<br>5. Agree<br>6. Strongly agree	
caes03	People will come up with new and exciting things more easily when they drink alcohol.	1. Strongly disagree<br>2. Disagree<br>3. Somewhat disagree<br>4. Somewhat agree<br>5. Agree<br>6. Strongly agree	

CID:	Date: DD MMM YY	School Code:	Initials of Data Collector:

caes04	It is easier to feel comfortable in being with others when one drinks alcohol.	1. Strongly disagree<br>2. Disagree<br>3. Somewhat disagree<br>4. Somewhat agree<br>5. Agree<br>6. Strongly agree	
caes05	People become more friendly when they have been drinking and are a little drunk.	1. Strongly disagree<br>2. Disagree<br>3. Somewhat disagree<br>4. Somewhat agree<br>5. Agree<br>6. Strongly agree	
caes06	When people drink alcohol, they are more likely to irritate others.	1. Strongly disagree<br>2. Disagree<br>3. Somewhat disagree<br>4. Somewhat agree<br>5. Agree<br>6. Strongly agree	
caes07	It is O.K. to drink alcohol because then one can join in with others who are having fun.	1. Strongly disagree<br>2. Disagree<br>3. Somewhat disagree<br>4. Somewhat agree<br>5. Agree<br>6. Strongly agree	
caes08	It is easier for people to say what they are really thinking after becoming drunk.	1. Strongly disagree<br>2. Disagree<br>3. Somewhat disagree<br>4. Somewhat agree<br>5. Agree<br>6. Strongly agree	
caes09	People who have been drinking feel like they get power to control others.	1. Strongly disagree<br>2. Disagree<br>3. Somewhat disagree<br>4. Somewhat agree<br>5. Agree<br>6. Strongly agree	
caes10	Many alcoholic drinks taste good.	1. Strongly disagree	

CID:	Date: DD MMM YY	School Code:	Initials of Data Collector:

		2. Disagree<br>3. Somewhat disagree<br>4. Somewhat agree<br>5. Agree<br>6. Strongly agree	
caes11	Alcohol helps people stand up against the pressure and bullying from others.	1. Strongly disagree<br>2. Disagree<br>3. Somewhat disagree<br>4. Somewhat agree<br>5. Agree<br>6. Strongly agree	
caes12	Annoyances and worries disappear when drinking alcohol.	1. Strongly disagree<br>2. Disagree<br>3. Somewhat disagree<br>4. Somewhat agree<br>5. Agree<br>6. Strongly agree	
caes13	Alcohol makes kissing and touching both easier and better.	1. Strongly disagree<br>2. Disagree<br>3. Somewhat disagree<br>4. Somewhat agree<br>5. Agree<br>6. Strongly agree	
caes14	Alcohol makes people more polite.	1. Strongly disagree<br>2. Disagree<br>3. Somewhat disagree<br>4. Somewhat agree<br>5. Agree<br>6. Strongly agree	
caes15	People become more friendly and less formal when they drink alcohol.	1. Strongly disagree<br>2. Disagree<br>3. Somewhat disagree<br>4. Somewhat agree<br>5. Agree<br>6. Strongly agree	
caes16	Alcohol makes people relax.	1. Strongly disagree<br>2. Disagree	

CID:	Date: DD MMM YY	School Code:	Initials of Data Collector:

		3. Somewhat disagree<br>4. Somewhat agree<br>5. Agree<br>6. Strongly agree	
caes17	When people are drunk, they are more likely to take advantage of others.	1. Strongly disagree<br>2. Disagree<br>3. Somewhat disagree<br>4. Somewhat agree<br>5. Agree<br>6. Strongly agree	
caes18	One is better able to feel in touch with others when drinking alcohol.	1. Strongly disagree<br>2. Disagree<br>3. Somewhat disagree<br>4. Somewhat agree<br>5. Agree<br>6. Strongly agree	
caes19	People don't become angry as easily when they are drunk.	1. Strongly disagree<br>2. Disagree<br>3. Somewhat disagree<br>4. Somewhat agree<br>5. Agree<br>6. Strongly agree	
caes20	Parties become more fun when alcoholic beverages are consumed there.	1. Strongly disagree<br>2. Disagree<br>3. Somewhat disagree<br>4. Somewhat agree<br>5. Agree<br>6. Strongly agree	
caes21	People don't feel so alone when they are drinking and becoming drunk.	1. Strongly disagree<br>2. Disagree<br>3. Somewhat disagree<br>4. Somewhat agree<br>5. Agree<br>6. Strongly agree	
caes22	People can better control their moods when they are drunk.	1. Strongly disagree<br>2. Disagree<br>3. Somewhat disagree	

CID:	Date: DD MMM YY	School Code:	Initials of Data Collector:

		4. Somewhat agree<br>5. Agree<br>6. Strongly agree	
caes23	One doesn't have to think about mistakes one has made when one is drunk.	1. Strongly disagree<br>2. Disagree<br>3. Somewhat disagree<br>4. Somewhat agree<br>5. Agree<br>6. Strongly agree	
caes24	It is easier to speak in front of a group of people when one is drunk.	1. Strongly disagree<br>2. Disagree<br>3.Somewhat disagree<br>4. Somewhat agree<br>5. Agree<br>6. Strongly agree	
caes25	People get into better moods when they are drunk.	1. Strongly disagree<br>2. Disagree<br>3.Somewhat disagree<br>4.Somewhat agree<br>5. Agree<br>6.Strongly agree	
caes26	Students who sometimes drink alcohol don't get their homework done as well as others.	1. Strongly disagree<br>2. Disagree<br>3. Somewhat disagree<br>4. Somewhat agree<br>5. Agree<br>6. Strongly agree	
caes27	One becomes better able to flirt with a person one is attracted to when one is drunk.	1. Strongly disagree<br>2. Disagree<br>3. Somewhat disagree<br>4. Somewhat agree<br>5. Agree<br>6. Strongly agree	

4.18 Alcohol Willingness

CID:	Date: D D M M M Y Y	School Code:	Initials of Data Collector:

Var Name	Item	Response Options	Response
cawintro01	Suppose you were with some friends and one of them offered you alcohol.		
caw01	How likely is it that you would take it and try it?	1. Not at all likely<br>2. Unlikely<br>3. Somewhat unlikely<br>4. Somewhat likely<br>5. Likely<br>6. Very likely	
caw02	How likely is it that you would tell them "no"?	1. Not at all likely<br>2. Unlikely<br>3. Somewhat unlikely<br>4. Somewhat likely<br>5. Likely<br>6. Very likely	
caw03	How likely is it that you would leave the situation?	1. Not at all likely<br>2. Unlikely<br>3. Somewhat unlikely<br>4. Somewhat likely<br>5. Likely<br>6. Very likely	

4.19 Alcohol Use

Var Name	Item	Response Options	Response
cau01	Have you **ever**, even once, had a drink of any type of alcoholic beverage? Please do not include times when you only had a sip or two from a drink.	0. No<br>1. Yes	
cau02	On what approximate day did you first drink alcohol? Please do not include the time when you only had a sip or two from a drink.	*Date (mm dd yyyy)*	
cau03	How many times in the past [time_point] did you have a drink of alcohol? Please do not include times when you only had a sip or two from a drink.	0. Never<br>1. Once a month or less<br>2. About once a week	

CID:	Date: DD MMM YY	School Code:	Initials of Data Collector:

		3. Several times a week<br>4. About once a day<br>5. Several times a day	

4.20 Alcohol Intentions

Var Name	Item	Response Options	Response
cai01	Do you plan to drink alcohol in the next 30 days?	0. No<br>1. Probably No<br>2. Probably Yes<br>3. Yes	
cai02	Do you plan to drink alcohol in the next [time_point]?	0. No<br>1. Probably No<br>2. Probably Yes<br>3. Yes	

4.21 Affiliation with Alcohol drinking peers

Var Name	Item	Response Options	Response
caap01	How many of your friends do you think drink alcoholic beverages (for non-religious purposes)?	0. None<br>1. A few<br>2. Some<br>3. Most<br>4. All	

4.22 Peer Norms about Alcohol

Var Name	Item	Response Options	Response

CID:	Date: D D M M M Y Y	School Code:	Initials of Data Collector:

cipna01	How do most of your friends feel about people your age drinking alcohol?	1. Strongly disapprove<br>2. Disapprove<br>3. Somewhat disapprove<br>4. Somewhat approve<br>5. Approve<br>Strongly approve	
cipna02	How do most of your friends feel about people your age getting drunk?	1. Strongly disapprove<br>2. Disapprove<br>3. Somewhat disapprove<br>4. Somewhat approve<br>5. Approve<br>6. Strongly approve	
cdpna01	How many of your friends do you estimate occasionally drink alcohol?	0. None<br>2. Very few<br>3. Some<br>4. Most<br>5. All	

CID:	Date: D D M M M Y Y	School Code:	Initials of Data Collector:

4.23 Marijuana Definition

Var Name	Item	Response Options	Response
cmdintro01	The following set of questions will be about marijuana. By marijuana we also mean pot, weed, or cannabis that you may have smoked, vaped, or eaten.<br>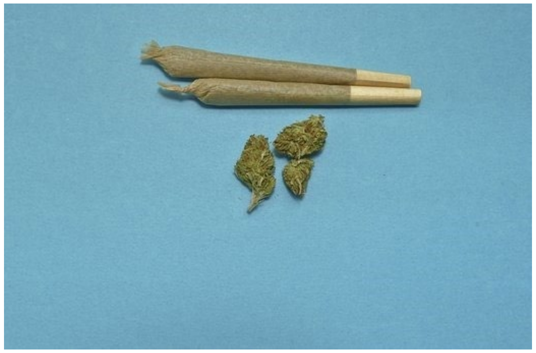		

4.24 Marijuana Expectancies (Marijuana Effect Expectancy Questionnaire – Brief)

Var Name	Item	Response Options	Response
cmeeqintro	You do not need to have any experience using marijuana to answer these questions.<br><br>Answer each statement based on YOUR own thoughts, feelings, and beliefs about using marijuana. We are interested in what you think about using marijuana, not what others might think.<br><br>Using the scale below, please indicate how much you agree or disagree with each of the following statements. Answer with what you believe happens when people use a moderate amount of marijuana (whatever moderate means to you).		

CID:	Date: DD MMM YY	School Code:	Initials of Data Collector:

cmeeq01	I get a sense of relaxation from using marijuana.	1. Strongly disagree<br>2. Disagree<br>3. Somewhat disagree<br>4. Somewhat agree<br>5. Agree<br>6. Strongly agree	
cmeeq02	Using marijuana makes me less tense or relieves anxiety; it helps me to unwind.	1. Strongly disagree<br>2. Disagree<br>3. Somewhat disagree<br>4. Somewhat agree<br>5. Agree<br>6. Strongly agree	
cmeeq03	Marijuana makes me carefree and I do not care about my problems as much.	1. Strongly disagree<br>2. Disagree<br>3. Somewhat disagree<br>4. Somewhat agree<br>5. Agree<br>6. Strongly agree	
cmeeq04	I am not concerned about how others evaluate me when I am on marijuana.	1. Strongly disagree<br>2. Disagree<br>3. Somewhat disagree<br>4. Somewhat agree<br>5. Agree<br>6. Strongly agree	
cmeeq05	When I use marijuana, I do not feel insecure.	1. Strongly disagree<br>2. Disagree<br>3. Somewhat disagree<br>4. Somewhat agree<br>5. Agree<br>6. Strongly agree	
cmeeq06	Marijuana makes me say things I do not mean.	1. Strongly disagree<br>2. Disagree<br>3. Somewhat disagree<br>4. Somewhat agree<br>5. Agree<br>6. Strongly agree	

CID:	Date: DD MMM YY	School Code:	Initials of Data Collector:

cmeeq07	If I have been using marijuana, it is harder for me to concentrate and understand the meaning of what is being said.	1. Strongly disagree<br>2. Disagree<br>3. Somewhat disagree<br>4. Somewhat agree<br>5. Agree<br>6. Strongly agree	
cmeeq08	Marijuana slows thinking and actions.	1. Strongly disagree<br>2. Disagree<br>3. Somewhat disagree<br>4. Somewhat agree<br>5. Agree<br>6. Strongly agree	
cmeeq09	If I have been using marijuana it is harder to remember things.	1. Strongly disagree<br>2. Disagree<br>3. Somewhat disagree<br>4. Somewhat agree<br>5. Agree<br>6. Strongly agree	
cmeeq10	Things seem unreal and I feel out of touch with what is going on around me when I use marijuana.	1. Strongly disagree<br>2. Disagree<br>3. Somewhat disagree<br>4. Somewhat agree<br>5. Agree<br>6. Strongly agree	
cmeeq11	When I use marijuana, I feel like I have heavy feet and no coordination.	1. Strongly disagree<br>2. Disagree<br>3. Somewhat disagree<br>4. Somewhat agree<br>5. Agree<br>6. Strongly agree	
cmeeq12	Marijuana tastes and smells bad.	1. Strongly disagree<br>2. Disagree<br>3. Somewhat disagree<br>4. Somewhat agree<br>5. Agree<br>6. Strongly agree	
cmeeq13	Marijuana causes me to lose control and become careless.	1. Strongly disagree	

CID:	Date: DD MMM YY	School Code:	Initials of Data Collector:

		2. Disagree<br>3. Somewhat disagree<br>4. Somewhat agree<br>5. Agree<br>6. Strongly agree	
cmeeq14	Marijuana makes it easier to escape from problems and responsibilities.	1. Strongly disagree<br>2. Disagree<br>3. Somewhat disagree<br>4. Somewhat agree<br>5. Agree<br>6. Strongly agree	
cmeeq15	Marijuana can cause me to become depressed and disappointed with myself.	1. Strongly disagree<br>2. Disagree<br>3. Somewhat disagree<br>4. Somewhat agree<br>5. Agree<br>6. Strongly agree	
cmeeq16	Marijuana can make my feelings change from happy to sad.	1. Strongly disagree<br>2. Disagree<br>3. Somewhat disagree<br>4. Somewhat agree<br>5. Agree<br>6. Strongly agree	
cmeeq17	After using marijuana my eyelids become heavy and I become drowsy.	1. Strongly disagree<br>2. Disagree<br>3. Somewhat disagree<br>4. Somewhat agree<br>5. Agree<br>6. Strongly agree	
cmeeq18	Marijuana can make me angry and possibly violent.	1. Strongly disagree<br>2. Disagree<br>3. Somewhat disagree<br>4. Somewhat agree<br>5. Agree<br>6. Strongly agree	
cmeeq19	After the “high” of using marijuana, I feel down.	1. Strongly disagree<br>2. Disagree	

CID:	Date: DD MMM YY	School Code:	Initials of Data Collector:

		3. Somewhat disagree<br>4. Somewhat agree<br>5. Agree<br>6. Strongly agree	
cmeeq20	Marijuana does not alter my personality.	1. Strongly disagree<br>2. Disagree<br>3. Somewhat disagree<br>4. Somewhat agree<br>5. Agree<br>6. Strongly agree	
cmeeq21	Marijuana makes me critical and short-tempered.	1. Strongly disagree<br>2. Disagree<br>3. Somewhat disagree<br>4. Somewhat agree<br>5. Agree<br>6. Strongly agree	
cmeeq22	It is difficult for me to express my thoughts clearly if I have been using marijuana.	1. Strongly disagree<br>2. Disagree<br>3. Somewhat disagree<br>4. Somewhat agree<br>5. Agree<br>6. Strongly agree	
cmeeq23	Marijuana makes me calm.	1. Strongly disagree<br>2. Disagree<br>3. Somewhat disagree<br>4. Somewhat agree<br>5. Agree<br>6. Strongly agree	
cmeeq24	Marijuana changes my perception of time and distance.	1. Strongly disagree<br>2. Disagree<br>3. Somewhat disagree<br>4. Somewhat agree<br>5. Agree<br>6. Strongly agree	
cmeeq25	I am more relaxed in social situations if I've been using marijuana.	1. Strongly disagree<br>2. Disagree<br>3. Somewhat disagree	

CID:	Date: DD MMM YY	School Code:	Initials of Data Collector:

		4. Somewhat agree<br>5. Agree<br>6. Strongly agree	
cmeeq26	Marijuana makes reaction times slower.	1. Strongly disagree<br>2. Disagree<br>3. Somewhat disagree<br>4. Somewhat agree<br>5. Agree<br>6. Strongly agree	
cmeeq27	Using marijuana is similar to being "high" from drinking alcohol.	1. Strongly disagree<br>2. Disagree<br>3. Somewhat disagree<br>4. Somewhat agree<br>5. Agree<br>6. Strongly agree	

4.25 Marijuana Willingness

Var Name	Item	Response Options	Response
cmwintro	Suppose you were with some friends and one of them offered you marijuana.		
cmw01	How likely is it that you would take it and try it?	1. Not at all likely<br>2. Unlikely<br>3. Somewhat unlikely<br>4. Somewhat likely<br>5. Likely<br>6. Very likely	
cmw02	How likely is it that you would tell them "no"?	1. Not at all likely<br>2. Unlikely<br>3. Somewhat unlikely<br>4. Somewhat likely<br>5. Likely<br>6. Very likely	
cmw03	How likely is it that you would leave the situation?	1. Not at all likely<br>2. Unlikely<br>3. Somewhat unlikely<br>4. Somewhat likely	

CID:	Date: DD MMM YY	School Code:	Initials of Data Collector:

		5. Likely<br>6. Very likely	

4.26 Marijuana Use

Var Name	Item	Response Options	Response
cmu01	Have you ever used marijuana?	0. No<br>1. Yes	
cmu02	On what approximate day did you first use marijuana?	*Date (mm dd yyyy)*	
cmu03	How many times in the past [time_point] did you use marijuana?	0. Never<br>1. Once a month or less<br>2. About once a week<br>3. Several times a week<br>4. About once a day<br>5. Several times a day	

4.27 Marijuana Intentions

Var Name	Item	Response Options	Response
cmi01	Do you plan to use marijuana in the next 30 days?	0. No<br>1. Probably No<br>2. Probably Yes<br>3. Yes	
cmi02	Do you plan to use marijuana in the next [time_point]?	0. No<br>1. Probably No<br>2. Probably Yes<br>3. Yes	

4.28 Affiliation with Marijuana using peers

Var Name	Item	Response Options	Response
camp01	How many of your friends do you think use marijuana (pot, weed, grass) or hashish?	0. None<br>1. A few<br>2. Some	

CID:	Date: DD MMM YY	School Code:	Initials of Data Collector:

		3. Most<br>4. All	

4.29 Peer Norms about Marijuana

Var Name	Item	Response Options	Response
cipnm01	How do most of your friends feel about people your age using marijuana?	1. Strongly disapprove<br>2. Disapprove<br>3. Somewhat disapprove<br>4. Somewhat approve<br>5. Approve<br>Strongly approve	
cdpnm01	How many of your friends do you estimate occasionally use marijuana?	0. None<br>2. Very few<br>3. Some<br>4. Most<br>5. All	

CID:	Date: DD MMM YY	School Code:	Initials of Data Collector:

4.30 Other Drugs Definition

Var Name	Item	Response Options	Response
coddintro01	The following set of questions will be about **other drugs**. By other drugs we mean prescription drugs used for non-medical reasons, inhalants, and other substances used to get high, which can include cocaine (including powder, crack, or freebase), steroids taken without a doctor's prescription), or any other substance that can be used to get high.<br><br>For this section, "other drugs" **does not include** alcohol, tobacco, e-cigarettes, or marijuana.		

4.31 Other Drug expectancies

Var Name	Item	Response Options	Response
codeintro01	You do not need to have any experience using other drugs to answer these questions.<br><br>Answer each statement based on YOUR own thoughts, feelings, and beliefs about using other drugs. We are interested in what you think about using other drugs, not what others might think.<br><br>Using the scale below, please indicate how much you agree or disagree with each of the following statements. Answer with what you believe happens when people use a moderate amount of other drugs (whatever moderate means to you).		
code01	Other drugs make me talk more than usual.	1. Strongly disagree<br>2. Disagree<br>3.Somewhat disagree<br>4.Somewhat agree<br>5. Agree<br>6. Strongly agree	
code02	Other drugs make me say things I do not mean.	1. Strongly disagree<br>2. Disagree<br>3.Somewhat disagree<br>4.Somewhat agree<br>5. Agree<br>6. Strongly agree	

CID:	Date: DD MMM YY	School Code:	Initials of Data Collector:

code03	I am more sociable when I use other drugs.	1. Strongly disagree<br>2. Disagree<br>3.Somewhat disagree<br>4.Somewhat agree<br>5. Agree<br>6. Strongly agree	
code04	Using other drugs makes me feel like part of the group.	1. Strongly disagree<br>2. Disagree<br>3. Somewhat disagree<br>4. Somewhat agree<br>5. Agree<br>6. Strongly agree	
code05	Other drugs taste and smell bad.	1. Strongly disagree<br>2. Disagree<br>3. Somewhat disagree<br>4. Somewhat agree<br>5. Agree<br>6. Strongly agree	
code06	I have a happy, good feeling when I use other drugs.	1. Strongly disagree<br>2. Disagree<br>3. Somewhat disagree<br>4. Somewhat agree<br>5. Agree<br>6. Strongly agree	
code07	Using other drugs causes me to lose control and become careless.	1. Strongly disagree<br>2. Disagree<br>3. Somewhat disagree<br>4. Somewhat agree<br>5. Agree<br>6. Strongly agree	
code08	I am less motivated when I use other drugs.	1. Strongly disagree<br>2. Disagree<br>3. Somewhat disagree<br>4. Somewhat agree<br>5. Agree<br>6. Strongly agree	
code09	Other drugs can cause me to become depressed and disappointed with myself.	1. Strongly disagree	

CID:	Date: DD MMM YY	School Code:	Initials of Data Collector:

		2. Disagree<br>3. Somewhat disagree<br>4. Somewhat agree<br>5. Agree<br>6. Strongly agree	
code10	Other drugs can make my feelings change from happy to sad.	1. Strongly disagree<br>2. Disagree<br>3. Somewhat disagree<br>4. Somewhat agree<br>5. Agree<br>6. Strongly agree	
code11	I act excited when I use other drugs.	1. Strongly disagree<br>2. Disagree<br>3. Somewhat disagree<br>4. Somewhat agree<br>5. Agree<br>6. Strongly agree	
code12	Other drugs do not make me feel more romantic or attracted to other people.	1. Strongly disagree<br>2. Disagree<br>3. Somewhat disagree<br>4. Somewhat agree<br>5. Agree<br>6. Strongly agree	
code13	Other drugs can make me angry and possibly violent.	1. Strongly disagree<br>2. Disagree<br>3. Somewhat disagree<br>4. Somewhat agree<br>5. Agree<br>6. Strongly agree	
code14	After the “high” of using other drugs, I feel down.	1. Strongly disagree<br>2. Disagree<br>3. Somewhat disagree<br>4. Somewhat agree<br>5. Agree<br>6. Strongly agree	
code15	Other drugs make me critical and short-tempered.	1. Strongly disagree<br>2. Disagree	

CID:	Date: DD MMM YY	School Code:	Initials of Data Collector:

		3. Somewhat disagree<br>4. Somewhat agree<br>5. Agree<br>6. Strongly agree	
code16	I have a better time at parties if I am using other drugs.	1. Strongly disagree<br>2. Disagree<br>3. Somewhat disagree<br>4. Somewhat agree<br>5. Agree<br>6. Strongly agree	
code17	I am more willing to do things that I normally would not do when I use other drugs.	1. Strongly disagree<br>2. Disagree<br>3. Somewhat disagree<br>4. Somewhat agree<br>5. Agree<br>6. Strongly agree	
code18	Using other drugs is similar to being “high” from drinking alcohol.	1. Strongly disagree<br>2. Disagree<br>3. Somewhat disagree<br>4. Somewhat agree<br>5. Agree<br>6. Strongly agree	

4.32 Other drugs willingness

Var Name	Item	Response Options	Response
codwintro01	Suppose you were with some friends and one of them offered you other drugs (other than alcohol, marijuana, cigarettes, and e-cigarettes).		
codw01	How likely is it that you would take it and try it?	1. Not at all likely<br>2. Unlikely<br>3. Somewhat unlikely<br>4. Somewhat likely<br>5. Likely<br>6. Very likely	
codw02	How likely is it that you would tell them “no”?	1. Not at all likely<br>2. Unlikely<br>3. Somewhat unlikely	

CID:	Date: DD MMM YY	School Code:	Initials of Data Collector:

		4. Somewhat likely<br>5. Likely<br>6. Very likely	
codw03	How likely is it that you would leave the situation?	1. Not at all likely<br>2. Unlikely<br>3. Somewhat unlikely<br>4. Somewhat likely<br>5. Likely<br>6. Very likely	

4.33 Other drug use

Var Name	Item	Response Options	Response
codu01	Have you ever used other drugs (not including alcohol, marijuana, cigarettes, and e-cigarettes)?	0. No<br>1. Yes	
codu02	On what approximate day did you first use other drugs (not including alcohol, marijuana, cigarettes, and e-cigarettes).?	*Date (mm dd yyyy)*	
codu03	How many times in the past [time_point] did you use other drugs (not including alcohol, marijuana, cigarettes, and e-cigarettes)?	0. Never<br>1. Once a month or less<br>2. About once a week<br>3. Several times a week<br>4. About once a day<br>5. Several times a day	

4.34 Other Drug Intentions

Var Name	Item	Response Options	Response
codi01	Do you plan to use other drugs (not including alcohol, marijuana, cigarettes and e-cigarettes) in the next 30 days?	0. No<br>1. Probably No<br>2. Probably Yes<br>3. Yes	
codi02	Do you plan to use other drugs (not including alcohol, marijuana, cigarettes and e-cigarettes) in the next [time_point]?	0. No<br>1. Probably No<br>2. Probably Yes<br>3. Yes	

CID:	Date: DD MMM YY	School Code:	Initials of Data Collector:

codi03	Which other drugs (not including alcohol, marijuana, cigarettes and e-cigarettes) do you plan to use? (Check all that apply)	1. Inhalants<br>2. Prescription drugs (used for non-medical reasons)<br>3. Anabolic steroids (Juice, Roids)<br>5. Bath salts (Bloom, Cloud nine, Vanilla sky, White lightning)<br>6. Cocaine (Blow, Bump, C, Charlie, Coca, Coke, Flake, Rock, Snow, Toot)<br>7. MDMA (Ecstasy or Molly)<br>8. Methamphetamine (Meth)<br>9. Other	
codi03_spec	Please specify which other drugs you plan to use:		

4.35 Affiliation with other drug using peers

Var Name	Item	Response Options	Response
caodp01	How many of your friends do you think use other drugs (not including alcohol, marijuana, cigarettes, and e-cigarettes)?	0. None<br>1. A few<br>2. Some<br>3. Most<br>4. All	

4.36 Peer Norms about other drugs

Var Name	Item	Response Options	Response
cipnod01	How do most of your friends feel about people your age using other drugs (not including alcohol, marijuana, cigarettes, and e-cigarettes)?	1. Strongly disapprove<br>2. Disapprove<br>3. Somewhat disapprove<br>4. Somewhat approve<br>5. Approve<br>6. Strongly approve	
cdpnod01	How many of your friends do you estimate occasionally use other drugs (not including alcohol, marijuana, cigarettes, and e-cigarettes)?	0. None<br>2. Very few<br>3. Some<br>4. Most<br>5. All	

CID:	Date: D D M M M Y Y	School Code:	Initials of Data Collector:

4.37 Parental Attitudes Towards Use

Var Name	Item	Response Options	Response
cpatuintro01	How wrong does your parent in the study feel it would be for you to…		
cpatu01	Drink beer, wine or hard liquor (for example, vodka, whisky or gin) regularly (at least once or twice a month)?	1. Very wrong<br>2. Wrong<br>3. A little bit wrong<br>4. Not wrong at all	
cpatu02	Smoke cigarettes?	1. Very wrong<br>2. Wrong<br>3. A little bit wrong<br>4. Not wrong at all	
cpatu03	Use e-cigarettes or vapes/JUULs?	1. Very wrong<br>2. Wrong<br>3. A little bit wrong<br>4. Not wrong at all	
cpatu04	Use marijuana?	1. Very wrong<br>2. Wrong<br>3. A little bit wrong<br>4. Not wrong at all	
cpatu05	Use other drugs?	1. Very wrong<br>2. Wrong<br>3. A little bit wrong<br>4. Not wrong at all	

Section 5: HOW YOU FEEL ABOUT YOURSELF
5.1 Self Esteem

Variable Name	Question	Response Options	Response
crosenbergintro	The next section is asking about how you feel about yourself.<br><br>How much do you agree or disagree with each of the following statements?		
crosenberg01	I feel that I am a person of worth, at least on an equal plane with others.	1. Strongly disagree<br>2. Disagree<br>3. Somewhat disagree	

CID:	Date: DD MMM YY	School Code:	Initials of Data Collector:

		4. Somewhat agree<br>5. Agree<br>6. Strongly agree	
crosenberg02	I feel that I have a number of good qualities.	1. Strongly disagree<br>2. Disagree<br>3. Somewhat disagree<br>4. Somewhat agree<br>5. Agree<br>6. Strongly agree	
crosenberg03	All in all, I am inclined to feel that I am a failure.	1. Strongly disagree<br>2. Disagree<br>3. Somewhat disagree<br>4. Somewhat agree<br>5. Agree<br>6. Strongly agree	
crosenberg04	I am able to do things as well as most other people.	1. Strongly disagree<br>2. Disagree<br>3. Somewhat disagree<br>4. Somewhat agree<br>5. Agree<br>6. Strongly agree	
crosenberg05	I feel I do not have much to be proud of.	1. Strongly disagree<br>2. Disagree<br>3. Somewhat disagree<br>4. Somewhat agree<br>5. Agree<br>6. Strongly agree	
crosenberg06	I take a positive attitude toward myself.	1. Strongly disagree<br>2. Disagree<br>3. Somewhat disagree<br>4. Somewhat agree<br>5. Agree<br>6. Strongly agree	
crosenberg07	On the whole, I am satisfied with myself.	1. Strongly disagree<br>2. Disagree<br>3. Somewhat disagree<br>4. Somewhat agree	

CID:	Date: DD MMM YY	School Code:	Initials of Data Collector:

		5. Agree<br>6. Strongly agree	
crosenberg08	I wish I could have more respect for myself.	1. Strongly disagree<br>2. Disagree<br>3. Somewhat disagree<br>4. Somewhat agree<br>5. Agree<br>6. Strongly agree	
crosenberg09	I certainly feel useless at times.	1. Strongly disagree<br>2. Disagree<br>3. Somewhat disagree<br>4. Somewhat agree<br>5. Agree<br>6. Strongly agree	
crosenberg10	At times I think I am no good at all.	1. Strongly disagree<br>2. Disagree<br>3. Somewhat disagree<br>4. Somewhat agree<br>5. Agree<br>6. Strongly agree	

5.2 Mental Health

Variable Name	Question	Response Options	Response
cpscintro01	Please say how often each of the following has been true for you in the past [time_point]:		
cpsc01	Fidgety, unable to sit still.	0. Never<br>1. Sometimes<br>2. Often	
cpsc02	Feel sad, unhappy.	0. Never<br>1. Sometimes<br>2. Often	
cpsc03	Daydream too much.	0. Never<br>1. Sometimes<br>2. Often	
cpsc04	Refuse to share.	0. Never	

CID:	Date: DD MMM YY	School Code:	Initials of Data Collector:

		1. Sometimes<br>2. Often	
cpsc05	Do not understand other people's feelings.	0. Never<br>1. Sometimes<br>2. Often	
cpsc06	Feel hopeless.	0. Never<br>1. Sometimes<br>2. Often	
cpsc07	Have trouble concentrating.	0. Never<br>1. Sometimes<br>2. Often	
cpsc08	Fight with other people your age.	0. Never<br>1. Sometimes<br>2. Often	
cpsc09	Are down on yourself.	0. Never<br>1. Sometimes<br>2. Often	
cpsc10	Blame others for your troubles.	0. Never<br>1. Sometimes<br>2. Often	
cpsc11	Seem to be having less fun.	0. Never<br>1. Sometimes<br>2. Often	
cpsc12	Do not listen to rules.	0. Never<br>1. Sometimes<br>2. Often	
cpsc13	Act as if driven by a motor.	0. Never<br>1. Sometimes<br>2. Often	
cpsc14	Tease others.	0. Never<br>1. Sometimes<br>2. Often	
cpsc15	Worry a lot.	0. Never<br>1. Sometimes<br>2. Often	
cpsc16	Take things that do not belong to you.	0. Never	

CID:	Date: D D M M M Y Y	School Code:	Initials of Data Collector:

		1. Sometimes<br>2. Often	
cpsc17	Distract easily.	0. Never<br>1. Sometimes<br>2. Often	

Section 6: YOUR HOME & NEIGHBORHOOD

6.1 Family Meal Descriptive

Var Name	Item	Response Options	Response
cfddintro01	The following questions will ask you about <u>family meals</u>. In this study, we define <u>family meals</u> as the time when you join your parent/guardian (the one in the study with you) for a meal and one of you is eating, regardless of the type of food that is served. It is still considered a family meal if your parent/guardian is present while you eat but they do not eat the meal themselves (and vice versa).		
cfdd01	The following question is about family breakfasts. We define breakfast as your first meal of the day.<br><br>During a typical week in the <u>past [time_point]</u>, how many breakfasts do you usually eat with your parent?	0. 0 breakfasts<br>1. 1 breakfast<br>2. 2 breakfasts<br>3. 3 breakfasts<br>4. 4 breakfasts<br>5. 5 breakfasts<br>6. 6 breakfasts<br>7. 7 breakfasts	
cfdd02	The following question is about family lunch. We define lunch as the meal you eat in the middle of the day, between breakfast and dinner.<br><br>During a typical week in the <u>past [time_point]</u>, how many <u>lunches</u> do you usually eat with your parent?	0. 0 lunches<br>1. 1 lunch<br>2. 2 lunches<br>3. 3 lunches<br>4. 4 lunches<br>5. 5 lunches<br>6. 6 lunches<br>7. 7 lunches	
cfdd03	The following questions will ask you about family dinners. We define dinner as the meal you eat at the end of the day.	0. 0 dinners<br>1. 1 dinner	

CID:	Date: DD MMM YY	School Code:	Initials of Data Collector:

	During a typical week in the past [time_point], how many dinners do you usually eat with your parent?	2. 2 dinners<br>3. 3 dinners<br>4. 4 dinners<br>5. 5 dinners<br>6. 6 dinners<br>7. 7 dinners	
cfdd04	During a typical week in the past [time_point], about how many minutes do family dinners usually last (do not include the time it makes to make the meal or clean up)?	0. We never have family dinners<br>1. 0 - 15 minutes<br>2. 16 - 30 minutes<br>3. 31 - 45 minutes<br>4. 46 - 60 minutes<br>5. More than 60 minutes	
cfdd05	During a typical week in the past [time_point], how many people are usually at family dinners?	0. We never have family dinners<br>1. 2 people<br>2. 3 people<br>3. 4 people<br>4. 5 people<br>5. 6 people<br>6. 7 people<br>7. 8 people<br>8. 9 people<br>9. 10 or more people	
cfddintro03	For these questions, think about any of your parents or guardians. The answers can be same as the answers above, or they may be different.		
cfdd06	During a typical week in the past [time_point], did you eat family dinner with any OTHER parent/guardian?	0. No<br>1. Yes	
cfdd07	During a typical week in the past [time_point], how many family dinners did you eat with any OTHER parent/guardian?	0. 0 dinners<br>1. 1 dinner<br>2. 2 dinners<br>3. 3 dinners<br>4. 4 dinners<br>5. 5 dinners	

CID:	Date: DD MMM YY	School Code:	Initials of Data Collector:

		6. 6 dinners<br>7. 7 dinners	
cfdd08	During a typical week in the past [time_point], about how many minutes did family dinners with any OTHER parent/guardian usually last (do not include the time it takes to make the meal or clean it up)?	0. We never have family dinners<br>1. 0 - 15 minutes<br>2. 16 - 30 minutes<br>3. 31 - 45 minutes<br>4. 46 - 60 minutes<br>5. More than 60 minutes	

6.2 Family Dinner Index

Var Name	Item	Response Options	Response
cfdiintro	When we say family dinner, we mean the last meal of the day, as long as you are with your parent/guardian, even if you are not eating. If you do not eat family dinners frequently, think about the meal you eat most frequently with you parent.<br><br>During a typical week in the past [time_point]…		
cfdi01	How often are you supposed to be at dinners with your family?	0. Never<br>1. Rarely<br>2. Sometimes<br>3. Often<br>4. Always	
cfdi02	How much do you like being with your parent/guardian during family dinners?	0. Not at all<br>1. A little<br>2. Somewhat<br>3. A lot<br>4. Very much	
cfdi03	How much do you think your parent(s) enjoys family dinners in general (note: this does not include the food that is served)?	0. Not at all<br>1. A little<br>2. Somewhat<br>3. A lot	

CID:	Date: DD MMM YY	School Code:	Initials of Data Collector:

		4. Very much<br>5.	
cfdi04	In general, how much do people talk <u>to each other</u> during family dinners?	0. Not at all<br>1. A little<br>2. Somewhat<br>3. A lot<br>4. Very much<br>6.	
cfdi05	How often are people allowed to talk, send messages, or watch something during family dinner using personal devices (for example, phones)?	0. Never<br>1. Rarely<br>2. Sometimes<br>3. Often<br>4. Always	
cfdi06	How much do you participate in the conversation during family dinners?	0. Not at all<br>1. A little<br>2. Somewhat<br>3. A lot<br>4. Very much	
cfdi07	Of the foods served at family dinners, I am able to choose <u>how much</u> I eat.	1. Strongly disagree<br>2. Disagree<br>3. Not sure<br>4. Agree<br>5. Strong agree	
cfdi08	Of the foods served at family dinners, I am able to choose <u>the ones</u> I eat.	1. Strongly disagree<br>2. Disagree<br>3. Not sure<br>4. Agree<br>5. Strong agree	
cfdi09	In thinking about family dinners <u>in the past [time_point]</u>,that you eat in a home, either in your home or in someone else's home, please answer the following question.<br><br>How often do people watch shows, movies, or sports games during family dinners (<u>actively watching</u>, not just on in the background)?	0. Never<br>1. Rarely<br>2. Sometimes<br>3. Often<br>4. Always	

CID:	Date: DD MMM YY	School Code:	Initials of Data Collector:

6.3 Perceived Neighborhood Disorder

Var Name	Item	Response Options	Response
cpndintro01	We are going to be asking you some questions about your neighborhood.<br><br>How much do you agree or disagree with each of the following statements about the neighborhood you currently live in?		
cpnd01	There is a lot of graffiti in my neighborhood.	1. Strongly disagree<br>2. Disagree<br>3. Somewhat disagree<br>4. Somewhat agree<br>5. Agree<br>6. Strongly agree	
cpnd02	My neighborhood is noisy.	1. Strongly disagree<br>2. Disagree<br>3. Somewhat disagree<br>4. Somewhat agree<br>5. Agree<br>6. Strongly agree	
cpnd03	Vandalism is common in my neighborhood.	1. Strongly disagree<br>2. Disagree<br>3. Somewhat disagree<br>4. Somewhat agree<br>5. Agree<br>6. Strongly agree	
cpnd04	There are a lot of abandoned buildings in my neighborhood.	1. Strongly disagree<br>2. Disagree<br>3. Somewhat disagree<br>4. Somewhat agree<br>5. Agree<br>6. Strongly agree	
cpnd05	My neighborhood is clean.	1. Strongly disagree<br>2. Disagree<br>3. Somewhat disagree<br>4. Somewhat agree<br>5. Agree<br>6. Strongly agree	

CID:	Date: DD MMM YY	School Code:	Initials of Data Collector:

cpnd06	People in my neighborhood take good care of their houses and apartments.	1. Strongly disagree<br>2. Disagree<br>3. Somewhat disagree<br>4. Somewhat agree<br>5. Agree<br>6. Strongly agree	
cpnd07	There are too many people hanging around on the streets near my home.	1. Strongly disagree<br>2. Disagree<br>3. Somewhat disagree<br>4. Somewhat agree<br>5. Agree<br>6. Strongly agree	
cpnd08	There is too much drug use in my neighborhood.	1. Strongly disagree<br>2. Disagree<br>3. Somewhat disagree<br>4. Somewhat agree<br>5. Agree<br>6. Strongly agree	
cpnd09	There is too much alcohol use in my neighborhood.	1. Strongly disagree<br>2. Disagree<br>3. Somewhat disagree<br>4. Somewhat agree<br>5. Agree<br>6. Strongly agree	
cpnd10	I'm always having trouble with my neighbors.	1. Strongly disagree<br>2. Disagree<br>3. Somewhat disagree<br>4. Somewhat agree<br>5. Agree<br>6. Strongly agree	
cpnd11	There is a lot of crime in my neighborhood.	1. Strongly disagree<br>2. Disagree<br>3. Somewhat disagree<br>4. Somewhat agree<br>5. Agree<br>6. Strongly agree	

CID:	Date: DD MMM YY	School Code:	Initials of Data Collector:

cpnd12	In my neighborhood, people watch out for each other.	1. Strongly disagree<br>2. Disagree<br>3. Somewhat disagree<br>4. Somewhat agree<br>5. Agree<br>6. Strongly agree	
cpnd13	The police protection in my neighborhood is pretty good.	1. Strongly disagree<br>2. Disagree<br>3. Somewhat disagree<br>4. Somewhat agree<br>5. Agree<br>6. Strongly agree	
cpnd14	My neighborhood is safe.	1. Strongly disagree<br>2. Disagree<br>3. Somewhat disagree<br>4. Somewhat agree<br>5. Agree<br>6. Strongly agree	
cpnd15	I can trust most people in my neighborhood.	1. Strongly disagree<br>2. Disagree<br>3. Somewhat disagree<br>4. Somewhat agree<br>5. Agree<br>6. Strongly agree	

Section 7: ABOUT YOU

7.1 Social Desirability Scale

Var Name	Item	Response Options	Response
csdsintro	This questionnaire lists a number of experiences that most people your age have had at one time or another. Read each of these carefully.<br><br>After you have read one, decide whether it does or does not fit you. If it does, mark True; if it doesn't, mark False.		

CID:	Date: D D M M M Y Y	School Code:	Initials of Data Collector:

csds01	Have you ever felt like saying unkind things to a person?	0. False<br>1. True	
csds02	Are you always careful about keeping your clothing neat and your room picked up?	0. False<br>1. True	
csds03	Do you sometimes feel like staying home from school even if you are not sick?	0. False<br>1. True	
csds04	Do you ever say anything that makes somebody else feel bad?	0. False<br>1. True	
csds05	Are you always polite, even to people who are not very nice?	0. False<br>1. True	
csds06	Sometimes do you do things you've been told not to do?	0. False<br>1. True	
csds07	Do you always listen to your parents?	0. False<br>1. True	
csds08	Do you sometimes wish you could just play around instead of having to go to school?	0. False<br>1. True	
csds09	Have you ever broken a rule?	0. False<br>1. True	
csds10	Do you sometimes feel angry when you don't get your way?	0. False<br>1. True	
csds11	Do you sometimes feel like making fun of other people?	0. False<br>1. True	
csds12	Do you always do the right thing?	0. False<br>1. True	
csds13	Are there some times when you don't like to do what your parents tell you?	0. False<br>1. True	
csds14	Do you sometimes get mad when people don't do what you want them to do?	0. False<br>1. True	
