# Supplementary material for: Randomized controlled trial to test the efficacy of a brief, communication-based, substance use preventive intervention for parents of adolescents: Protocol for the SUPPER Project (Substance Use Prevention Promoted by Eating family meals Regularly)
Source: PLoS One. 2022 Feb 2;17(2):e0263016. doi: 10.1371/journal.pone.0263016 (PMC8809599; doi:10.1371/journal.pone.0263016)
Supplement: S4 File — (PDF) [file pone.0263016.s005.pdf]

PID:	Date: D D M M M Y Y	School Code:	Initials of Data Collector:

PID:	Date: DD MMM YY	School Code:	Initials of Data Collector:

Section 0: Introduction and Practice Questions

pintro			
	Thank you for taking the time to complete this survey. Your responses are very important to us. Please read each question carefully before answering.<br><br>Filling out this survey is your choice. You do not have to answer any questions unless you choose to. All the information you share with us will be kept private. It will not be seen by anyone, besides the people working on the study.<br><br>The survey is expected to take about **1 hour** to complete. Try to complete the entire survey in one sitting. If you need to stop and finish the survey at a later time, you can do so.<br><br>If you have any questions about the survey, please contact our study team at **(617) 636-3587** or e-mail us at thesupperproject@tufts.edu .<br><br>Throughout the survey, when we say "child" we mean the child that is in the study with you.<br><br>When taking this survey, you agree to:<br>1. Complete the survey yourself without assistance from anyone, except a study team member<br>2. Not share your responses with your child<br>3. Not watch or help your child complete their survey<br><br>**By clicking "continue" you agree to the above statements.**<br>Thank you for your help!		

**Var Name**	**Item**	**Response Options**	**Response**
cpracintro01	We are going to walk you through a few practice questions to make sure you feel comfortable using the iPad.<br><br>Throughout the survey, if you skip a question you will be asked whether you skipped the question on purpose or if it was a mistake. If it was a mistake, you will be able to answer the question. If you skipped the question because you did not want to answer it, you can leave it blank.		

PID:	Date: DD MMM YY	School Code:	Initials of Data Collector:

cprac01	Which is your favorite season?<br><br>If winter is your favorite season, you can choose the “Other” button and type your answer in the text box.	1. Spring<br>2. Summer<br>3. Fall<br>4. Other	
cprac01_spec	Please specify:		
cprac02	This is an example of a question where you can choose more than one answer. “Check all that apply” will tell you when you can select more than one answer.<br><br>Which of the following are shapes? (Check all that apply)	1. Triangle<br>2. Dog<br>3. Circle<br>4. Square	
cpracintro03	The next question is a practice grid question. It is important that you make a choice for each row before clicking on the “next” button.<br><br>How much do you agree with the following statements? (Select one answer for each line)		
cprac03	Winter is cold	1. Strongly disagree<br>2. Disagree<br>3. Somewhat disagree<br>4. Somewhat agree<br>5. Agree<br>6. Strongly agree	
cprac04	Dancing is fun	1. Strongly disagree<br>2. Disagree<br>3. Somewhat disagree<br>4. Somewhat agree<br>5. Agree<br>6. Strongly agree	
cprac05	Pizza is delicious	1. Strongly disagree<br>2. Disagree<br>3. Somewhat disagree	

PID:	Date: DD MMM YY	School Code:	Initials of Data Collector:

		4. Somewhat agree<br>5. Agree<br>6. Strongly agree	
cprac06_hours	In the last practice question, you need to type in the amount of time in the format below.<br><br>About how much time does it take you to get to get to your child's school?<br><br>Number of hours to get to your child's school:	*Write number of hours.*	
cprac06_min	Number of minutes to get to your child's school:	*Write number of minutes.*	
	The survey will now begin.		

Section 1: COMMUNICATION

1.1 Parent-Adolescent Communication Scale

Var Name	Item	Response Options	Response
ppacintro01	Using the scale below, please indicate how much you agree or disagree with each of the following statements about the general communication between you and your child in the study with you <u>in the past [time_point].</u>		
ppac01	I can discuss my beliefs with my child without feeling restrained or embarrassed.	1. Strongly disagree<br>2. Disagree<br>3. Somewhat disagree<br>4. Somewhat agree<br>5. Agree<br>6. Strongly agree	
ppac02	Sometimes I have trouble believing everything my child tells me.	1. Strongly disagree<br>2. Disagree<br>3. Somewhat disagree<br>4. Somewhat agree	

PID:	Date: DD MMM YY	School Code:	Initials of Data Collector:

		5. Agree<br>6. Strongly agree	
ppac03	My child is always a good listener.	1. Strongly disagree<br>2. Disagree<br>3. Somewhat disagree<br>4. Somewhat agree<br>5. Agree<br>6. Strongly agree	
ppac04	I am sometimes afraid to ask my child for what I want.	1. Strongly disagree<br>2. Disagree<br>3. Somewhat disagree<br>4. Somewhat agree<br>5. Agree<br>6. Strongly agree	
ppac05	My child has a tendency to say things which would be better left unsaid.	1. Strongly disagree<br>2. Disagree<br>3. Somewhat disagree<br>4. Somewhat agree<br>5. Agree<br>6. Strongly agree	
ppac06	My child can tell how I am feeling without asking.	1. Strongly disagree<br>2. Disagree<br>3. Somewhat disagree<br>4. Somewhat agree<br>5. Agree<br>6. Strongly agree	
ppac07	I am very satisfied with how my child and I talk together.	1. Strongly disagree<br>2. Disagree<br>3. Somewhat disagree<br>4. Somewhat agree<br>5. Agree<br>6. Strongly agree	
ppac08	If I were in trouble, I could tell my child.	1. Strongly disagree<br>2. Disagree<br>3. Somewhat disagree<br>4. Somewhat agree<br>5. Agree	

PID:	Date: DD MMM YY	School Code:	Initials of Data Collector:

		6. Strongly agree	
ppac09	I openly show affection to my child.	1. Strongly disagree<br>2. Disagree<br>3. Somewhat disagree<br>4. Somewhat agree<br>5. Agree<br>6. Strongly agree	
ppac10	When we are having a problem, I often give my child the silent treatment.	1. Strongly disagree<br>2. Disagree<br>3. Somewhat disagree<br>4. Somewhat agree<br>5. Agree<br>6. Strongly agree	
ppac11	I am careful about what I say to my child.	1. Strongly disagree<br>2. Disagree<br>3. Somewhat disagree<br>4. Somewhat agree<br>5. Agree<br>6. Strongly agree	
ppac12	When I am talking to my child, I have a tendency to say things that would be better left unsaid.	1. Strongly disagree<br>2. Disagree<br>3. Somewhat disagree<br>4. Somewhat agree<br>5. Agree<br>6. Strongly agree	
ppac13	When I ask questions, I get honest answers from my child.	1. Strongly disagree<br>2. Disagree<br>3. Somewhat disagree<br>4. Somewhat agree<br>5. Agree<br>6. Strongly agree	
ppac14	My child tries to understand my point of view.	1. Strongly disagree<br>2. Disagree<br>3. Somewhat disagree<br>4. Somewhat agree<br>5. Agree<br>6. Strongly agree	

PID:	Date: DD MMM YY	School Code:	Initials of Data Collector:

ppac15	There are topics I avoid discussing with my child.	1. Strongly disagree<br>2. Disagree<br>3. Somewhat disagree<br>4. Somewhat agree<br>5. Agree<br>6. Strongly agree	
ppac16	I find it easy to discuss problems with my child.	1. Strongly disagree<br>2. Disagree<br>3. Somewhat disagree<br>4. Somewhat agree<br>5. Agree<br>6. Strongly agree	
ppac17	It is very easy for me to express all my true feelings to my child.	1. Strongly disagree<br>2. Disagree<br>3. Somewhat disagree<br>4. Somewhat agree<br>5. Agree<br>6. Strongly agree	
ppac18	My child nags/bothers me.	1. Strongly disagree<br>2. Disagree<br>3. Somewhat disagree<br>4. Somewhat agree<br>5. Agree<br>6. Strongly agree	
ppac19	My child insults me when they are angry with me.	1. Strongly disagree<br>2. Disagree<br>3. Somewhat disagree<br>4. Somewhat agree<br>5. Agree<br>6. Strongly agree	
ppac20	I do not think I can tell my child how I really feel about some things.	1. Strongly disagree<br>2. Disagree<br>3. Somewhat disagree<br>4. Somewhat agree<br>5. Agree<br>6. Strongly agree	

PID:	Date: DD MMM YY	School Code:	Initials of Data Collector:

1.2 Frequency of parent-child communication about substance use

Var Name	Item	Response Options	Response
pfpcintro01	Below are a few topics you may or may not have ever discussed with your child in the study. Please choose the best response to the statement provided.<br><br>**DO NOT include the audio-recorded conversation you had with your child using the Parent-Child Conversation Prompts for the SUPPER Project.**		
pfpc01	Have you and your child ever talked about smoking cigarettes?	0. No<br>1. Yes	
pfpc02	During the past [time_point], how many times have you and your child talked about smoking cigarettes?	0. None<br>1. Once<br>2. A few times<br>3. Several<br>4. A lot	
pfpc03	Have you and your child ever talked about using e-cigarettes or vaping?	0. No<br>1. Yes	
pfpc04	During the past [time_point], how many times have you and your child talked about using e-cigarettes or vaping?	0. None<br>1. Once<br>2. A few times<br>3. Several<br>4. A lot	
pfpc05	Have you and your child ever talked about drinking alcohol?	0. No<br>1. Yes	
pfpc06	During the past [time_point], how many times have you and your child talked about drinking alcohol?	0. None<br>1. Once<br>2. A few times<br>3. Several<br>4. A lot	
pfpc07	Have you and your child ever talked about using marijuana?	0. No<br>1. Yes	

PID:	Date: DD MMM YY	School Code:	Initials of Data Collector:

pfpc08	During the past [time_point], how many times have you and your child talked about using marijuana?	0. None<br>1. Once<br>2. A few times<br>3. Several<br>4. A lot	
pfpc09	Have you and your child ever talked about using other drugs?	0. No<br>1. Yes	
pfpc10	During the past [time_point], how many times have you and your child talked about using other drugs?	0. None<br>1. Once<br>2. A few times<br>3. Several<br>4. A lot	

1.3 Targeted Parent-Child Communication About Drugs

Var Name	Item	Response Options	Response
ptpcintro01	Think about the conversations you have had with your child in the study, in the past [time_point]. With these conversations in mind, indicate how much you agree or disagree with each of the following statements.<br><br>**DO NOT include the audio-recorded conversation you had with your child using the Parent-Child Conversation Prompts for the SUPPER Project.**<br><br>You…		
ptpc01	Have not directly talked with your child about drugs and alcohol use but have given hints that they should not use them.	1. Strongly disagree<br>2. Disagree<br>3. Somewhat disagree<br>4. Somewhat agree<br>5. Agree<br>6. Strongly agree	
ptpc02	Have lectured or given your child a speech about drinking alcohol and using drugs.	1. Strongly disagree<br>2. Disagree<br>3. Somewhat disagree<br>4. Somewhat agree<br>5. Agree	

PID:	Date: DD MMM YY	School Code:	Initials of Data Collector:

		6. Strongly agree	
ptpc03	Have warned your child about the dangers of drinking alcohol and using drugs.	1. Strongly disagree<br>2. Disagree<br>3. Somewhat disagree<br>4. Somewhat agree<br>5. Agree<br>6. Strongly agree	
ptpc04	Have talked to your child about how to handle offers of alcoholic drinks and drugs.	1. Strongly disagree<br>2. Disagree<br>3. Somewhat disagree<br>4. Somewhat agree<br>5. Agree<br>6. Strongly agree	
ptpc05	Have given your child rules to obey about drinking alcohol and using drugs.	1. Strongly disagree<br>2. Disagree<br>3. Somewhat disagree<br>4. Somewhat agree<br>5. Agree<br>6. Strongly agree	
ptpc06	Have made a comment to your child about how drinking alcohol and using drugs is bad if a character on TV is drinking or drunk.	1. Strongly disagree<br>2. Disagree<br>3. Somewhat disagree<br>4. Somewhat agree<br>5. Agree<br>6. Strongly agree	
ptpc07	Have told your child stories of people who drink alcohol, have been drunk, or use drugs.	1. Strongly disagree<br>2. Disagree<br>3. Somewhat disagree<br>4. Somewhat agree<br>5. Agree<br>6. Strongly agree	
ptpc08	Have told your child you would be disappointed in her/him if they were to drink alcohol or use drugs.	1. Strongly disagree<br>2. Disagree<br>3. Somewhat disagree<br>4. Somewhat agree<br>5. Agree<br>6. Strongly agree	

PID:	Date: DD MMM YY	School Code:	Initials of Data Collector:

ptpc09	Have shown your child information on the web, TV, or in the news about the dangers of drinking alcohol and using drugs.	1. Strongly disagree<br>2. Disagree<br>3. Somewhat disagree<br>4. Somewhat agree<br>5. Agree<br>6. Strongly agree	
ptpc10	Have asked your child about their thoughts and opinions about drinking alcohol and using drugs.	1. Strongly disagree<br>2. Disagree<br>3. Somewhat disagree<br>4. Somewhat agree<br>5. Agree<br>6. Strongly agree	

PID:	Date: DD MMM YY	School Code:	Initials of Data Collector:

1.4 Parental Self-Efficacy

Var Name	Item	Response Options	Response
ppseintro01	How confident are you that you can talk to your child in the study about:		
ppse01	Cigarettes?	1. Not at all confident<br>2. Somewhat confident<br>3. Moderately confident<br>4. Very confident	
ppse02	E-cigarettes?	1. Not at all confident<br>2. Somewhat confident<br>3. Moderately confident<br>4. Very confident	
ppse03	Alcohol?	1. Not at all confident<br>2. Somewhat confident<br>3. Moderately confident<br>4. Very confident	
ppse04	Marijuana?	1. Not at all confident<br>2. Somewhat confident<br>3. Moderately confident<br>4. Very confident	
ppse05	Other drugs?	1. Not at all confident<br>2. Somewhat confident<br>3. Moderately confident<br>4. Very confident	
ppse06	How confident are you that you have given your child in the study the knowledge and strategies they need to help keep them from misusing drugs and alcohol?	1. Not at all confident<br>2. Somewhat confident<br>3. Moderately confident<br>4. Very confident	
ppse07	How confident are you in your ability to talk to the parents of your child's friends about your expectations regarding your child's use of alcohol and other substances?	1. Not at all confident<br>2. Somewhat confident<br>3. Moderately confident<br>4. Very confident	

1.5 Health/Weight-Related Conversations

Var Name	Item	Response Options	Response
phwrcintro01	The following set of questions are about conversations you have had with your child in the study about eating habits or physical activity.		

PID:	Date: DD MMM YY	School Code:	Initials of Data Collector:

	**DO NOT include the audio-recorded conversation you had with your child using the Parent-Child Conversation Prompts for the SUPPER Project.**<br><br>How often in the past [time_point] have you…		
phwrc01	…had a conversation with your child about healthy eating habits?	0. Never<br>1. A few times in the past [time_point]<br>2. A few times a month<br>3. A few times a week<br>4. Almost every day	
phwrc02	…had a conversation with your child about being physically active?	0. Never<br>1. A few times in the past [time_point]<br>2. A few times a month<br>3. A few times a week<br>4. Almost every day	
phwrc03	…had a conversation with your child about their weight or size?	0. Never<br>1. A few times in the past [time_point]<br>2. A few times a month<br>3. A few times a week<br>4. Almost every day	
phwrc04	…mentioned to your child that they weigh too much?	0. Never<br>1. A few times in the past [time_point]<br>2. A few times a month<br>3. A few times a week<br>4. Almost every day	
phwrc05	…mentioned to your child that they should eat differently in order to lose weight or keep from gaining weight?	0. Never<br>1. A few times in the past [time_point]<br>2. A few times a month<br>3. A few times a week<br>4. Almost every day	
phwrc06	…mentioned to your child that they should exercise in order to lose weight or to keep from gaining weight?	0. Never<br>1. A few times in the past [time_point]<br>2. A few times a month<br>3. A few times a week<br>4. Almost every day	

PID:	Date: DD MMM YY	School Code:	Initials of Data Collector:

1.6 Negative weight talk

Var Name	Item	Response Options	Response
pnwtintro01	How often in the past [time_point] have you done or said the following to your child in the study:		
pnwt01	Said they were fat.	0. Never<br>1. A few times in the past [time_point]<br>2. A few times a month<br>3. A few times a week<br>4. Almost every day	
pnwt02	Teased or made fun of them about the size and shape of their body.	0. Never<br>1. A few times in the past [time_point]<br>2. A few times a month<br>3. A few times a week<br>4. Almost every day	
pnwt03	Said they should go on a diet.	0. Never<br>1. A few times in the past [time_point]<br>2. A few times a month<br>3. A few times a week<br>4. Almost every day	
pnwt04	Said that they eat food that will make them fat.	0. Never<br>1. A few times in the past [time_point]<br>2. A few times a month<br>3. A few times a week<br>4. Almost every day	
pnwt05	Said that they would look better if they were thinner.	0. Never<br>1. A few times in the past [time_point]<br>2. A few times a month<br>3. A few times a week<br>4. Almost every day	
psnwt01	Said **about yourself** that **you** were fat or that **you** needed to go on a diet to lose weight?	0. Never<br>1. A few times in the past [time_point]<br>2. A few times a month<br>3. A few times a week<br>4. Almost every day	

PID:	Date: D D M M M Y Y	School Code:	Initials of Data Collector:

Section 2: EATING, EXERCISE, & WEIGHT

2.1 Dietary Intake

Var Name	Item	Response Options	Response
pdiintro01	The next 8 questions ask about food you ate or drank during the past 7 days. Think about all the meals and snacks you had from the time you got up until you went to bed. Be sure to include food you ate at home, at work, at restaurants, or anywhere else.		
pdi01	During the past 7 days, how many times did you eat fruit? (Do not count fruit juice.)	0. None<br>1. 1-3 times<br>2. 4-6 times<br>3. 1 time per day<br>4. 2 times per day<br>5. 3 times per day<br>6. 4 or more times per day	
pdi02	During the past 7 days, how many times did you drink 100% fruit juice such as orange juice, apple juice, or grape juice? (Do not count punch, Kool-Aid, sports drinks, or other fruit-flavored drinks.)	0. None<br>1. 1-3 times<br>2. 4-6 times<br>3. 1 time per day<br>4. 2 times per day<br>5. 3 times per day<br>6. 4 or more times per day	
pdi03	During the past 7 days, how many times did you eat vegetables (for example, green salad, carrots or any other vegetable—do not include French fries or potato chips)?	0. None<br>1. 1-3 times<br>2. 4-6 times<br>3. 1 time per day<br>4. 2 times per day<br>5. 3 times per day<br>6. 4 or more times per day	
pdi04	During the past 7 days, how many times did you drink a can, bottle, or glass of soda or pop, such as Coke, Pepsi, or Sprite? (Do not count diet soda or diet pop.)	0. None<br>1. 1-3 times<br>2. 4-6 times<br>3. 1 time per day<br>4. 2 times per day<br>5. 3 times per day<br>6. 4 or more times per day	

PID:	Date: D D M M M Y Y	School Code:	Initials of Data Collector:

pdi05	During the past 7 days, how many times did you drink a can, bottle, or glass of a sports drink such as Gatorade or Powerade? (Do not count low-calorie sports drinks such as Propel or G2.)	0. None<br>1. 1-3 times<br>2. 4-6 times<br>3. 1 time per day<br>4. 2 times per day<br>5. 3 times per day<br>6. 4 or more times per day	
pdi06	During the past 7 days, how many times did you drink a bottle or glass of plain water (Count tap, bottled and unflavored sparkling water)?	0. None<br>1. 1-3 times<br>2. 4-6 times<br>3. 1 time per day<br>4. 2 times per day<br>5. 3 times per day<br>6. 4 or more times per day	
pdi07	During the past 7 days, how many times did you drink a glass of milk? (Count the milk you drank in a glass or cup, from a carton, or with cereal).	0. None<br>1. 1-3 times<br>2. 4-6 times<br>3. 1 time per day<br>4. 2 times per day<br>5. 3 times per day<br>6. 4 or more times per day	
pdi08	During the <u>past 7 days</u>, on how many days did you eat <u>breakfast</u>?	0. None<br>1. 1 day<br>2. 2 days<br>3. 3 days<br>4. 4 days<br>5. 5 days<br>6. 6 days<br>7. 7 days	

2.2 Physical Activity Levels – Adult (IPAQ)

Var Name	Item	Response Options	Response
pipaqintro01	We are interested in finding out about the kinds of physical activities that people do as part of their everyday lives. The questions will ask you about the time you spent being physically active <u>in the last 7 days</u>. Please		

PID:	Date: DD MMM YY	School Code:	Initials of Data Collector:

	answer each question even if you do not consider yourself to be an active person. Please think about the activities you do at work, as part of your house and yard work, to get from place to place, and in your spare time for recreation, exercise or sport.		
pipaq01	Think about all the vigorous activities that you did in the last 7 days. Vigorous physical activities refer to activities that take hard physical effort and make you breathe much harder than normal. Think only about those physical activities that you did for at least 10 minutes at a time.<br><br>During the last 7 days on how many days did you do vigorous physical activities like heavy lifting, digging, aerobics, or fast bicycling?	0. No vigorous physical activities<br>1. 1 day<br>2. 2 days<br>3. 3 days<br>4. 4 days<br>5. 5 days<br>6. 6 days<br>7. 7 days	
pipaq02hr	How much time did you usually spend doing vigorous physical activities on one of those days?<br><br>*Note: Hours must be in whole numbers (1, 2, 3). If you did physical activity for less than an hour, please enter the time in minutes.*<br><br>*Examples:*<br>*Half an hour = 0 hours 30 minutes*<br>*One hour and a half = 1 hour 30 minutes*	Number of hours per day	
pipaq02min		Number of minutes per day	
pipaq03	Think about all the moderate activities that you did in the last 7 days. Moderate activities refer to activities that take moderate physical effort and make you breathe somewhat harder than normal. Think only about those physical activities that you did for at least 10 minutes at a time.<br><br>During the last 7 days, on how many days did you do moderate physical activities like carrying light loads, bicycling at a regular pace, or doubles tennis? Do not include walking.	0. No moderate physical activities<br>1. 1 day<br>2. 2 days<br>3. 3 days<br>4. 4 days<br>5. 5 days<br>6. 6 days<br>7. 7 days	

PID:	Date: DD MMM YY	School Code:	Initials of Data Collector:

pipaq04hr	How much time did you usually spend doing moderate physical activities on one of those days?	Number of hours per day	
pipaq04min		Number of minutes per day	
pipaq05	Think about the time you spent walking in the last 7 days. This includes at work and at home, walking to travel from place to place, and any other walking that you have done solely for recreation, sport, exercise, or leisure.<br><br>During the last 7 days, on how many days did you walk for at least 10 minutes at a time?	0. No walking<br>1. 1 day<br>2. 2 days<br>3. 3 days<br>4. 4 days<br>5. 5 days<br>6. 6 days<br>7. 7 days	
pipaq06hr	How much time did you spend walking on one of those days?	Number of hours per day	
pipaq06min		Number of minutes per day	
pipaq07hr	The last question is about the time you spent sitting on weekdays during the last 7 days. Include time spent at work, at home, while doing course work and during leisure time. This may include time spent sitting at a desk, visiting friends, reading, or sitting or lying down to watch television.<br><br>During the last 7 days, how much time did you spend sitting on a week day?	Number of hours per weekday	
pipaq07min		Number of minutes per weekday	

PID:	Date: DD MMM YY	School Code:	Initials of Data Collector:

2.3 Parent Self-reported weight

Var Name	Item	Response Options	Response
pwt01	About how much do you weigh without clothes or shoes?	____ pounds	
pwt02ft	About how tall are you without shoes?	____ feet	
pwt02in		____ inches	

2.4 Parent own weight description

Var Name	Item	Response Options	Response
pwd01	How do you describe your weight?	1. Very underweight<br>2. Slightly underweight<br>3. About the right weight<br>4. Slightly overweight<br>5. Very overweight	
pwd02	Which of the following are you trying to do about your weight?	1. Lose weight<br>2. Gain weight<br>3. Stay the same weight<br>4. I am not trying to do anything about my weight	

2.5 Modified Weight Bias Internalization Scale

Var Name	Item	Response Options	Response
pwbismintro01	The following statements are about how you feel about your current weight.<br><br>Read each statement below and answer with how much you agree or disagree with each statement.		
pwbism01	I am less attractive than most other people because of my weight.	1. Strongly disagree<br>2. Disagree<br>3. Somewhat disagree<br>4. Somewhat agree<br>5. Agree<br>6. Strongly agree	
pwbism02	I feel anxious about my weight because of what people might think of me.	1. Strongly disagree<br>2. Disagree<br>3. Somewhat disagree<br>4. Somewhat agree	

PID:	Date: DD MMM YY	School Code:	Initials of Data Collector:

		5. Agree<br>6. Strongly agree	
pwbism03	I wish I could drastically change my weight.	1. Strongly disagree<br>2. Disagree<br>3. Somewhat disagree<br>4. Somewhat agree<br>5. Agree<br>6. Strongly agree	
pwbism04	Whenever I think a lot about my weight, I feel depressed.	1. Strongly disagree<br>2. Disagree<br>3. Somewhat disagree<br>4. Somewhat agree<br>5. Agree<br>6. Strongly agree	
pwbism05	I hate myself for my weight.	1. Strongly disagree<br>2. Disagree<br>3. Somewhat disagree<br>4. Somewhat agree<br>5. Agree<br>6. Strongly agree	
pwbism06	My weight is a major way that I judge my value as a person.	1. Strongly disagree<br>2. Disagree<br>3. Somewhat disagree<br>4. Somewhat agree<br>5. Agree<br>6. Strongly agree	
pwbism07	I don't feel that I deserve to have a really fulfilling social life because of my weight.	1. Strongly disagree<br>2. Disagree<br>3. Somewhat disagree<br>4. Somewhat agree<br>5. Agree<br>6. Strongly agree	
pwbism08	I am OK being the weight that I am.	1. Strongly disagree<br>2. Disagree<br>3. Somewhat disagree<br>4. Somewhat agree	

PID:	Date: DD MMM YY	School Code:	Initials of Data Collector:

		5. Agree<br>6. Strongly agree	
pwbism09	Because of my weight, I don't feel like my true self.	1. Strongly disagree<br>2. Disagree<br>3. Somewhat disagree<br>4. Somewhat agree<br>5. Agree<br>6. Strongly agree	
pwbism10	Because of my weight, I don't understand how anyone attractive would want to date me.	1. Strongly disagree<br>2. Disagree<br>3. Somewhat disagree<br>4. Somewhat agree<br>5. Agree<br>6. Strongly agree	

2.6 Weight stigma experience by peers

Var Name	Item	Response Options	Response
pwsp01	Have you ever been teased or made fun of by peers because of your weight?	0. No<br>1. Yes	
pwsp02	Have you ever been treated unkindly by peers because of your weight?	0. No<br>1. Yes	

2.7 Weight stigma experience by family members

Var Name	Item	Response Options	Response
pwsf01	Have you ever been teased or made fun of by family members because of your weight?	0. No<br>1. Yes	
pwsf02	Have you ever been treated unkindly by family members because of your weight?	0. No<br>1. Yes	

2.8 Parent-Reported Child Ht/Wt

Var Name	Item	Response Options	Response
pprcw01	About how much does your child in the study weigh without clothes or shoes?	____ pounds	
pprcw02ft	About how tall is your child in the study without shoes?	____ feet	
pprcw02in		____ inches	

PID:	Date: DD MMM YY	School Code:	Initials of Data Collector:

2.9 Parent Perception of Weight Teasing

Var Name	Item	Response Options	Response
ppwt01	Has your child in the study ever been teased, treated unfairly, or been discriminated against because of their weight?	0. No<br>1. Yes	
ppwt02	During the past [time_point], has your child in the study been teased, treated unfairly, or been discriminated against because of their weight?	0. No<br>1. Yes	

Section 3: USING SUBSTANCES

3.1.1 Cigarette Definition

Var Name	Item	Response Options	Response
pcdintro01	The following set of questions will be about cigarettes. By cigarettes we mean tobacco cigarettes, this does NOT INCLUDE e-cigarettes (vaping/JUULing), hookah, or other tobacco products. 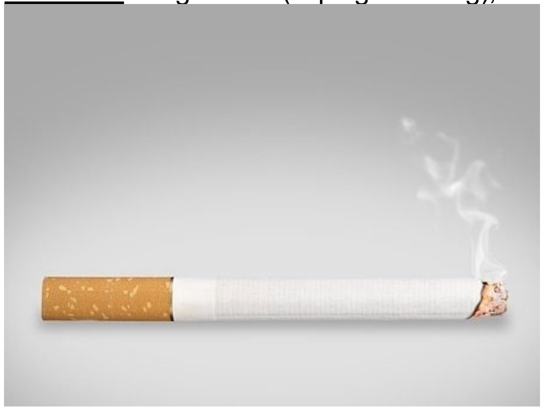		

3.1.2 Cigarette Use

Var Name	Item	Response Options	Response
pcu01	Have you ever smoked a cigarette?	0. No<br>1. Yes	

PID:	Date: DD MMM YY	School Code:	Initials of Data Collector:

pcu02	How old were you the **first time** you smoked part or all of a cigarette?	*Age in years*	
pcu03	How many times in the past [time_point] did you smoke a cigarette?	0. Never<br>1. Once a month or less<br>2. About once a week<br>3. Several times a week<br>4. About once a day<br>5. Several times a day	

3.1.3 Other Tobacco Products use

Var Name	Item	Response Options	Response
potu01	Have you ever used other tobacco products, such as chewing tobacco, snuff, dip, snus, or dissolvable tobacco products, such as Copenhagen, Grizzly, Skoal, or Camel Snus (do not count any electronic vapor products)?	0. No<br>1. Yes	
potu02	How old were you the **first time** you used other tobacco products, such as chewing tobacco, snuff, dip, snus, or dissolvable tobacco products (do not count any electronic vapor products)?	*Age in years*	
potu03	How many times in the past [time_point] did you use other tobacco products (do not count any electronic vapor products)?	0. Never<br>1. Once a month or less<br>2. About once a week<br>3. Several times a week<br>4. About once a day<br>5. Several times a day	

3.2.1 E-cigarette definition

Var Name	Item	Response Options	Response
pecdintro01	The following set of questions will be about **e-cigarettes**. By e-cigarettes we mean any electronic vapor product such as JUUL, Vuse, MarkTen, and blu. Electronic vapor products include e-cigarettes, vapes, vape pens, e-cigars, ehookahs, hookah pens, and mods.		

PID:	Date: DD MMM YY	School Code:	Initials of Data Collector:

	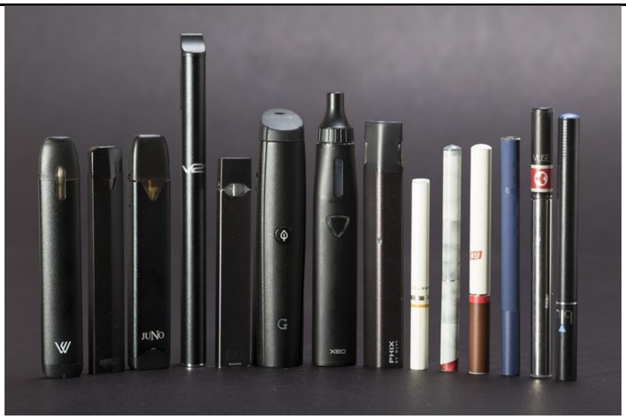		

3.2.2 E-cigarette use

Var Name	Item	Response Options	Response
pecu01	Have you ever used an e-cigarette/vape/JUUL?	0. No<br>1. Yes	
pecu02	How old were you the **first time** you used an e-cigarette/vape/JUUL?	*Age in years*	
pecu03	How many times in the past [time_point] did you use an e-cigarette/vape/JUUL?	0. Never<br>1. Once a month or less<br>2. About once a week<br>3. Several times a week<br>4. About once a day<br>5. Several times a day	

3.3.1 Alcohol Definition

Variable Name	Item	Response Options	Response
padintro01	The following set of questions will be about alcohol. By alcohol we mean any alcoholic beverage such as, beer, wine, wine coolers, and liquor (whiskey, rum, vodka, or gin). We do not mean alcohol that people drink for religious reasons.		

PID:	Date: DD MMM YY	School Code:	Initials of Data Collector:

3.3.2 Alcohol Use

Var Name	Item	Response Options	Response
pau01	Have you **ever**, even once, had a drink of any type of alcoholic beverage? Please do not include times when you only had a sip or two from a drink.	0. No<br>1. Yes	
pau02	Think about the **first time** you had a drink of an alcoholic beverage. How old were you the **first time** you had a drink of an alcoholic beverage? Please do not include any time when you only had a sip or two from a drink.	*Age in years*	
pau03	How many times in the past [time_point] did you have a drink of alcohol? Please do not include times when you only had a sip or two from a drink.	0. Never<br>1. Once a month or less<br>2. About once a week<br>3. Several times a week<br>4. About once a day<br>5. Several times a day	

3.4.1 Marijuana Definition

Var Name	Item	Response Options	Response
pmdintro01	The following set of questions will be about marijuana. By marijuana we also mean pot, weed, or cannabis that you may have smoked, vaped, or eaten.		

PID:	Date: DD MMM YY	School Code:	Initials of Data Collector:

	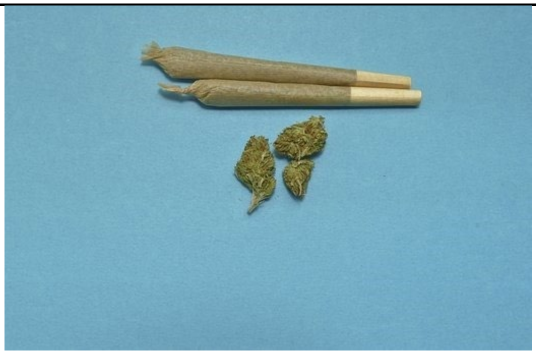		

3.4.2 Marijuana Use

Var Name	Item	Response Options	Response
pmu01	Have you ever, even once, used marijuana?	0. No<br>1. Yes	
pmu02	How old were you the **first time** you used marijuana or hashish?	*Age in years*	
pmu03	How many times in the past [time_point] did you use marijuana?	0. Never<br>1. Once a month or less<br>2. About once a week<br>3. Several times a week<br>4. About once a day<br>5. Several times a day	

3.5.1 Other Drugs Definition

Var Name	Item	Response Options	Response
poddintro01	The following set of questions will be about **other drugs**. By other drugs we mean prescription drugs used for non-medical reasons, inhalants, and other substances used to get high, which can include cocaine (including powder, crack, or freebase), steroids taken without a doctor's prescription), heroin, or any other substance that can be used to get high.		

PID:	Date: DD MMM YY	School Code:	Initials of Data Collector:

	For this section, "other drugs" **does not include** alcohol, tobacco, e-cigarettes, or marijuana,		

3.5.2 Other drug use

Var Name	Item	Response Options	Response
podu01	Have you ever used other drugs?	0. No<br>1. Yes	
podu02	How old were you the **first time** you used other drugs?	*Age in years*	
podu03	How many times in the past [time_point] did you use other drugs?	0. Never<br>1. Once a month or less<br>2. About once a week<br>3. Several times a week<br>4. About once a day<br>5. Several times a day	

3.6 Parental AUDIT

Var Name	Item	Response Options	Response
pauditintro01	These are some questions about your use of alcoholic beverages during the past [time_point]. Please choose your best answer to each question.		
paudit01	How often do you have a drink containing alcohol?	0. Never<br>1. Monthly or less<br>2. 2 to 4 times a month<br>3. 2 to 3 times a week<br>4. 4 or more times a week	
paudit02	How many drinks containing alcohol do you have on a typical day when you are drinking?	0. 1 or 2<br>1. 3 or 4<br>2. 5 or 6<br>3. 7, 8, or 9<br>4. 10 or more	
paudit03	How often do you have six or more drinks on one occasion?	0. Never	

PID:	Date: DD MMM YY	School Code:	Initials of Data Collector:

		1. Less than monthly<br>2. Monthly<br>3. Weekly<br>4. Daily or almost daily	
paudit04	How often during the last [time_point] have you found that you were not able to stop drinking once you had started?	0. Never<br>1. Less than monthly<br>2. Monthly<br>3. Weekly<br>4. Daily or almost daily	
paudit05	How often during the last [time_point] have you failed to do what was normally expected of you because of drinking?	0. Never<br>1. Less than monthly<br>2. Monthly<br>3. Weekly<br>4. Daily or almost daily	
paudit06	How often during the last [time_point] have you needed a first drink in the morning to get yourself going after a heavy drinking session?	0. Never<br>1. Less than monthly<br>2. Monthly<br>3. Weekly<br>4. Daily or almost daily	
paudit07	How often during the last [time_point] have you had a feeling of guilt or remorse after drinking?	0. Never<br>1. Less than monthly<br>2. Monthly<br>3. Weekly<br>4. Daily or almost daily	
paudit08	How often during the last [time_point] have you been unable to remember what happened the night before because of your drinking?	0. Never<br>1. Less than monthly<br>2. Monthly<br>3. Weekly<br>4. Daily or almost daily	
paudit09	Have you or someone else been injured because of your drinking?	0. No<br>1. Yes, but not in the last year<br>2. Yes, during the last year	
paudit10	Has a relative, friend, doctor, or other health care worker been concerned about your drinking or suggested you cut down?	0. No<br>1. Yes, but not in the last year<br>2. Yes, during the last year	

PID:	Date: DD MMM YY	School Code:	Initials of Data Collector:

3.7 Parental DAST

Var Name	Item	Response Options	Response
pdastintro01	The following questions ask for information about your potential drug use during the PAST [time_point].<br><br>"Drug abuse" means (1) use of prescribed or over-the-counter drugs in excess of the directions, and (2) any use of drugs for non-medical reasons. The various classes of drugs may include but are not limited to: cannabis (e.g., marijuana, hash), solvents (e.g., gas, paints etc), tranquilizers (e.g., Valium), barbiturates, cocaine, and stimulants (e.g., speed), hallucinogens (e.g., LSD) or narcotics (e.g., Heroin). Remember that the questions do not include alcohol or tobacco.<br><br>Please answer every question. If you have difficulty with a question, then choose the response that is mostly right.<br><br>These questions refer to the past [time_point] ONLY.<br><br>Please select one answer for each line.		
pdast01	Have you ever used drugs other than those required for medical reasons?	0. No<br>1. Yes	
pdast02	Do you abuse more than one drug at a time?	0. No<br>1. Yes	
pdast03	Are you always able to stop using drugs when you want to?	0. No<br>1. Yes	
pdast04	Have you had "blackouts" or "flashbacks" as a result of drug use?	0. No<br>1. Yes	
pdast05	Do you ever feel bad or guilty about your drug use?	0. No<br>1. Yes	
pdast06	Does your partner (or parent) ever complain about your involvement with drugs?	0. No<br>1. Yes	
pdast07	Have you neglected your family because of your use of drugs?	0. No<br>1. Yes	
pdast08	Have you engaged in illegal activities in order to obtain drugs?	0. No<br>1. Yes	

PID:	Date: DD MMM YY	School Code:	Initials of Data Collector:

pdast09	Have you ever experienced withdrawal symptoms (felt sick) when you stopped taking drugs?	0. No<br>1. Yes	
pdast10	Have you had medical problems as a result of your drug use (e.g., memory loss, hepatitis, convulsions, bleeding, etc)?	0. No<br>1. Yes	

3.8 Parental problem with substance use

Var Name	Item	Response Options	Response
ppwsu01	Do you think you ever had a problem with your own drug or alcohol use?	0. No<br>1. Yes	
ppwsu02	Have you ever received treatment or counseling for your use of alcohol or any drug, not counting cigarettes?	0. No<br>1. Yes	
ppwsu03	At this time do you consider yourself to be in recovery or recovered from your own problem with drugs or alcohol use?	0. No<br>1. Yes	
ppwsu04	During the past [time_point], do you think you have had a problem with your own drug or alcohol use?	0. No<br>1. Yes	
ppwsu05	During the past [time_point], have you received treatment or counseling for your use of alcohol or any drug, not counting cigarettes?	0. No<br>1. Yes	

PID:	Date: DD MMM YY	School Code:	Initials of Data Collector:

3.9 Attitude and Perception Survey on Youth Alcohol, Tobacco and Drug Use

Var Name	Item	Response Options	Response
ppatuintro01	The next set of questions are about YOUR own thoughts, feelings and beliefs. We are interested in what you think, not what others might think.<br><br>How wrong do you feel it would be for your child in the study to…		
ppatu01	Drink beer, wine or hard liquor (for example, vodka, whisky or gin) regularly (at least once or twice a month)?	1. Very wrong<br>2. Wrong<br>3. A little bit wrong<br>4. Not wrong at all	
ppatu02	Smoke cigarettes?	1. Very wrong<br>2. Wrong<br>3. A little bit wrong<br>4. Not wrong at all	
ppatu03	Use e-cigarettes or vapes/JUULs?	1. Very wrong<br>2. Wrong<br>3. A little bit wrong<br>4. Not wrong at all	
ppatu04	Use marijuana?	1. Very wrong<br>2. Wrong<br>3. A little bit wrong<br>4. Not wrong at all	
ppatu05	Use other drugs?	1. Very wrong<br>2. Wrong<br>3. A little bit wrong<br>4. Not wrong at all	

PID:	Date: D D M M M Y Y	School Code:	Initials of Data Collector:

3.10 Parental Report of (other) Child and Adult Substance Use

Var Name	Item	Response Options	Response
pocas01	The following questions are about the drug and alcohol use of others in your home. Remember all responses are kept private.<br><br>Do you think anyone in your household has ever had a problem with their drug or alcohol use?	0. No<br>1. Yes	
pocas02	Has anyone in your household ever received treatment or counseling for your use of alcohol or any drug, not counting cigarettes?	0. No<br>1. Yes	
pocas03	During the past [time_point], do you think anyone in your household has had problem with their drug or alcohol use?	0. No<br>1. Yes	
pocas04	During the past [time_point], has anyone in your household received treatment or counseling for your use of alcohol or any drug, not counting cigarettes?	0. No<br>1. Yes	

PID:	Date: DD MMM YY	School Code:	Initials of Data Collector:

Section 4: HOW YOU FEEL ABOUT YOURSELF

4.1 Self-esteem

Variable Name	Question	Response Options	Response
prosenberg01	The next section is asking about how you feel about yourself. Indicate how much you agree or disagree with each of the following statements.<br><br>I feel that I am a person of worth, at least on an equal plane with others.	1. Strongly disagree<br>2. Disagree<br>3. Somewhat disagree<br>4. Somewhat agree<br>5. Agree<br>6. Strongly agree	
prosenberg02	I feel that I have a number of good qualities.	1. Strongly disagree<br>2. Disagree<br>3. Somewhat disagree<br>4. Somewhat agree<br>5. Agree<br>6. Strongly agree	
prosenberg03	All in all, I am inclined to feel that I am a failure.	1. Strongly disagree<br>2. Disagree<br>3. Somewhat disagree<br>4. Somewhat agree<br>5. Agree<br>6. Strongly agree	
prosenberg04	I am able to do things as well as most other people.	1. Strongly disagree<br>2. Disagree<br>3. Somewhat disagree<br>4. Somewhat agree<br>5. Agree<br>6. Strongly agree	
prosenberg05	I feel I do not have much to be proud of.	1. Strongly disagree<br>2. Disagree<br>3. Somewhat disagree<br>4. Somewhat agree<br>5. Agree<br>6. Strongly agree	
prosenberg06	I take a positive attitude toward myself.	1. Strongly disagree<br>2. Disagree<br>3. Somewhat disagree	

PID:	Date: DD MMM YY	School Code:	Initials of Data Collector:

		4. Somewhat agree<br>5. Agree<br>6. Strongly agree	
prosenberg07	On the whole, I am satisfied with myself.	1. Strongly disagree<br>2. Disagree<br>3. Somewhat disagree<br>4. Somewhat agree<br>5. Agree<br>6. Strongly agree	
prosenberg08	I wish I could have more respect for myself.	1. Strongly disagree<br>2. Disagree<br>3. Somewhat disagree<br>4. Somewhat agree<br>5. Agree<br>6. Strongly agree	
prosenberg09	I certainly feel useless at times.	1. Strongly disagree<br>2. Disagree<br>3. Somewhat disagree<br>4. Somewhat agree<br>5. Agree<br>6. Strongly agree	
prosenberg10	At times I think I am no good at all.	1. Strongly disagree<br>2. Disagree<br>3. Somewhat disagree<br>4. Somewhat agree<br>5. Agree<br>6. Strongly agree	

4.2 Mental Health

Variable Name	Question	Response Options	Required
ppscintro01	Please say how often each of the following has been true <u>for your child in the study</u> in the past <u>[time_point]</u>:		
ppsc01	Fidgety, unable to sit still.	0. Never<br>1. Sometimes<br>2. Often	
ppsc02	Feels sad, unhappy.	0. Never	

PID:	Date: D D M M M Y Y	School Code:	Initials of Data Collector:

		1. Sometimes<br>2. Often	
ppsc03	Daydreams too much.	0. Never<br>1. Sometimes<br>2. Often	
ppsc04	Refuses to share.	0. Never<br>1. Sometimes<br>2. Often	
ppsc05	Does not understand other people's feelings.	0. Never<br>1. Sometimes<br>2. Often	
ppsc06	Feels hopeless.	0. Never<br>1. Sometimes<br>2. Often	
ppsc07	Has trouble concentrating.	0. Never<br>1. Sometimes<br>2. Often	
ppsc08	Fights with other children.	0. Never<br>1. Sometimes<br>2. Often	
ppsc09	Are down on themselves.	0. Never<br>1. Sometimes<br>2. Often	
ppsc10	Blame others for their troubles.	0. Never<br>1. Sometimes<br>2. Often	
ppsc11	Seems to be having less fun.	0. Never<br>1. Sometimes<br>2. Often	
ppsc12	Does not listen to rules.	0. Never<br>1. Sometimes<br>2. Often	
ppsc13	Acts as if driven by a motor.	0. Never<br>1. Sometimes<br>2. Often	
ppsc14	Teases others.	0. Never	

PID:	Date: DD MMM YY	School Code:	Initials of Data Collector:

		1. Sometimes<br>2. Often	
ppsc15	Worries a lot.	0. Never<br>1. Sometimes<br>2. Often	
ppsc16	Takes things that do not belong to them.	0. Never<br>1. Sometimes<br>2. Often	
ppsc17	Distracted easily.	0. Never<br>1. Sometimes<br>2. Often	

Section 5: YOUR HOME & NEIGHBORHOOD

5.1 Household structure (follow up)

Var Name	Item	Response Options	Response
phsfintro01	The next section asks about your home and neighborhood.<br><br>We define household members as people who sleep and eat in the same residence as you most of the time.		
phsf01	Does Name 1 (relationship to child) still live with you?	0. No<br>1. Yes	
phsf01_spec	Please explain, in a few words, why this change in household structure has happened:	TEXT ENTRY	
phsf02	Does anyone else (not previously mentioned) live in your house?	0. No<br>1. Yes	
phsf03	Please list the first name(s) of anyone else (not previously mentioned) living in your house	List all names	
phsf04	What is the relationship of [Name written on line #xxx] to the participant child?	1. Biological mother<br>2. Biological Father<br>3. Step-mother<br>4. Step-father<br>5. Adoptive mother	

PID:	Date: DD MMM YY	School Code:	Initials of Data Collector:

		6. Adoptive father<br>7. Foster mother<br>8. Foster father<br>9. Grandmother<br>10. Grandfather<br>11. Biological sister<br>12. Biological brother<br>13. Step-sister<br>14. Step-brother<br>15. Foster sister<br>16. Foster brother<br>17. Adopted sister<br>18. Adopted brother<br>19. Other	
phsf04_spec	Please specify their relationship to the child in the study.	TEXT ENTRY	
phsf05	Please explain, in a few words, why this change in household structure has happened:	TEXT ENTRY	

5.2 Lives with child 50% of the time

Var Name	Item	Response Options	Response
plwc01	Does your child in the study live with you at least 50% (half) of the time?	0. No<br>1. Yes	
plwc02	Please explain, in a few words, what has changed:	___________	

5.3 School enrollment

Var Name	Item	Response Options	Response
pse01	Is your child in the study currently enrolled in school this academic year?	1. Yes (public, private, home study)<br>2. No, summer/vacation<br>3. No, quit<br>4. No, suspended/expelled	

PID:	Date: DD MMM YY	School Code:	Initials of Data Collector:

5.4 Family Meals Descriptive

Var Name	Item	Response Options	Response
pfddintro01	The following questions will ask you about family meals. In this study, we define family meals as the time when you join your child in the study for a meal and one of you is eating, regardless of the type of food that is served. It is still considered a family meal if you are present while your child eats but you do not eat the meal yourself (and vice versa).		
pfdd01	The following question is about family breakfasts. We define breakfast as your first meal of the day.<br><br>During a typical week in the past [time_point], how many breakfasts do you usually eat with your child?	0. 0 breakfasts<br>1. 1 breakfast<br>2. 2 breakfasts<br>3. 3 breakfasts<br>4. 4 breakfasts<br>5. 5 breakfasts<br>6. 6 breakfasts<br>7. 7 breakfasts	
pfdd02	The following question is about family lunch. We define lunch as the meal you eat in the middle of the day, between breakfast and dinner.<br><br>During a typical week in the past [time_point], how many lunches do you usually eat with your child?	0. 0 lunches<br>1. 1 lunch<br>2. 2 lunches<br>3. 3 lunches<br>4. 4 lunches<br>5. 5 lunches<br>6. 6 lunches<br>7. 7 lunches	
pfdd03	The following questions will ask you about family dinners. We define dinner as the meal you eat at the end of the day.<br><br>During a typical week in the past [time_point], how many dinners do you eat with your child?	0. 0 dinners<br>1. 1 dinner<br>2. 2 dinners<br>3. 3 dinners<br>4. 4 dinners<br>5. 5 dinners<br>6. 6 dinners<br>7. 7 dinners	
pfdd04	During a typical week in the past [time_point], about how many minutes do family dinners usually last (do not include the time it takes to make the meal or clean it up)?	0. We never have family dinner<br>1. 0 - 15 minutes<br>2. 16 - 30 minutes	

PID:	Date: DD MMM YY	School Code:	Initials of Data Collector:

		3. 31 - 45 minutes<br>4. 46 - 60 minutes<br>5. More than 60 minutes	
pfdd05	During a typical week in the past [time_point], how many people are usually at family dinners?	0. We never have family dinner<br>1. 2 people<br>2. 3 people<br>3. 4 people<br>4. 5 people<br>5. 6 people<br>6. 7 people<br>7. 8 people<br>8. 9 people<br>9. 10 or more people	

5.5 Family Dinner Index

Var Name	Item	Response Options	Response
pfdiintro01	When we say family dinner, we mean the last meal of the day, as long as you are with your child in the study, even if you are not eating. If you do not eat dinner with your child, think about the meal you eat most frequently together.		
pfdi01	Think about family dinners during a typical week in the past [time_point]:<br><br>How often is your child supposed to be at dinners with you?	0. Never<br>1. Rarely<br>2. Sometimes<br>3. Often<br>4. Always	
pfdi02	How much do you think your child in the study likes having dinner with you?	0. Not at all<br>1. A little<br>2. Somewhat<br>3. A lot<br>4. Very much	
pfdi03	Think about family dinners in the past [time_point] during a typical week:	0. Not at all	

PID:	Date: DD MMM YY	School Code:	Initials of Data Collector:

	How much do you enjoy family dinners in general (note: this does not include the food that is served)?	1. A little<br>2. Somewhat<br>3. A lot<br>4. Very much	
pfdi04	Think about family dinners during a typical week in the past [time_point]:<br><br>In general, how much do people talk to each other during family dinners?	0. Not at all<br>1. A little<br>2. Somewhat<br>3. A lot<br>4. Very much	
pfdi05	Think about family dinners during a typical week in the past [time_point]:<br><br>How often do people talk, send messages, or watch something during family dinners using personal devices (for example, phones)?	0. Never<br>1. Rarely<br>2. Sometimes<br>3. Often<br>4. Always	
pfdi06	Think about family dinners in the past [time_point] during a typical week:<br><br>How much does your child in the study participate in the conversation during family dinners?	0. Not at all<br>1. A little<br>2. Somewhat<br>3. A lot<br>4. Very much	
pfdi07	Think about family dinners in the past [time_point] during a typical week:<br><br>Of the foods served at family dinners, your child is able to choose how much they eat.	1. Strongly disagree<br>2. Disagree<br>3. Not sure<br>4. Agree<br>5. Strong agree	
pfdi08	Think about family dinners in the past [time_point] during a typical week:<br><br>Of the foods served at family dinners, your child is able to choose the ones that they eat.	1. Strongly disagree<br>2. Disagree<br>3. Not sure<br>4. Agree<br>5. Strong agree	
pfdi09	Think about family dinners in the past [time_point] during a typical week:<br><br>How often do people actively watch shows, movies, or sports games during family dinners (when it is not just on in the background)?	0. Never<br>1. Rarely<br>2. Sometimes<br>3. Often	

PID:	Date: DD MMM YY	School Code:	Initials of Data Collector:

		4. Always	
pfdi10	Parents have different reasons for eating family dinners with their child. Think about family dinners in the past [time_point] during a typical week:<br><br>How important are family dinners for making parent-child relationships stronger?	0. Not at all<br>1. A little<br>2. Somewhat<br>3. A lot<br>4. Very much	
pfdi11	Parents have different reasons for eating family dinners with their child. Think about family dinners in the past [time_point] during a typical week:<br><br>How important are family dinners for teaching about values and life?	0. Not at all<br>1. A little<br>2. Somewhat<br>3. A lot<br>4. Very much	

5.6 Parenting Styles and Dimensions Questionnaire (PSDQ)

Var Name	Item	Response Options	Response
ppsdqintro	The following questions ask about the parenting practices and strategies you use with your child. There are no right or wrong answers to these questions and your responses will remain private.<br><br>For each question, answer how often you use the described parenting strategy with your child in the study.		
ppsdq01	How often are you responsive to your child's feelings or needs?	0. Never<br>1. Once in a while<br>2. About half of the time<br>3. Very often<br>4. Always	
ppsdq02	How often do you use physical punishment as a way of disciplining your child?	0. Never<br>1. Once in a while<br>2. About half of the time<br>3. Very often<br>4. Always	
ppsdq03	How often do you take your child's desires into account before asking your child to do something?	0. Never<br>1. Once in a while	

PID:	Date: DD MMM YY	School Code:	Initials of Data Collector:

		2. About half of the time<br>3. Very often<br>4. Always	
ppsdq04	When your child asks why they have to conform, how often do you state: "because I said so," or "I am your parent and I want you to"?	0. Never<br>1. Once in a while<br>2. About half of the time<br>3. Very often<br>4. Always	
ppsdq05	How often do you explain to your child how you feel about their good and bad behavior?	0. Never<br>1. Once in a while<br>2. About half of the time<br>3. Very often<br>4. Always	
ppsdq06	How often do you spank your child when they are disobedient?	0. Never<br>1. Once in a while<br>2. About half of the time<br>3. Very often<br>4. Always	
ppsdq07	How often do you encourage your child to talk about their troubles?	0. Never<br>1. Once in a while<br>2. About half of the time<br>3. Very often<br>4. Always	
ppsdq08	How often do you find it difficult to discipline your child?	0. Never<br>1. Once in a while<br>2. About half of the time<br>3. Very often<br>4. Always	
ppsdq09	How often do you encourage your child to freely express themselves even when they disagree with you?	0. Never<br>1. Once in a while<br>2. About half of the time<br>3. Very often<br>4. Always	
ppsdq10	How often do you punish your child by taking privileges away with little if any explanations?	0. Never<br>1. Once in a while<br>2. About half of the time<br>3. Very often	

PID:	Date: DD MMM YY	School Code:	Initials of Data Collector:

		4. Always	
ppsdq11	How often do you emphasize the reasons for rules?	0. Never<br>1. Once in a while<br>2. About half of the time<br>3. Very often<br>4. Always	
ppsdq12	How often do you give comfort and understanding when your child is upset?	0. Never<br>1. Once in a while<br>2. About half of the time<br>3. Very often<br>4. Always	
ppsdq13	How often do you yell or shout when your child misbehaves?	0. Never<br>1. Once in a while<br>2. About half of the time<br>3. Very often<br>4. Always	
ppsdq14	How often do you give praise when your child is good?	0. Never<br>1. Once in a while<br>2. About half of the time<br>3. Very often<br>4. Always	
ppsdq15	How often do you give into your child when they cause a commotion about something?	0. Never<br>1. Once in a while<br>2. About half of the time<br>3. Very often<br>4. Always	
ppsdq16	How often do you explode in anger towards child?	0. Never<br>1. Once in a while<br>2. About half of the time<br>3. Very often<br>4. Always	
ppsdq17	How often do you threaten your child with punishment more often than actually giving it?	0. Never<br>1. Once in a while<br>2. About half of the time<br>3. Very often<br>4. Always	

PID:	Date: D D M M M Y Y	School Code:	Initials of Data Collector:

ppsdq18	How often do you take into account your child's preferences in making plans for the family?	0. Never<br>1. Once in a while<br>2. About half of the time<br>3. Very often<br>4. Always	
ppsdq19	How often do you grab your child when being disobedient?	0. Never<br>1. Once in a while<br>2. About half of the time<br>3. Very often<br>4. Always	
ppsdq20	How often do you state punishments to your child and do not actually do them?	0. Never<br>1. Once in a while<br>2. About half of the time<br>3. Very often<br>4. Always	
ppsdq21	How often do you show respect for your child's opinions by encouraging your child to express them?	0. Never<br>1. Once in a while<br>2. About half of the time<br>3. Very often<br>4. Always	
ppsdq22	How often do you allow your child to give input into family rules?	0. Never<br>1. Once in a while<br>2. About half of the time<br>3. Very often<br>4. Always	
ppsdq23	How often do you scold and criticize to make your child improve?	0. Never<br>1. Once in a while<br>2. About half of the time<br>3. Very often<br>4. Always	
ppsdq24	How often do you spoil your child?	0. Never<br>1. Once in a while<br>2. About half of the time<br>3. Very often<br>4. Always	
ppsdq25	How often do you give your child reasons why rules should be obeyed?	0. Never<br>1. Once in a while	

PID:	Date: DD MMM YY	School Code:	Initials of Data Collector:

		2. About half of the time<br>3. Very often<br>4. Always	
ppsdq26	How often do you use threats as punishment with little or no justification?	0. Never<br>1. Once in a while<br>2. About half of the time<br>3. Very often<br>4. Always	
ppsdq27	How often do you have warm and intimate times together with your child?	0. Never<br>1. Once in a while<br>2. About half of the time<br>3. Very often<br>4. Always	
ppsdq28	How often do you punish by putting your child off somewhere alone with little if any explanations?	0. Never<br>1. Once in a while<br>2. About half of the time<br>3. Very often<br>4. Always	
ppsdq29	How often do you help your child to understand the impact of behavior by encouraging child to talk about the consequences of their own actions?	0. Never<br>1. Once in a while<br>2. About half of the time<br>3. Very often<br>4. Always	
ppsdq30	How often do you scold and criticize when your child's behavior doesn't meet your expectations?	0. Never<br>1. Once in a while<br>2. About half of the time<br>3. Very often<br>4. Always	
ppsdq31	How often do you explain the consequences of your child's behavior?	0. Never<br>1. Once in a while<br>2. About half of the time<br>3. Very often<br>4. Always	
ppsdq32	How often do you slap your child when your child misbehaves?	0. Never<br>1. Once in a while<br>2. About half of the time<br>3. Very often	

PID:	Date: D D M M M Y Y	School Code:	Initials of Data Collector:

		4. Always	

PID:	Date: DD MMM YY	School Code:	Initials of Data Collector:

5.7 Child's Adverse Experiences

Var Name	Item	Response Options	Response
paceintro01	The last set of questions asks for information about you and your family. All information is kept private. However, if you are uncomfortable answering any of these questions, you can skip them.<br><br>The next questions are about events that may have happened during your child's life (the one in the study). These things can happen in any family, but some people may feel uncomfortable with these questions.		
pace01	SINCE THIS CHILD WAS BORN, how often has it been very hard to get by on your family's income – hard to cover the basics like food or housing?	0. Never<br>1. Rarely<br>2. Somewhat often<br>3. Very often	
paceintro02	To the best of your knowledge, has your child EVER experienced:		
pace02	Parent or guardian divorced or separated.	0. No<br>1. Yes	
pace03	Parent or guardian died.	0. No<br>1. Yes	
pace04	Parent or guardian served time in jail.	0. No<br>1. Yes	
pace05	Saw or heard parents or adults slap, hit, kick, punch one another in the home.	0. No<br>1. Yes	
pace06	Was a victim of violence or witnessed violence in neighborhood.	0. No<br>1. Yes	
pace07	Lived with anyone who was mentally ill, suicidal, or severely depressed.	0. No<br>1. Yes	
pace08	Lived with anyone who had a problem with alcohol or drugs.	0. No<br>1. Yes	
pace09	Treated or judged unfairly because of their race or ethnic group.	0. No<br>1. Yes	

5.8 Parental Report of Child Resiliency

Var Name	Item	Response Options	Response
prcrintro01	Please indicate how much you agree or disagree with each of the following statements about your child in the study:		

PID:	Date: DD MMM YY	School Code:	Initials of Data Collector:

prcr01	My child is good in many school subjects.	1. Strongly disagree<br>2. Disagree<br>3. Somewhat disagree<br>4. Somewhat agree<br>5. Agree<br>6. Strongly agree	
prcr02	My child has a better than average natural ability to learn.	1. Strongly disagree<br>2. Disagree<br>3. Somewhat disagree<br>4. Somewhat agree<br>5. Agree<br>6. Strongly agree	
prcr03	If my child gets a poorer mark in an exam than usual, they will likely raise their achievement to the usual level the next time.	1. Strongly disagree<br>2. Disagree<br>3. Somewhat disagree<br>4. Somewhat agree<br>5. Agree<br>6. Strongly agree	
prcr04	My child can handle many things at a time.	1. Strongly disagree<br>2. Disagree<br>3. Somewhat disagree<br>4. Somewhat agree<br>5. Agree<br>6. Strongly agree	
prcr05	My child has possibilities to develop in many directions.	1. Strongly disagree<br>2. Disagree<br>3. Somewhat disagree<br>4. Somewhat agree<br>5. Agree<br>6. Strongly agree	
prcr06	My child is determined.	1. Strongly disagree<br>2. Disagree<br>3. Somewhat disagree<br>4. Somewhat agree<br>5. Agree<br>6. Strongly agree	
prcr07	When my child is in a difficult situation, they can usually find their way out of it.	1. Strongly disagree	

PID:	Date: DD MMM YY	School Code:	Initials of Data Collector:

		2. Disagree<br>3. Somewhat disagree<br>4. Somewhat agree<br>5. Agree<br>6. Strongly agree	
prcr08	When my child makes plans they follow through with them.	1. Strongly disagree<br>2. Disagree<br>3. Somewhat disagree<br>4. Somewhat agree<br>5. Agree<br>6. Strongly agree	
prcr09	My child usually manages one way or another.	1. Strongly disagree<br>2. Disagree<br>3. Somewhat disagree<br>4. Somewhat agree<br>5. Agree<br>6. Strongly agree	
prcr10	My child has enough energy to do what they have to do.	1. Strongly disagree<br>2. Disagree<br>3. Somewhat disagree<br>4. Somewhat agree<br>5. Agree<br>6. Strongly agree	
prcr11	My child keeps interested in things.	1. Strongly disagree<br>2. Disagree<br>3. Somewhat disagree<br>4. Somewhat agree<br>5. Agree<br>6. Strongly agree	
prcr12	My child has self-discipline.	1. Strongly disagree<br>2. Disagree<br>3. Somewhat disagree<br>4. Somewhat agree<br>5. Agree<br>6. Strongly agree	
prcr13	My child feels proud that they have accomplished things.	1. Strongly disagree<br>2. Disagree	

PID:	Date: DD MMM YY	School Code:	Initials of Data Collector:

		3. Somewhat disagree<br>4. Somewhat agree<br>5. Agree<br>6. Strongly agree	
prcr14	My child is friends with themself.	1. Strongly disagree<br>2. Disagree<br>3. Somewhat disagree<br>4. Somewhat agree<br>5. Agree<br>6. Strongly agree	
prcr15	My child usually takes things in stride.	1. Strongly disagree<br>2. Disagree<br>3. Somewhat disagree<br>4. Somewhat agree<br>5. Agree<br>6. Strongly agree	
prcr16	My child relies on themself.	1. Strongly disagree<br>2. Disagree<br>3. Somewhat disagree<br>4. Somewhat agree<br>5. Agree<br>6. Strongly agree	
prcr17	My child can usually find something to laugh about.	1. Strongly disagree<br>2. Disagree<br>3. Somewhat disagree<br>4. Somewhat agree<br>5. Agree<br>6. Strongly agree	
prcr18	My child can usually look at a situation in a number of ways.	1. Strongly disagree<br>2. Disagree<br>3. Somewhat disagree<br>4. Somewhat agree<br>5. Agree<br>6. Strongly agree	

PID:	Date: DD MMM YY	School Code:	Initials of Data Collector:

Section 6: ABOUT YOU

6.1 Social Desirability Scale

Var Name	Item	Response Options	Response
psdsintro01	Listed below are a number of statements concerning personal attitudes and traits. Please indicate whether the statement is true or false as it pertains to you personally.		
psds01	I sometimes feel resentful when I don't get my way.	0. False<br>1. True	
psds02	On a few occasions, I have given up something because I thought too little of my ability to do it.	0. False<br>1. True	
psds03	There have been times when I felt like rebelling against people in authority even though I knew they were right.	0. False<br>1. True	
psds04	No matter who I'm talking to, I'm always a good listener.	0. False<br>1. True	
psds05	I can remember "playing sick" to get out of something.	0. False<br>1. True	
psds06	There have been occasions when I have taken advantage of someone.	0. False<br>1. True	
psds07	I'm always willing to admit it when I make a mistake.	0. False<br>1. True	
psds08	I sometimes try to "get even" rather than "forgive and forget."	0. False<br>1. True	
psds09	When I don't know something, I don't mind admitting it at all.	0. False<br>1. True	
psds10	I am sometimes irritated by people who ask favors of me.	0. False<br>1. True	
psds11	I have never said something that hurt someone's feelings on purpose.	0. False<br>1. True	

Section 7: Contamination

Var Name	Item	Response Options	Response

PID:	Date: D D M M M Y Y	School Code:	Initials of Data Collector:

contamin01	Indicate which SUPPER materials other than those that were provided directly to you by the SUPPER Project Team you have received, been given, or heard of?<br><br>Indicate all those that apply.	0. I have not received, been given or heard of any SUPPER materials other than those directly given to me.<br>1. Handbooks and inserts<br>2. Text messages<br>3. Websites and resources<br>4. Magnets<br>5. Interventionist materials and action plan	
