# Supplementary material for: Randomized controlled trial to test the efficacy of a brief, communication-based, substance use preventive intervention for parents of adolescents: Protocol for the SUPPER Project (Substance Use Prevention Promoted by Eating family meals Regularly)
Source: PLoS One. 2022 Feb 2;17(2):e0263016. doi: 10.1371/journal.pone.0263016 (PMC8809599; doi:10.1371/journal.pone.0263016)
Supplement: S5 File — (DOCX) [file pone.0263016.s006.docx]

**PROTOCOL TITLE:**

A Brief Substance Use Preventive Intervention for Parents of Youth

**PRINCIPAL INVESTIGATOR (PI):**

**Name**: Dr. Margie Skeer

**Department**: Public Health and Community Medicine

**Are you a student**? Yes  No

*If Yes, you must designate a Faculty Advisor below.*

**FACULTY ADVISOR (required for Student PIs):**

**Name**: N/A

**Department**: N/A

**VERSION NUMBER/DATE**

Version: 13

Date: 12 Jan 2021

# Purpose of the study:

The purpose of this study is to test the efficacy of a brief, communication-based, substance use preventive intervention for parents of pre/early adolescents. A parallel-group randomized controlled trial (RCT) to test the primary hypotheses that, relative to parents in the comparison condition, parents receiving the intervention, over the study period, will have, on average: 1) greater quality of interactions at meals, 2) a greater number of conversations about substances, and 3) higher quality of content in their conversations with their children about sustance use. A companion primary hypothesis will be tested to determine if children of parents randomized to the intervention have fewer intentions and reduced willingness to use substances, reduced affiliation with substance-using peers,and increased negative attitudes and expectancies about substance use in contrast to children of parents in the comparison condition at study follow-up. We will also test a secondary aim’s hypothesis, that children of parents assigned to the intervention condition will have a delayed initiation of substance use compared to the comparison condition.

# Background / Literature Review / Rationale for the study:

Universal, family-based substance use preventive interventions have been shown to be efficacious, but programs that have been most effective are resource intensive and require extensive time and effort for program staff and participants. Therefore, an approach to universal substance misuse prevention is needed that is effective, resource-efficient, able to be disseminated easily, and sustainable.

# Participant Population:

We propose to enroll parents of pre/early-adolescents (grades 5-7) with their child who live and attend school in Massachusetts.

Inclusion criteria include: 1) the parent must have a child between 5th-7^th^ grades at the start of the project; 2) the parent must be the custodial parent, living with the child at least 50% of the time (as in the case of joint custody); and 3) parental consent and child assent are obtained. If a parent has more than one child that meets these criteria, the target child to participate in the intervention will be randomly chosen by research staff.

Exclusion criteria include:(1) the child has self-identified developmental disabilities who would have difficulty understanding the baseline assessment, and 2) the parent or child does not adequately speak, read, and comprehend English or Spanish well enough to complete study procedures.

# Special Populations:

|  | Children |
| --- | --- |
|  | Fetuses/Neonates |
|  | Prisoners |
|  | Members of the military |
|  | Non-English speakers |
|  | Those unable to read (illiterate) |
|  | Employees of the researcher |
|  | Students of the researcher |
|  | Adults lacking capacity to consent and/or adults with diminished capacity to consent, including, but not limited to, those with acute medical conditions, psychiatric disorders, neurologic disorders, developmental disorders, and behavioral disorders |
|  | Disadvantaged in the distribution of social goods and services such as income, housing, or healthcare |
|  | Fear of negative consequences for not participating in the research (e.g. institutionalization, deportation, disclosure of stigmatizing behavior) |
|  | Approached for participation in research during a stressful situation such as emergency room setting, childbirth (labor), etc. |

# Research Locations and Sample Size:

- 1. **Research Locations**

We will be working with schools in Massachusetts. We will obtain approval from schools prior to beginning any data collection. Once school district approval is received, data collection will begin and we will submit these letters of approval to the IRB to maintain an up-to-date list of school districts. Tufts University School of Medicine and Participant Homes will also serve as locations for data collection and intervention sessions.

***5.2* Sample Size**

The number of participants from each school will depend upon the number willing and available to participate in each school district, and the total number of participants from all sites combined shall not exceed 500.

# Procedures Involved:

Eligible, consenting parents and their assenting child will complete baseline assessments related to their family practice of eating meals together, communication skills, parenting styles, and their own substance use. Parents will then be randomly assigned to one of two conditions: 1) intervention, or 2) comparison. Parents and children will then be followed at four follow-up points at 3, 6, 12, and 18 months.

Brief Preventive Intervention Condition. While the intervention will only be given to the parent participants, the parent and their child will be administered survey-and video-based baseline and follow-up assessments. After parent participants complete the baseline assessments, they will be given a handbook specific to the gender of their child, written on an eighth-grade reading level that provides information and advice on six domains: 1) background on adolescent SU, including up-to-date statistics on the prevalence of use of different substances by youth and effects of SU on the developing brain; 2) parent-child communication, including the importance of open communication, monitoring, and ways to communicate effectively; 3) eating at least five meals per week with their children, including information on which meals parents should eat with their children, what to avoid doing during meals (for example, watching television, or using phones to talk, text, or search the internet), and alternatives if eating meals together is not possible; 4) parent-child communication specifically about the harms of SU, including understanding why teenagers use substances, finding out what their children already know, providing facts about substances, ways to explain why their children should not use substances, setting clear parental expectations, setting a good example, addressing problems with permissive parenting around SU, and sample conversation starters; 5) the role of substance-using peers with respect to adolescent substance use and effective peer management strategies and 6) additional resources, which are available through a study website with links to resources for additional information (to be printed out for parents without internet access).The handbook will be available on a portable USB flash drive and on the study website. The primary differences in the content of the handbooks by gender are in: 1) the statistics about SU, 2) the section on reasons why teens choose to use substances, which are different for adolescent boys and girls, 3) ways to help boys and girls meet distinct needs without substances; and 4) gender-specific resources available to parents.

The intervention will comprise of two components: in-person or via video conference, and home-based. Parents will be instructed to read the handbook in its entirety before the in-person/video conference session. One-to-two weeks after receiving the handbook, parents will participate in a one-hour session with an interventionist where the main points in the handbook will be reviewed. Parents will also be given the opportunity to ask questions to increase their competencies around the handbook suggestions. Additionally, with guidance from the interventionist, parents will fill out an action plan on how to make changes in family meals and communication about substances with their child. The interventionist will also provide parents with a referral packet, which will include resources for child and adult mental and physical health services, substance-related services, and will have insurance required, as well as free provider options. In response to parent feedback from the uncontrolled pilot indicating a desire to have more contact with the study interventionist, which we tested in the pilot RCT, two weeks after the in-person session, participants will have a half-hour follow-up phone call with the same study interventionist to go over the action plan and answer any questions that have arisen after parents have had an opportunity to implement changes with their children. Communication specialists will conduct the in-person/video conference sessions. For the home-based component, parents will receive two messages each week with reminders and tips that reinforce the information covered in the handbook. Parent participants who do not have their own cell phone will be provided a disposable phone, which will allow access to text messages. Parents who do not know how to text will be given a tutorial during the intervention session. Finally, participants will receive a magnet about the importance of family meals that they will be instructed to put on their refrigerators. Parents and their target child will complete post-intervention 3-, 6-, 12-and 18-month follow-up survey assessments and 3-, 6- and 12-month follow-up video and audio assessments. Weight and height measurements will be taken during baseline for child participants. To minimize the threat of group cross-contamination due to randomization at the individual, rather than school level (see Design Considerations), participants in the intervention condition will be apprised of the importance of not sharing any information about the intervention with other parents during the intervention period. They will be informed that after study completion, all parents in the school will be offered intervention materials. The level of cross-contamination will be assessed by asking parents at the end of the trial if they shared information with or received information from other parents, and through a cross-contamination measure (see Measures).

Comparison Condition. Parents and children in the comparison condition will be administered the baseline survey, video assessments and child weight/height measurement, and the follow-up survey (3, 6, 12, 18M) and video/audio (3, 6, 12, and 18M) assessments on the same schedule as intervention participants. For the comparison condition, after the baseline assessment, parents will receive a handbook on the importance of nutrition and physical activity for children entitled: "Healthy Eating & Physical Activity Across Your Lifespan: Helping your Child –Tips for Parents" with an insert on weight talk that was developed by the research team. The handbook, which is sponsored by the National Institute of Diabetes and Digestive and Kidney Diseases and the Weight Control Information Network, is approximately the same length as the intervention handbook and is available in English and Spanish. They will also receive a magnet with a message about nutrition and exercise. To control for contact time, these participants will also meet in-person/video conference with a study staff member to go over the comparison handbook material and complete an action plan two weeks after receiving the handbook and complete an action plan, as well as have a half hour follow up phone call two weeks after the in-person/video-conference session. Parents will receive two text messages twice per week for 13 weeks with tips and reminders from the comparison handbook.

Duration of participant involvement:

Week 0: Baseline survey, video family meal and audio conversation recordings, and child weight/height measurement

Weeks 1-13: Intervention (Intervention group activities include receiving handbook, in-person/video conference intervention, follow-up phone call, and text-messaging; control group activities include receiving handbook, in-person/video conference meeting, follow-up phone call, and text-messaging.)

Week 13: Post-intervention survey and audio conversation recordings
Week 26: 6-month survey and video family meal and audio conversation recordings
Week 52: 12-month survey and video family meal and audio conversation recordings
Week 78: 18-month survey and audio conversation recordings

Surveys and videos can be completed within +/- 2-week period of the given week. Participants that do not complete the surveys or videos within the given time period will be followed up with and asked to provide missing data. If changes are made to survey instruments after participants have already received the instrument, study staff will treat this as missing data and will contact participants to obtain the missing data.

In situations where participants express interest in continuing with the study but lack time to complete materials for a specific time point, participants will have the option to complete a shortened survey. They will specifically consent/assent to taking this shortened survey and receiving a $5 (3- or 6-month) or $10 (12- or 18-month) Amazon e-gift card. This consent/assent process is completed at the beginning of the shortened survey.

The expected duration of the study is 5 years.

When a participant completes the study, we will complete the following steps:

- Offer the other group’s materials to be emailed or mailed to them
- Send them the completion email and request a response to confirm their acknowledgement of completion
- Send them instructions on what to do with the tablet
- Ask them to complete a form which asks for consent to keep their contact information for potential future studies

We will keep all contact information of adult participants for future study purposes who consent to us keeping their information for this purpose. This contact information will be kept in its own Box folder, separate from any data or other study-related files. For those that do not consent to us keeping their contact information, we will delete their contact information after five years after the cessation of the study as per the protocol (Section 17.0 Data Management and Confidentiality).

Instrumentation

- Questionnaires
- Video: Video observation of family meals and parent-child discussions about substance use
- Audio: intervention in-person/video conferenced sessions will be recorded for quality assurance
- Other: height/weight measurement of children

Online Instrumentation

- The online survey engine REDCap will be used to present the surveys.
  - <https://collaborate.tuftsctsi.org/redcap//index.php?action=myprojects>
- Online recruitment agencies will have their own method of distributing the approved screening and eligibility survey to potential participants, such as a Qualtrics or REDCap survey.
- Participants will be able to complete the entire survey if they abstain from answering certain questions.
- Paper surveys are also available.

Data Analysis Plan

The scientific rigor of this application is reflected in our preliminary analyses, missing data detection and correction procedures, and our data analytic strategies. First, all forms will be checked for missing data and outliers prior to data entry. Attrition effects will be evaluated by testing whether systematic differences exist between participants who complete the research versus those who drop out. We will also examine any pre-existing differences between the two conditions in demographic composition and all measures collected at baseline. Pre-existing group differences in these variables will be accounted for in subsequent analyses, using baseline scores as covariates. Participants will be included in the analysis if they are randomized (i.e., intention -to-treat). Participants who are lost to follow-up or drop out can affect the validity of the group comparisons if: 1) the outcome of interest is related to being lost or dropped, and 2) it is differential between groups. The missing data mechanism will be formally tested to determine whether it is Missing Completely at Random (MCAR) or Missing at Random (MAR). For completeness in assessing the effect of missingness, two additional analyses will be conducted: 1) a ‘per-protocol’ analysis among participants who complete all follow-up surveys; and 2) multiple imputation of five data sets using a discriminant function for binary outcomes and Markov chain Monte Carlo for continuous outcomes, assuming the missing data mechanism is MAR.

Aim 1: To determine the efficacy of the brief intervention on parent outcomes. The proportion of parents who more frequently communicate will be compared between conditions across the follow-ups periods using a repeated measures log-binomial regression analysis (via generalized estimating equations; GEE), with an appropriate covariance structure and adjusting for baseline scores. Covariates will be added to the modeled analyses if differences among baseline characteristics are observed. A test for interaction between condition and follow-up point using the above models will assess whether there is a differential intervention effect across time, taking intervention dose delivered and received into account. To assess quality of mealtime interactions, we will first score the videos according to the IFIRS and then analyze the data using repeated measures analysis of covariance (i.e., a linear mixed model) to compare the two conditions across the follow-ups, using an appropriate covariance structure and adjusting for baseline scores. A similar analysis will be used for the summed scores from the FAsTask, which assess the quality of parent-child conversations about SU.

Aim 2: To examine the effects of the intervention on child SU beliefs, intentions, and willingness, and affiliation with substance-using peers. We will compare the proportion of children with intentions and willingness to use substances, negative SU attitudes and expectancies, and substance-using peers between conditions across the follow-up periods using a repeated measures log-binomial regression analysis (via GEE), with an appropriate covariance structure and adjusting for baseline scores.

Secondary Aim 1: To examine the effects of the intervention on SU initiation. To assess whether children of parents in the intervention condition delay the initiation of SU longer than the comparison condition, we will compare the incidence of initiation using a discrete-time survival analysis via a complementary log-log regression model, as the date of initiation between follow-up time points may not be known exactly.

Exploratory analyses will be run by race and ethnicity. Because it is known that subgroup analyses have less statistical power than the primary analysis, we will conduct exploratory analyses to determine whether the efficacy of the intervention varies by race and ethnicity, to inform future adaptations.

- 1. **Additional Safeguards for Special Populations**:

**Children**

The inclusion of children is necessary as this is a prevention study to conduct research with children. The study is designed as a prevention program for kids and families.

It is possible that situations will arise in which interventionists or other study team members are provided with information by children or families, which they are legally and ethically obligated to disclose. This will be in accordance with the Massachusetts state statutes for making a report for children, as delineated in the following document: https://www.childwelfare.gov/pubpdfs/manda.pdf. According to the statute in the state, “a mandated reporter must report when, in his or her professional capacity, he or she has reasonable cause to believe that a child is suffering physical or emotional injury resulting from:

- Abuse inflicted upon the child that causes harm or substantial risk of harm to the child’s health or welfare, including sexual abuse;
- Neglect, including malnutrition;
- Being a sexually exploited child; and
- Being a human trafficking victim as defined by chapter 233, § 20M.”

Further assessment of parental drug use as child abuse will be assessed as well, in accordance with the following document: https://www.childwelfare.gov/pubpdfs/drugexposed.pdf. Communication with the Tufts University IRB indicated that knowledge of substance use by youth is not necessarily indicative of abuse or neglect, and reporting in those situations without other cause to suspect abuse or neglect would be an unnecessary breach of confidentiality.

If any problems are reported or identified during any point of the study, appropriate referral for services or psychological intervention will be arranged. While the questionnaires do not touch on these topics, it is possible that situations will arise in which interventionists or other study staff members are provided with information by children or families which they are legally and ethically obligated to disclose (i.e., child abuse or neglect, whether or not in the context of parental substance use). All participants are informed in advance through the process of informed consent (or assent for child participants) of the legal and ethical obligations of study staff should these issues arise. Whenever possible, study staff will handle these situations therapeutically. For example, if time limits and safety concerns allow, parents will be informed in advance and provided the opportunity to participate in the mandated report process. Research staff will contact Dr. Spirito, the Co-PI (a clinical psychologist) who will be responsible for any on-site clinical decision-making.

Any adverse events that are observed and/or reported during assessments or intervention sessions will be reported immediately to Dr. Skeer. All adverse events will be reported in writing to the IRB within one week. All serious adverse events will be reported to the Tufts IRB immediately by telephone and by written report within 24 hours of our receipt of information regarding the event. NIH will be informed of any serious adverse events and action taken as a result of the IRB’s recommendations.

SAEs will be reported to the NIDA PO within 24 hours of the event by email. This notification will include a brief explanation of the SAE and when it occurred. A written follow up will be sent within 72 hours of the event and will include information on the date of the event, what occurred, actions taken by project staff, planned follow up (if any), the intervention group/study arm of the affected participant (if relevant), whether the event appears to be related to the intervention, and whether the event affects future participation.

**Non-English speakers**

The inclusion of Spanish speakers (who are non-English speakers) is necessary in order to ensure a sample that is as diverse and representative of the population as possible. All study materials are available in Spanish, and members of the study staff, including interventionists, are fluent in Spanish. Any participant who feels more comfortable speaking, reading, and writing exclusively in Spanish will be able to complete all study procedures entirely in Spanish and will be able to speak Spanish with study staff.

Copies of all Spanish translations will be submitted to the IRB along with the required “Certificate of Translation.”

**Those unable to read (illiterate)**

The inclusion of those unable to read (illiterate) is necessary due to universal nature of the intervention. Given the universal nature of the intervention, we aim to ensure that it is accessible to all people in the areas we will be working. Some school districts in Massachusetts have a high level of parental illiteracy, and it is necessary to ensure that the study is equally accessible for all potential participants.

In order to ensure participants with low or no levels of literacy understand the study fully, study staff will carry out the full consent process for those unable to read (detailed in the consent section of the protocol).

# Investigational Medical Devices:

N/A

# Incomplete Disclosure or Deception:

N/A

# Recruitment Methods:

*Recruitment Techniques*

- Email or web postings
- Letters to professionals/institutions
- Letters to parents/guardians
- Brochures, flyers, and/or pamphlets
- Letters to subjects (information sheet to children)
- Online recruitment
- Snowball recruitment, including but not limited to online recruitment platform
- Other (explain): in person

To obtain a diverse sample of parents of 5th-7^th^ grade students, participants will be recruited from schools in Massachusetts. Recruitment will take place in Years 1-3. Study staff members will recruit participants during the school year via in-person recruitment at the end of the school day and within afterschool programs, open houses, PTA meetings, and flyers put up at the school. School administration will have the ability to request their preferred type of recruitment material(s) to send out or distribute using their preferred methods, including, but not limited to: email blasts, school newsletters, social media, letters sent home to students, presentations, phone calls, automated phone calls, flyers, etc. These requests can include school-specific modifications, if the administration prefers. Whenever possible, we will screen on site. For those who cannot screen on site, study staff will collect contact information and organize a separate time to contact interested parents to be screened and then scheduled for an initial survey. In cases where there is limited time to collect contact information, interested parents will be given flyers instructing them to contact study staff at their convenience. Parent participants will be randomized to the intervention or comparison conditions, but child participants will only complete assessments. The PI, Dr. Margie Skeer, will not participate in active recruiting procedures at Milton Public Schools due to her possible prior relationship with parents and their children to prevent undue coersion (see further details in Section IX. Consent). Process).

In order to recruit outside the school setting, particularly given COVID-19, we are hiring an external recruitment agency to recruit dyads within the school systems in which we are currently working. We have received approval from NIH to use this method beginning June 1, 2020. The agency’s role would entail recruiting eligible families from their lists of contacts, with a specific focus on recruiting a diverse sample. Once eligible and interested participants are identified, our team will receive their contact information. The study team will then contact them and follow all screening and eligibility procedures as currently outlined. This will be done to supplement the above school-based recruiting strategies. As described above (Section 6.0; Online Instrumentation), the agency will use its own platform to distribute the screening and eligibility survey.

In order to increase recruitment numbers, we are using the online recruitment agency’s built-in snowball recruitment method. All current participants recruited through the online platform and those recruited outside the outline platform, but willing to make an account, can send referrals to other families they believe would be interested in participating. For each successful recruitment (i.e. they consent to participating in our study), the recruitment agency provides a standard minimal incentive to the referring participant. We will also use traditional snowball recruitment method techniques to encourage participants to share the opportunity with those they believe may be interested.

# Consent Process:

We would like to request a waiver of documentation (signature) for informed consent and child assent. The study presents no more than minimal risk of harm to the participants. Study procedures include: completing surveys, reading a handbook, receiving text messages, meeting with an interventionist, and recording meals and conversations within participants' own homes which are then uploaded by the participant to a secure Box folder shared with the research team.

The Informed consent/assent process will be completed before the start of the assessment at baseline. The full consent and assent forms will be emailed to all participants to give participants a chance to review it and discuss it with their child. A meeting with study staff will happen for each dyad in order to review the study and consent/assent documents. During this meeting, the study staff will ask questions to gauge comprehension and address questions and concerns. The research staff member will give the participants as much time as needed to ensure that they understand the elements of the consent/assent. Once all the participant dyad's questions have been answered and they agree to be part of the study, the adult particpant will complete a REDCap survey to indicate they consent for themself and their child to be part of the study. The child participant will complete a REDCap survey to indicate they assent to be part of the study. The REDCap survey will have the full consent/assent text with a check box where they will indicate consent/assent in lieu of providing a signature. A copy of their responses will be emailed to them for their records and they will have access to the full consent/assent forms in their email. This process will be the same for remote and in-person meetings.

In order to ensure child assent is linked with adult consent, both consent and assent surveys will be sent from and associated with a singular REDCap ID that is unique to each participant dyad.

Procedure for Consent Process with Participants who are Illiterate

1. A copy of the consent/assent form will be mailed to the participant along with a sheet (see ‘Illiterate Contact Sheet’). Before mailing, a member of the study team will let the participant know that it will be mailed and to look for the SUPPER logo on the envelope. If the participant has an email address, the consent/assent forms will also be emailed.
2. A study team member will organize a meeting – either in-person or via video conference – with the participant at a time after the participant received the consent/assent documents.
3. During this meeting, a study team member will read the ICF and assent to the subject and encourage the subject to ask questions. The research staff member will give the participants as much time as needed to ensure that they understand the elements of the consent/assent.
4. This process will be conducted with a witness (another study team member) present. The witness is to observe the entire process. The witness must be literate.
5. If done in-person, the study staff member will assist the participant to open the REDCap consent/assent form and indicate their consent/assent. If done remotely, the participant must have access to an email address. The link will then be emailed to said address and the participant will be instructed to click on the link to open the survey, scroll to the bottom of the survey, and click to indicate they consent/assent. The witness will be present throughout this procedure.
   1. If visit done in person and/or participant does not have an email – the participant will be asked to make a check mark on their paper consent/assent form to indicate they have consented for their records.
   2. If participant has access to an email, a copy of their response will be emailed to them for their records.
6. At the end of the meeting, the study staff member will remind the participant to use the Illiterate Contact Sheet (i.e. phone number) if they have any questions or concerns.
7. A progress note will be made in the participant’s file indicating the participant is illiterate and the illiterate consent process was followed.

The Project Director and/or study Research Assistants will be responsible for obtaining informed consent. They will trained by the PI or co-I in obtaining informed consent.

The consent form describes all study procedures, including confidentiality and privacy, information about potential risks, discomforts, benefits of participation, and information regarding who they can contact with further questions. It also states that participation is voluntary, that participants may decide not to take part or to withdraw from the study at any time without penalty or loss of any benefit to which they might otherwise be entitled, and that study participation is in no way related to their child’s involvement in school. Furthermore, participants can refuse to answer any question or discuss any topic.

Participants at Milton Public Schools, due to their prior possible relationship with Dr. Margie Skeer, PI, will be informed of her participation in the study during screening and eligibilty and again during the consent/assent procedure. For those that do know Dr. Skeer, they will be asked during the consent/assent procedure their preferences for Dr. Skeer’s level of participation in their data collection and data analysis, especially video recordings. For participants who do not want Dr. Skeer involved, the Project Manager and Research Assistants will complete all data collection and analysis.

Additional information regarding the consent procedure

During family meal video recordings, other non-participants may be present. The other persons are not considered participants because no data is collected about them specifically – only “group interaction” in terms of the overall feel/mood of the meal is observed and coded, not their specific interaction and/or relationship with the participants or others. However, to ensure complete transparency of the purpose of the video recording, the parent/guardian participant is asked to explain the purpose of the study to the non-participants prior to recording. Non-participants then verbally agree to their being recorded by stating their name and relationship to the child participant in the video recording (reference: Family Meal Video Recording Instructions).

Re-consent Process for Existing Participants

In the instance that the consent/assent form(s) changes, all existing participants will be reconsented using the following procedure:

1. A notification will be sent out via email, text message, mail or phone call which includes:
   1. the exact language of the update;
   2. information on how to contact a study staff member in case they have questions; and
   3. Instructions for how to reconsent.
2. If the participant agrees to reconsent, they will be directed to a REDCap survey which has the full, updated consent/assent language. The participant will then be asked to click they consent/assent following the same consent/assent procedures as outlined above. A copy of their responses will be emailed to them for their records.
   1. In order to link all consent/assent forms, consent and assent surveys will be sent from and associated with the singular REDCap ID that is unique to each participant dyad.

Non-English Speaking Participants

All study materials, including consent and assent forms, are available in both English and Spanish. Participants will be asked which language they prefer to use. For participants who prefer Spanish, a Spanish-speaking member of the study team will conduct the consent process in Spanish and will explain all study procedures in Spanish and another Spanish-speaking member of the study team will serve as witness.

Copies of all Spanish translations will be submitted to the IRB along with the required “Certificate of Translation.”

Short-Form Consent Process

N/A

Participants who are not yet adults (minors under 18 years of age):

Standard written assent (child participant) will be obtained. Written parental permission for the child’s participation will be obtained.

Participants will undergo a detailed assent process in which study staff explain all study procedures and answer questions concerning the study and assent process. The research staff member will give the pre-adolescent participants as much time as needed to ensure that they understand the elements of the assent. They will be encouraged to confer with the parent completing the informed consent. They will also be encouraged to address any questions or concerns they may have with the research staff member. The research staff member will ask questions to the participant to gauge comprehension. The assent form describes all study procedures, including confidentiality and privacy, information about potential risks, discomforts, benefits of participation, and information regarding who they can contact with further questions. It also states that participation is voluntary, that participants may decide not to take part or to withdraw from the study at any time without penalty or loss of any benefit to which they might otherwise be entitled, and that study participation is in no way related to their involvement at their school. Furthermore, participants can refuse to answer any question or discuss any topic.

Cognitively Impaired Adults:

N/A

Adults Unable to Consent:

N/A

Certificate of Confidentiality:

The NIH automatically issued a Certificate of Confidentiality (CoC) from the United States Department of Health and Human Services (DHHS) as this was an NIH-funded project using identifiable, sensitive information that was on-going on/after December 13, 2016.

# Compensation:

Parent/child dyads will be compensated $60 for completing the baseline assessments, $60 for the 3-and 6-month follow-up surveys, and $80 for the 12-and 18-month follow-up surveys. Dyads will also receive an tablet in order to record their family meals and their conversations about SU. They will be able to keep the tablet as compensation if all video recordings are completed at each time point. Monetary compensation will be in the form of either cash or an Amazon gift card.

For participants who complete the Brief Specific Aims Only Survey for a given time point will receive either $5.00 (3- or 6-month surveys) or $10.00 (12- or 18-month) Amazon gift card. The Brief Specific Aims Only Survey is approximately 10% the length of the full-length survey; therefore, the compensation is proportional. This is in place of completing the full-length survey for that time point and receiving the usual compensation described above.

Compensation for completion of surveys occurs at each follow-up timepoint. Any participant who withdraws from the study before finishing will keep any compensation up until the point of withdrawal but will not be compensated for study activities they would have completed after the timepoint at which they withdrew.

# Economic Burden:

Participants will be responsible for the cost of travel to any interventionist sessions outside their home. Every effort is made to schedule sessions at a location convenient for the participant (e.g. home, work). All activities can also be completed virtually.

# Recording with Audio, Video, or Photographs

Video and audio recordings will be used for direct observation and assessment of conversations about substance use and family meals, as indicated in the specific aims of the study. Intervention sessions will be audio recorded for quality assurance.

The recordings will not be shown to anyone other than research staff. For coding training purposes an IFIRS consultant will have access to a select few participant family meal video recordings. These videos will be shared through Box and only the consultant will have access to view and code videos for the sole purpose of training the SUPPER team. The consultant will provide fee-for-service training to the team and will not be involved in any othe aspects of the study including data collection and analysis.

Participants will be instructed to only use first names on the videos. The recordings will be only be used for coding, data analysis, and training purposes. The recordings are uploaded by the participants to a secure Box folder shared with the study staff, and the recordings are then moved to a secure Box folder only accessible by certain study staff members. Recordings are also backed up on a secure, password-protected external hard drive which is stored in a locked cabinet.

For participants from Milton Public Schools, their preferences specified during the consent/assent period for Dr. Margie Skeer, PI, to be involved in the analysis of their video recordings will be honored.

# Potential Benefits to Participants:

*Potential benefits to participants*

All individuals participating in this research will benefit by having the opportunity to learn more about ways to prevent substance use, as well as possible increased positive family interactions. All families will also receive materials on substance use, as well as services and resources available in their area. Participants in the uncontrolled pilot as well as those randomized to the experimental condition will receive a handbook specific to the gender of their target child that provides tools to increase family communication and monitoring, and ultimately prevent the use and misuse of substances among their children, free of charge. In addition to the handbook, parents will receive a magnet encouraging family meals. The resources from the handbooks will be available on a study specific website that, while publically available in that anyone can access it (i.e., it is not password protected), is developed for the purposes of the study and it is not accessible through any larger search engines (e.g., Google, Bing, etc.). Parents in the comparison condition of the pilot RCT will be offered a copy of the handbook after the completion of the study to obtain the intervention material. The opportunity to speak to a project staff member during the follow-up period and discuss concerns is an additional benefit of the project.

*How the benefits listed above outweigh the risks inherent in the research.*

The risks are minimal to participants. Improvements in parent-child communication and reductions in pre-adolescent substance use initiation would be significant benefits to parents.

# Risks to Participants:

*Potential risks to participants*

There is risk that parent participants in the intervention group will feel embarrassed or uncomfortable, particularly while discussing issues of a personal nature, such as their family environment. In addition, all participants may feel embarrassed or uncomfortable disclosing issues of a personal nature during the assessments, such as their family environment or regarding behavior related to alcohol and drug use.

*Risks to family, school, social group, or place of employment.*

There are no anticipated risks to family, school, social group, or place of employment.

*Does this research qualify as minimal risk or greater than minimal risk?*

This research qualifies as minimal risk. The pilot trial was a minimal risk study (expedited) and procedures are similar. The intervention will focus on parent-child communication and parental engagement. We will ask about risk behaviors in the pre-adolescent questionnaire (substance use), but that in and of itself does not constitute risk.

*Minimizing potential risks*

Potential risk will be minimized by strictly adhering to the guidelines for research outlined by the Tufts University IRB, Massachusetts state law, the Federal Health Insurance Portability and Accountability Act of 1996 and its regulations (“HIPAA”), and the DHHS Federal Policy for the Protection of Human Subjects (45 CFR Part 46 Subpart D). This will include a coded identification system that will be used whereby families are assigned a unique study ID that will be placed on their study assessments and recorded data. Research data, as mentioned, will be accessible only to research staff and all electronic data will be password protected.

Because of the nature of the study, researchers will be transporting confidential information to and from participants' homes and the Tufts University School of Medicine. To ensure confidentiality of the information being gathered (consent forms, video-recorded data, computer-based assessments) researchers will carry lock boxes from each location. Data obtained during the period of the in-home observation (recorded data) will be uploaded directly to Box, and once transferred, erased off the tablet. All written and recorded data will be stored in a locked file cabinet that is behind a locked door. All electronic data will be stored in in the data collection software server and in Box.

As with the pilot project, this study will be collecting identifiable, sensitive information. The NIH has issued a Certificate of Confidentiality (CoC) in order to further protect research participants’ identities and research data from subpoena or court order. This further protects highly vulnerable youth, such as those in CYF care. Participant confidentiality will be breeched only to protect the safety and welfare of research participants and only in accordance with state and federal law. See “Data Safety and Monitoring Plan” for a detailed description of the data and safety monitoring of the research as well as plans for adverse event reporting.

If any problems are reported or identified during any point of the study, appropriate referral for services or psychological intervention will be arranged. While the questionnaires do not touch on these topics, it is possible that situations will arise in which interventionists or other study staff members are provided with information by children or families which they are legally and ethically obligated to disclose (i.e., child abuse or neglect, whether or not in the context of parental substance use). All participants are informed in advance through the process of informed consent (or assent for child participants) of the legal and ethical obligations of study staff should these issues arise. Whenever possible, study staff will handle these situations therapeutically. For example, if time limits and safety concerns allow, parents will be informed in advance and provided the opportunity to participate in the mandated report process. Research staff will contact Dr. Spirito, the Co-PI (a clinical psychologist) who will be responsible for any on-site clinical decision-making.

Participants will be free to terminate their participation in the preventive intervention study at any time. To further ensure confidentiality, no names, only identification codes, will be used in presenting data in lectures, seminars, and papers. No names will be released without written consent of the parents. Participating families will be given full access to their data and professional interpretation if requested after completion of the study.

# Withdrawal of Participants:

Participants are free to withdraw at any point. When a participant indicates they would like to withdraw, the study team coordinates return of study materials (specifically the tablet) and documents participant’s written request for withdrawal.

All data submitted by participants prior to withdrawal will be used in analysis.

Participants will not be withdrawn without their consent.

# Data Management and Confidentiality:

The study data (surveys, video and audio recordings) will be collected and entered by the Project Coordinator and Research Assistants. All physical study records will be kept confidential and stored in locked cabinets in the study team’s office; study records will be identified by an ID number only, and a link between names and ID numbers will be kept separately under lock and key; locator information and informed consent and assent will be stored separately from study assessment documents. The soft copy data will be stored on Box and a secure, password-protected external hard drive stored in a locked cabinet, and only accessibly by the Principal Investigator, Project Coordinator, and Research Assistants. Box is a HIPPA compliant cloud storage. Survey data will be stored in REDCap and paper forms will be stored in a locked filing cabinet.

The Principal Investigator will maintain all study documentation for at least five years after the completion of the study. Recordings will be reviewed by a member of the research team to ensure that there is no identifying information in the recordings. The recordings will only be accessible to study and training staff.

We will keep all contact information of participants who consent to us keeping it for potential future studies in its own Box folder, separate from any study data or other study-related files. For those that do not consent, we will delete the contact information after five years.

# Provisions to Protect the Privacy and Confidentiality of Participants and the Research Data:

All study records will be kept confidential and stored in locked cabinets in research staff office; study records will be identified by an ID number only, and a link between names and ID numbers will be kept separately under lock and key; locator information and informed consent and assent will be stored separately from study assessment documents. The soft copy data will be stored on Box.

The Principal Investigator will maintain all study documentation for at least five years after the completion of the study. Recordings will be reviewed by a member of the research team to ensure that there is no identifying information in the recordings. All video and audio recordings will be kept for at least five years after the completion of the study.

# Provisions to Monitor the Data to Ensure the Safety of Subjects:

N/A

# Compensation for Research-Related Injury:

N/A

# Data Sharing and Specimen Banking:

For coding training purposes an IFIRS consultant will have access to three participant family meal video recordings. These videos will be shared through Box and only the consultant will have access to view and code videos for the sole purpose of training the SUPPER team. The consultant will provide fee-for-service training to the team and will not be involved in any othe aspects of the study including data collection and analysis.

A description of this study will be posted on a public website, http://ClinicalTrials.gov, and summary results of this study will be posted on this website at the conclusion of the research, as required by the National Institutes of Health (NIH), the study sponsor. Only deidentified participant data will be posted.

The final dataset will include self-reported demographic and behavioral data from quantitive codings from audio and video recordings as well las quantitative surveys with the participants. The final dataset will be stripped of identifiers prior to release for sharing and we will make the data and associated documentation available to users only under a data-sharing agreement that provides for: (1) a commitment to using the data only for research purposes and not to identify any individual participant; (2) a commitment to securing the data using appropriate computer technology; and (3) a commitment to destroying or returning the data after analyses are completed.

# International Research:

N/A

# Multiple sites:

This is a collaborative study with co-investigators and consultants from Brown and the University of Minnesota. Data collection is only occurring under the supervision of the Principal Investigator in Massachusetts.

# Reliance Agreements/Single IRB:

This is a collaborative study with co-investigators and consultants from Brown and the University of Minnesota. Smart IRB for Brown University and University of Minnesota. Smart IRB agreements exist for all co-investigators and consultants at Brown and the University of Minnesota.

# Qualifications to Conduct Research and Resources Available:

Principal Investigator / Co-Investigators:

PhD and body of research in prevention, substance use, or relevant academic field

Project Manager:

Master’s Degree in Public Health or relevant field

Consultants:

Demonstrated expertise and specialty in their area

Research Assistants:

Master’s Degree in Public Health or relevant field

Undergraduate Research Assistants:

Demonstrated interest in prevention research

Data Collection Assistants:

Demonstrated interest in prevention research
