# Supplementary material for: Randomized controlled trial to test the efficacy of a brief, communication-based, substance use preventive intervention for parents of adolescents: Protocol for the SUPPER Project (Substance Use Prevention Promoted by Eating family meals Regularly)
Source: PLoS One. 2022 Feb 2;17(2):e0263016. doi: 10.1371/journal.pone.0263016 (PMC8809599; doi:10.1371/journal.pone.0263016)
Supplement: S6 File — (DOCX) [file pone.0263016.s007.docx]

Abstract

Randomized control trial study protocol of a brief, family-centered, substance use preventive intervention for parents of pre-adolescents

**Margie R. Skeer, ScD, MPH, MSW**^1^, Rachael A. Sabelli, MS^1^, Katherine M. RancaÃ±o, MS^2^, Michelle A. Lee-Bravatti, MS, MPH^1^, Emma C. Ryan, MPH^1^, Misha Eliasziw, PhD^1^ and Anthony Spirito, PhD^3^, (1) Tufts University School of Medicine, Boston, MA, (2) The Gerald J. and Dorothy R. Friedman School of Nutrition Science and Policy at Tufts University, Boston, MA, (3) Alpert Medical School, Brown University, Providence, RI

APHA's 2020 VIRTUAL Annual Meeting and Expo (Oct. 24 - 28)

Family-based substance-use prevention programs that target the general population have been shown to be efficacious. However, they are often resource- and time-intensive, thus reducing potential program sustainability. To address this, we developed a brief, resource-efficient intervention with a goal of future implementation in school systems; preliminary efficacy was established in a NIDA-funded pilot study (R34). We are currently implementing a full-scale efficacy trial of the intervention (R01) with 500 parents/guardians of 5^th^-7^th^-graders in the Greater Boston area. Parent-child dyads recruited from participating schools are randomized to the intervention or comparison condition using block-urn randomization. The 13-week intervention focuses on parental engagement by promoting family meals and improving parent-child communicationâ€”in general and about substance use specifically. Parent participants receive a short handbook, attend two meetings with a communication specialist (one in person and one by phone), and receive two text messages/week. The study was designed with an attention control condition, where parent participants receive the same dose, but with content focused on physical activity, nutrition and reducing weight-based teasing in families. All study materials and activities are available in English and Spanish. Parents and children provide data over 18 months (baseline, 3-, 6-, 12-, 18-month follow up) with a study-provided iPad to submit videos of family meals, audio recordings of prompted parent-child conversations about substance use through adapted FAsTask methodology, and quantitative surveys. The primary outcomes are: 1) quantity and quality of substance-use conversations, 2) quality of family-meal interactions, and 3) child substance-use attitudes and expectancies, affiliation with substance-using peers, and intentions/willingness to use. The secondary aim is child substance-use initiation.

Planning of health education strategies, interventions, and programs

Public health or related research

Social and behavioral sciences
