# Supplementary material for: Randomized controlled trial to test the efficacy of a brief, communication-based, substance use preventive intervention for parents of adolescents: Protocol for the SUPPER Project (Substance Use Prevention Promoted by Eating family meals Regularly)
Source: PLoS One. 2022 Feb 2;17(2):e0263016. doi: 10.1371/journal.pone.0263016 (PMC8809599; doi:10.1371/journal.pone.0263016)
Supplement: Peer review history

# Editor report: Decision Letter 0

**DOI:** 10.1371/journal.pone.0263016.r001

**Authors:** Lorena Verduci

11 Jan 2022

Randomized controlled trial to test the efficacy of a brief, communication-based, substance use preventive intervention for parents of adolescents: Protocol for the SUPPER Project (Substance Use Prevention Promoted by Eating family meals Regularly)

PONE-D-21-23964

Dear Dr. Skeer,

We’re pleased to inform you that your manuscript has been judged scientifically suitable for publication and will be formally accepted for publication once it meets all outstanding technical requirements.

Within one week, you’ll receive an e-mail detailing the required amendments. When these have been addressed, you’ll receive a formal acceptance letter and your manuscript will be scheduled for publication.

An invoice for payment will follow shortly after the formal acceptance. To ensure an efficient process, please log into Editorial Manager at http://www.editorialmanager.com/pone/, click the 'Update My Information' link at the top of the page, and double check that your user information is up-to-date. If you have any billing related questions, please contact our Author Billing department directly at authorbilling@plos.org.

If your institution or institutions have a press office, please notify them about your upcoming paper to help maximize its impact. If they’ll be preparing press materials, please inform our press team as soon as possible -- no later than 48 hours after receiving the formal acceptance. Your manuscript will remain under strict press embargo until 2 pm Eastern Time on the date of publication. For more information, please contact onepress@plos.org.

Kind regards,

Lorena Verduci

Staff Editor

PLOS ONE

Journal Requirements:

1. Please include additional information regarding the survey or questionnaire used in the study and ensure that you have provided sufficient details that others could replicate the analyses. For instance, if you developed a questionnaire as part of this study and it is not under a copyright more restrictive than CC-BY, please include a copy, in both the original language and English, as Supporting Information.

2. We note that you have referenced (ie Bewick et al. [5]”) which has currently not yet been accepted for publication. Please respond by return e-mail with a copy of your updated manuscript to include to remove this from your References and amend this to state in the body of your manuscript: (ie “Bewick et al. [Unpublished]”) as detailed online in our guide for authors

http://journals.plos.org/plosone/s/submission-guidelines#loc-reference-style. We can then upload this to your submission on your behalf.

# Editor report: Acceptance letter

**DOI:** 10.1371/journal.pone.0263016.r002

**Authors:** Lorena Verduci

24 Jan 2022

PONE-D-21-23964 

Randomized controlled trial to test the efficacy of a brief, communication-based, substance use preventive intervention for parents of adolescents: Protocol for the SUPPER Project (Substance Use Prevention Promoted by Eating family meals Regularly) 

Dear Dr. Skeer:

I'm pleased to inform you that your manuscript has been deemed suitable for publication in PLOS ONE. Congratulations! Your manuscript is now with our production department. 

If your institution or institutions have a press office, please let them know about your upcoming paper now to help maximize its impact. If they'll be preparing press materials, please inform our press team within the next 48 hours. Your manuscript will remain under strict press embargo until 2 pm Eastern Time on the date of publication. For more information please contact onepress@plos.org.

If we can help with anything else, please email us at plosone@plos.org. 

Thank you for submitting your work to PLOS ONE and supporting open access. 

Kind regards, 

PLOS ONE Editorial Office Staff

on behalf of

Dr Lorena Verduci 

Staff Editor

PLOS ONE
